# An improved assembly and annotation of the allohexaploid wheat genome identifies complete families of agronomic genes and provides genomic evidence for chromosomal translocations

**DOI:** 10.1101/gr.217117.116

**Published:** 2017-05

**Authors:** Bernardo J. Clavijo, Luca Venturini, Christian Schudoma, Gonzalo Garcia Accinelli, Gemy Kaithakottil, Jonathan Wright, Philippa Borrill, George Kettleborough, Darren Heavens, Helen Chapman, James Lipscombe, Tom Barker, Fu-Hao Lu, Neil McKenzie, Dina Raats, Ricardo H. Ramirez-Gonzalez, Aurore Coince, Ned Peel, Lawrence Percival-Alwyn, Owen Duncan, Josua Trösch, Guotai Yu, Dan M. Bolser, Guy Namaati, Arnaud Kerhornou, Manuel Spannagl, Heidrun Gundlach, Georg Haberer, Robert P. Davey, Christine Fosker, Federica Di Palma, Andrew L. Phillips, A. Harvey Millar, Paul J. Kersey, Cristobal Uauy, Ksenia V. Krasileva, David Swarbreck, Michael W. Bevan, Matthew D. Clark

**Affiliations:** 1 Earlham Institute, Norwich, NR4 7UZ, United Kingdom;; 2 John Innes Centre, Norwich, NR4 7UH, United Kingdom;; 3 ARC Centre of Excellence in Plant Energy Biology, The University of Western Australia, Crawley Western Australia 6009, Australia;; 4 EMBL European Bioinformatics Institute, Hinxton, CB10 1SD, United Kingdom;; 5 Plant Genome and Systems Biology, Helmholtz Center Munich, 85764 Neuherberg, Germany;; 6 University of East Anglia, Norwich, NR4 7TJ, United Kingdom;; 7 Rothamsted Research, Harpenden, AL5 2JQ, United Kingdom;; 8 The Sainsbury Laboratory, Norwich, NR4 7UH, United Kingdom

## Abstract

Advances in genome sequencing and assembly technologies are generating many high-quality genome sequences, but assemblies of large, repeat-rich polyploid genomes, such as that of bread wheat, remain fragmented and incomplete. We have generated a new wheat whole-genome shotgun sequence assembly using a combination of optimized data types and an assembly algorithm designed to deal with large and complex genomes. The new assembly represents >78% of the genome with a scaffold N50 of 88.8 kb that has a high fidelity to the input data. Our new annotation combines strand-specific Illumina RNA-seq and Pacific Biosciences (PacBio) full-length cDNAs to identify 104,091 high-confidence protein-coding genes and 10,156 noncoding RNA genes. We confirmed three known and identified one novel genome rearrangements. Our approach enables the rapid and scalable assembly of wheat genomes, the identification of structural variants, and the definition of complete gene models, all powerful resources for trait analysis and breeding of this key global crop.

Improvements in sequencing read lengths and throughput have enabled the rapid and cost-effective assembly of many large and complex genomes (Gnerre et al. 2011; Lam et al. 2011). Comparisons between assembled genomes have revealed many classes of sequence variation of major functional significance that were not detected by direct alignment of sequence reads to a common reference (The 1000 Genomes Project Consortium 2010; Gan et al. 2011; Bishara et al. 2015). Therefore, accurate comparative genomics requires that genome sequences are assembled prior to alignment, but in many eukaryotic genomes, assembly is complicated by the presence of large tracts of repetitive sequences (Treangen and Salzberg 2012; Chaisson et al. 2015) and the common occurrence of genome duplications, for example, in polyploids (Blanc and Wolfe 2004; Berthelot et al. 2014).

Recent innovations in sequence library preparation, assembly algorithms, and long-range scaffolding have dramatically improved whole-genome shotgun assemblies from short-read sequences. These include PCR-free library preparation to reduce bias (Aird et al. 2011), longer sequence reads, and algorithms that preserve allelic diversity during assembly (Weisenfeld et al. 2014). Short-read assemblies have been linked into larger chromosome-scale scaffolds by Hi-C in vivo (Lieberman-Aiden et al. 2009) and in vitro (Putnam et al. 2016) chromatin proximity ligation, as well as by linked-read sequencing technologies (Mostovoy et al. 2016; Weisenfeld et al. 2016). Although it is more expensive than short-read sequencing approaches, single-molecule real-time (SMRT) sequencing improved the contiguity and repeat representation of mammalian (Pendleton et al. 2015; Gordon et al. 2016; Bickhart et al. 2017) and diploid grass genomes (Zimin et al. 2017). SMRT technologies are also being used to generate the complete sequence of transcripts, increasing the accuracy of splicing isoform definition (Abdel-Ghany et al. 2016).

The assembly of the 17Gb allohexaploid genome of bread wheat (*Triticum aestivum*) has posed major difficulties, as it is composed of three large, repetitive, and closely related genomes (Moore et al. 1995). Despite progressive improvements, an accurate and near-complete wheat genome sequence assembly and corresponding high-quality gene annotation has not yet been generated. Initial whole-genome sequencing used orthologous Poaceae protein sequences to generate highly fragmented gene assemblies (Brenchley et al. 2012). A BAC-based assembly of Chromosome 3B provided major insights into wheat chromosome organization (Choulet et al. 2014). Illumina sequencing and assembly of flow-sorted chromosome arm DNA (Chromosome Survey Sequencing [CSS]) identified homoeologous relationships between genes in the three genomes, but the assemblies remained highly fragmented (The International Wheat Genome Sequencing Consortium 2014). Recently, a whole-genome shotgun sequence of hexaploid wheat was assembled and anchored, though not annotated, using an ultradense genetic map (Chapman et al. 2015). The assembly contained ∼48.2% of the genome with contig and scaffold N50 lengths of 8.3 and 25 kb, respectively.

Here we report the most complete and accurate sequence assembly and annotation to date of the allohexaploid wheat reference accession, Chinese Spring (CS42). Our approach is open source, rapid, and scalable and enables a more in-depth analysis of sequence and structural variation in this key global crop.

## Results

### DNA library preparation and sequencing

We aimed to reduce bias and retain maximum sequence complexity by using unamplified libraries for contig generation (Kozarewa et al. 2009) and to improve scaffolding by using precisely sized mate-pair libraries (Heavens et al. 2015). Libraries were sequenced using Illumina paired-end (PE) 250-bp reads to distinguish closely related sequences. In total, 1.1 billion PE reads were generated to provide 33× sequence coverage of the CS42 genome (Supplemental Information S1; Supplemental Table S4.1). For scaffolding, long mate-pair (LMP) libraries with insert sizes ranging from 2480–11,600 bp provided 53× sequence coverage, and Tight, Amplification-free, Large insert PE Libraries (TALL) with an insert size of 690 bp provided 15× sequence coverage (Supplemental Information S1; Supplemental Table S4.2).

### Genome assembly

Nearly 3 million contigs (of length >500 bp) were generated using the w2rap-contigger (Clavijo et al. 2017) with an N50 of 16.7 kb (Supplemental Information S1; Supplemental Table S4.3). After scaffolding using SOAPdenovo (Luo et al. 2012), the assembly contained 1.3 million sequences with an N50 of 83.9 kbp. The TGACv1 scaffolds were classified to chromosome arms using raw CSS reads (The International Wheat Genome Sequencing Consortium 2014) and subsequently screened with a two-tiered filter based first on their length and their *k*-mer content (see Supplemental Information S1, section S4.5). The approach removed short, redundant sequences from the assembly minimizing the loss of unique sequence content, leading to an increase in scaffold N50 to 88.8 kb. Contig accuracy was assessed by mapping links from the 11-kb LMP library, which was not used in the contig assembly. Breaks in the linkage at different mate-pair mapping coverages only affected a very small portion of the content and did not reduce N50 contiguity significantly (Supplemental Information S1; Supplemental Figs. S4.4, S4.5). Supplemental Tables S4.5 and S4.6 in S1 show that 91.1% of TGACv1 genes were correctly assigned to Chromosome 3B, with no discrepancies in gene order identified.

The genome of a synthetic wheat line W7984 was previously assembled with an improved version of meraculous (Chapman et al. 2011) using 150-bp PE libraries with varying insert sizes, for a combined genome coverage of 34.3×, together with 1.5- and 4-kb LMP libraries for scaffolding (Chapman et al. 2015). This contig assembly, with an N50 of 8.3 kb, covered 8 Gb of the genome while the scaffold assembly covered 8.21 Gb with an N50 of 24.8 kb. In comparison, the TGACv1 assembly represents ∼80% of the 17-Gb genome, a 60% improvement in genome coverage. The contiguity of the TGACv1 assembly, as measured by scaffold N50 values, is 3.7-fold greater than that of the W7984 assembly and 30 times that of the CSS assembly (Table 1; The International Wheat Genome Sequencing Consortium 2014).

**Table 1.**
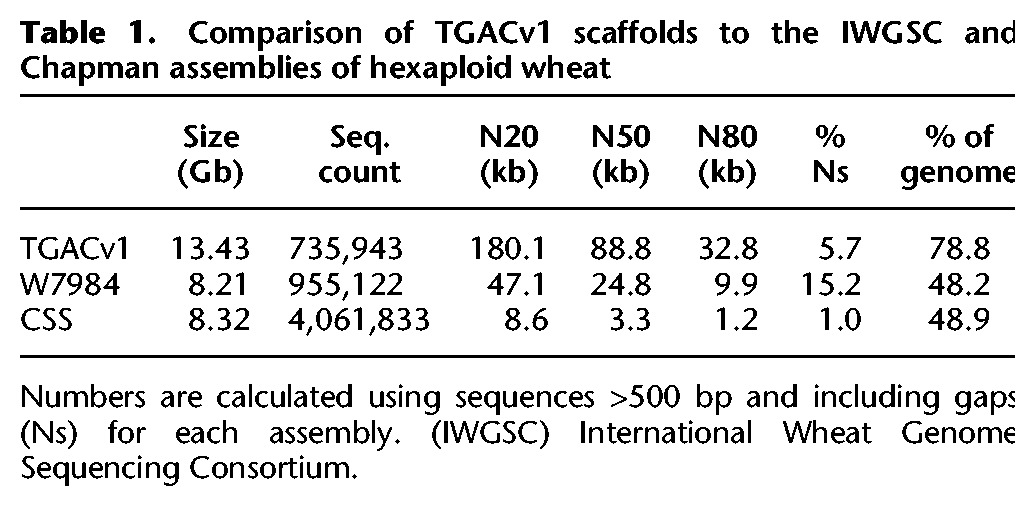
Comparison of TGACv1 scaffolds to the IWGSC and Chapman assemblies of hexaploid wheat

**Figure 1.**
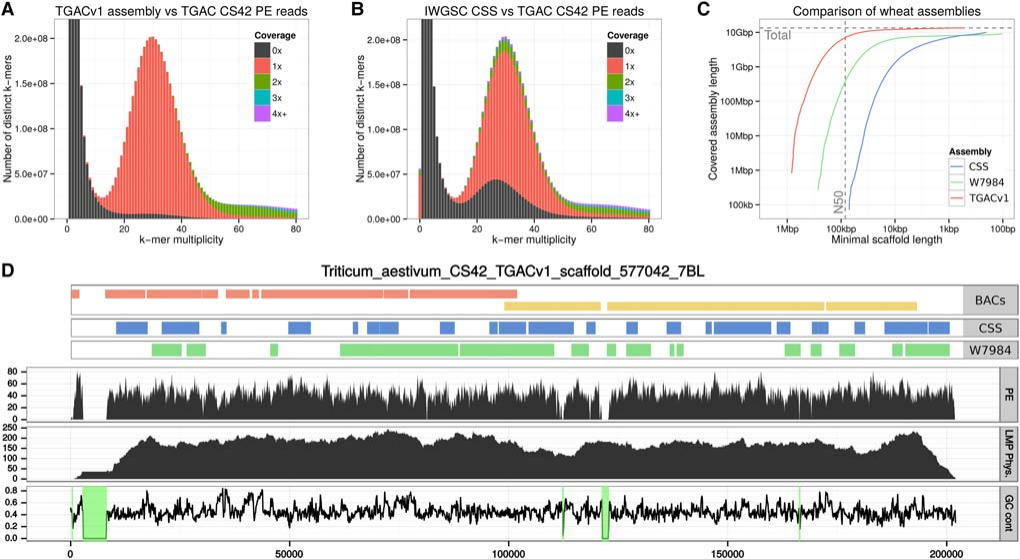
Summary of the TGACv1 wheat genome sequence assembly. (*A*,*B*) KAT spectra-cn plots comparing the PE reads to the TGACv1 scaffolds (*A*) and CSS scaffolds (*B*). Plots are colored to show how many times fixed length words (*k*-mers) from the reads appear in the assembly; frequency of occurrence (multiplicity; *x*-axis) and number of distinct *k*-mers (*y*-axis). Black represents *k*-mers missing from the assembly; red, *k*-mers that appear once in the assembly; green, twice; etc. Plots were generated using *k* = 31. The black distribution between *k*-mer multiplicity 15 and 45 in *B* represents *k*-mers that do not appear in the CSS assembly. (*C*) Comparison of scaffold lengths and total assembly sizes of the TGACv1, W7984, and CSS assemblies. (*D*) Scaffold 577042 of the TGACv1 assembly. Tracks from *top* to *bottom*: aligned BAC contigs, CSS contigs, W7984 contigs, coverage of PE reads, coverage of LMP fragments, and GC content with scaffolded gaps (N stretches) with 0% GC highlighted in green. There are two BACs (composed of seven and four contigs each), 22 CSS contigs, and 15 W7984 contigs across the single TGACv1 scaffold.

A KAT *k*-mer spectra copy number plot provides information to analyze how much and what type of *k*-mer content from reads is present in an assembly (Mapleson et al. 2017). It decomposes the *k*-mer spectrum of a read data set by the frequency in which the *k*-mers are encountered in the assembly. The plot generated from TGACv1 (Fig. 1A) showed that *k*-mers found at low frequency (less than 12), representing sequencing errors, were not found in the assembly (shown by the black distribution at *k*-mer multiplicity less than 12). Most sequence content was represented in the assembly once (shown by the main red distribution), with *k*-mers originating from the repetitive and the homoeologous regions of the genome represented at higher frequencies (more than 50). The absence of *k*-mers in the assembly that are not present in the reads indicated that the assembled contigs accurately reflected the input data. A similar analysis of the CSS assembly (Fig. 1B) identified approximately 50 million *k*-mers that were not found as sequenced content in the PCR-free paired-end data, as shown by the red bar at *k*-mer multiplicity equal to zero. This is indicative of chimeric sequences or consensus inconsistencies in the CSS assembly. The black distribution between *k*-mer multiplicity 15 and 45 shows *k*-mers from the PCR-free reads that were not present in the CSS assembly, most probably coming from the one-third of the genome not represented by the CSS assembly. The PCR-free library is expected to capture unbiased coverage of the genome, which is reflected in the increased size of the TGACv1 assembly compared with the CSS assembly. Greater amounts of duplication were observed in the single copy regions of the CSS assembly, corresponding to the purple and green areas above the main red distribution.

The content and order of genes in TGACv1 scaffolds assigned to Chromosome 3B (Supplemental Information S1; Supplemental Table S4.4) was compared to that in the Chromosome 3B BAC-based assembly (Choulet et al. 2014); 91.2% of the genes previously identified on the 3B BAC-based assembly aligned to TGACv1 scaffolds (Table 2), with no discrepancies in gene order (Supplemental Information S1; Supplemental Table S4.5). This compared with 73.9% aligned to W7984 3B scaffolds and 68.0% aligned to CSS Chromosome 3B scaffolds, demonstrating the improved representation of the TGACv1 assembly.

**Table 2.**
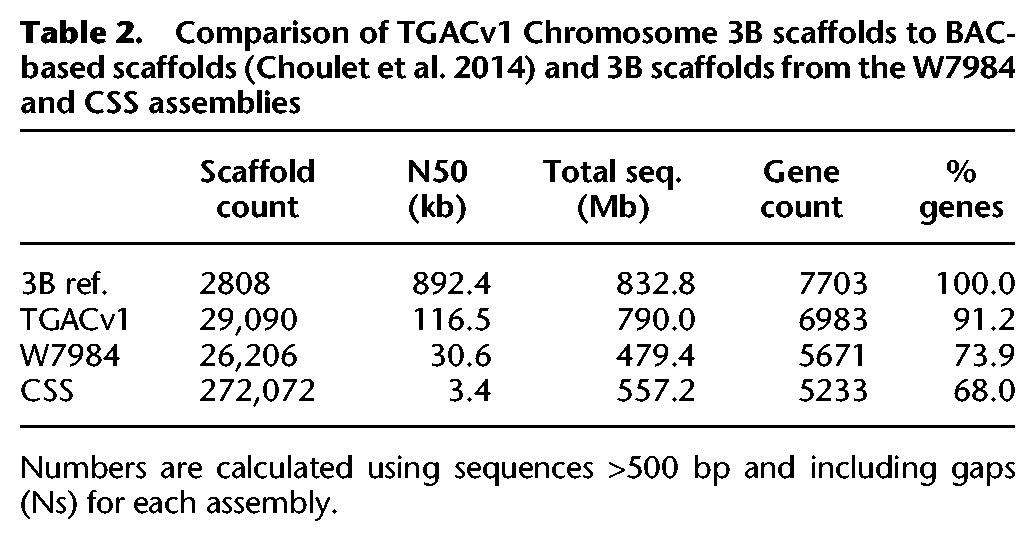
Comparison of TGACv1 Chromosome 3B scaffolds to BAC-based scaffolds (Choulet et al. 2014) and 3B scaffolds from the W7984 and CSS assemblies

**Figure 2.**
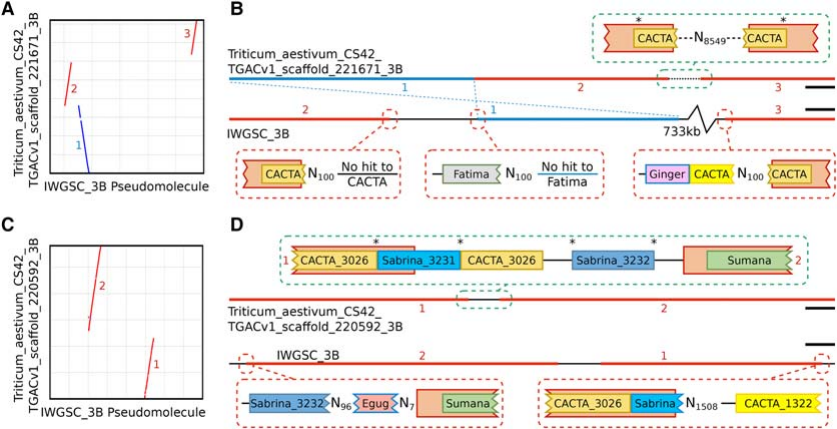
Comparative alignment of TGACv1 scaffolds with the 3B BAC-based pseudomolecule. (*A*,*C*) Dot plots between TGACv1 scaffolds and 3B show disruptions in sequence alignment, including rearrangements (red) and inversions (blue). (*B*,*D*) Graphical representation of sequence annotations in disrupted regions. Junctions in the TGACv1 scaffolds are consistent with a complete retroelement spanning the junction that includes identical TSD on either side of the retroelement (asterisks). Corresponding regions in the 3B BAC-based pseudomolecule are characterized by Ns that produce inconsistent alignment of retroelements across putative junctions. Retroelements of the same family (CACTA, Sabrina) but matching distinct members in the TREP database are indicated by different colors. Numbers adjacent to sequences correspond to regions shown in panel *A* and *C*, respectively. (*B*) Scale bars, 10 kbp; (*D*) scale bars, 30 kbp.

Alignment of TGACv1 3B scaffolds to the 3B BAC-based pseudomolecule (Fig. 2A,C) showed that they were largely in agreement. Two examples of apparent disagreement are shown in Figure 2, B and D. Scaffold_221671_3B spanned a gap of 700 kb in the 3B BAC assembly, and reoriented and removed a duplication, by identifying both ends of a CACTA element (Fig. 2B). Scaffold_220592_3B spanned 582 kb and diverged in one location (Fig. 2D) and contained a Sabrina solo-LTR with a characteristic ATCAG target site duplication (TSD). In scaffold_220592_3B, the TSD was present on either side of the Sabrina_3231 element, while in the BAC-based scaffold Sabrina homology ended in Ns. In the BAC-based assembly, only one side of the disjunction showed alignment similarity to CACTA_3026, which was found to be complete in scaffold_220592_3B and spanned the disjunction (Fig. 2D). These two examples illustrate how the TGACv1 assembly generated accurate scaffolds spanning typical complex and long tracts of repetitive DNA characterizing the wheat genome, which were misassembled in the BAC-based approach.

### Repetitive DNA composition

More than 80% of the 13.4-Gb assembly was composed of approximately 9.7 million annotated transposable element entities, of which ∼70% were retroelements (class I) and 13% DNA transposons (class II) (Supplemental Information S1; Supplemental Table S7.1). Among the class I elements, Gypsy and Copia LTR retroelements comprised the major component of the repeats, while CACTA DNA elements were highly predominant among class II DNA repeat types. No major differences in the repeat composition of the three genomes were apparent. Compared with *Brachypodium distachyon*, which has a related but much smaller genome (Vogel et al. 2010), there has been a greater than 100× increase in repeat content, driven by both class I and class II expansion. The preponderance of CACTA DNA elements in the wheat genome emerged during this massive expansion.

### Gene prediction and annotation

A total of 217,907 loci and 273,739 transcripts were identified from a combination of cross-species protein alignments, 1.5 million high-quality long Pacific Biosciences (PacBio) cDNA reads, and over 3.2 billion RNA-seq read pairs covering a range of tissues and developmental stages (Table 3; Supplemental Information S8).

**Table 3.**
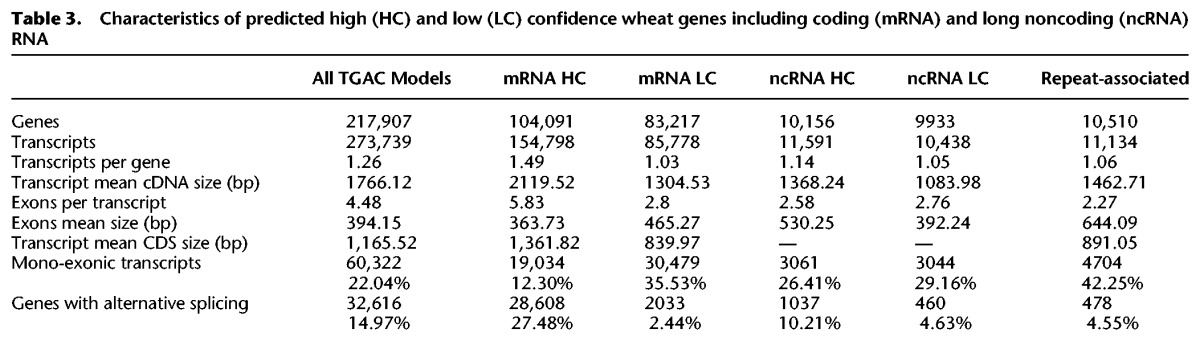
Characteristics of predicted high (HC) and low (LC) confidence wheat genes including coding (mRNA) and long noncoding (ncRNA) RNA

Loci were identified as coding, long ncRNA, or repeat associated and were classified as high (HC) or low (LC) confidence based on similarity to known plant protein sequences and supporting evidence from wheat transcripts (Supplemental Information S8.5.5). We assigned 104,091 coding genes (154,798 transcripts) as HC, of which 95,827 spanned at least 80% of the length of the best identified homolog (termed protein rank 1, P1, in the annotation) (Supplemental Fig. S8.1; Supplemental Information S8.5.1). The HC protein-coding set contained 51,851 genes confirmed by a PacBio transcript (Transcript rank 1, T1) and an additional 29,996 genes fully supported by assembled RNA-seq data (T2), providing full transcriptome support for 81,847 (78.63%) HC genes. Gene predictions were assessed by identifying 2707 single copy genes common to *B. distachyon*, *Oryza sativa*, *Sorghum bicolor*, *Setaria italica*, and *Zea mays*. A single orthologous wheat gene was identified for 2686 (99.22%) of these, with 2665 (98.45%) classified as HC and 21 (0.78%) in the LC set. A high coherence in gene length (*r* = 0.969) was found between wheat and *B. distachyon* proteins (Supplemental Fig. S8.2). These findings show that the HC gene set is robust and establishes a lower bound estimate for the total number of protein-coding genes in wheat. An additional 103,660 loci were defined as LC (i.e., gene models with all their transcripts either having <60% protein coverage or lacking wheat transcript support). These include bona fide genes that were fragmented due to breaks in the current assembly, wheat-specific genes, and genes without transcriptome support (Supplemental Table S8.8).

We also identified 10,156 HC noncoding genes with little similarity in protein databases and low protein-coding potential. The majority of these genes are located in intergenic regions (8854, or 87.18%), while most of the remaining 1302 are anti-sense to coding genes (1082, or 10.65%) (see Supplemental Information, section 8.5.8); 5413 of wheat noncoding genes (53.30%) were detected in at least one of the two sequenced wheat diploid progenitor species *Triticum urartu* and *Aegilops tauschii* (at least 90% coverage and 90% identity) (see Supplemental Information S8.5.8).

To obtain additional support for gene predictions, a proteome map was constructed from 27 wheat tissues (Supplemental Information S9). This identified 2,106,323 significant peptide spectrum matches corresponding to 102,379 distinct peptides. Of these, 96.20% matched HC genes, while 13.29% were assigned to LC genes. For 56,391 genes (43,431 HC, 12,960 LC), we were able to identify at least one peptide confirming the predicted coding sequence. Due to the hexaploid nature of wheat, only 22.1% of the peptides could be assigned to a single gene. Applying progressively stricter filters, by requiring at least two or five peptides, confirmed the protein sequence of 30,607 and 17,316 HC genes, respectively; 10,819 genes met the criteria of having support from multiple peptides with at least one uniquely identifying peptide and were considered as unambiguously corroborated by proteomic data. Among the LC genes, only 368 were identified by two or more peptides that did not match any HC gene, further supporting confidence assignments. Among these, 343 were classified as LC due to having <60% the length of the identified homolog, while the remaining 25 genes were classified as LC due to either repeat association or lack of wheat transcript support.

**Figure 3.**
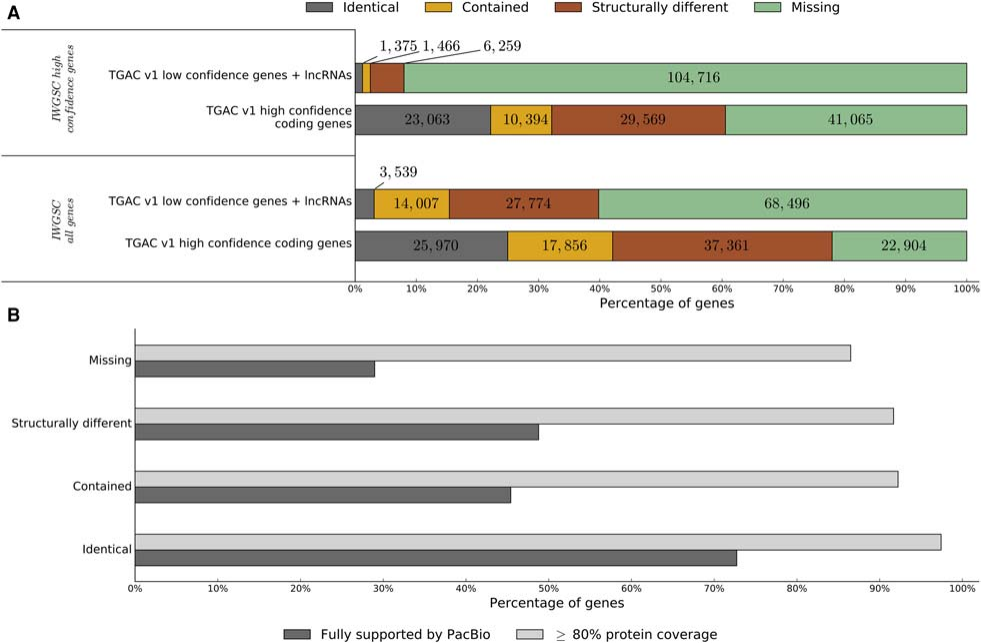
Comparison between IWGSC annotation and TGACv1 high (HC) and low confidence (LC) genes. IWGSC genes were aligned to the TGACv1 assembly (gmap, ≥90% coverage, ≥95% identity) and classified based on overlap with TGACv1 genes. (*A*) Identical indicates shared exon–intron structure; contained, exactly contained within the TGACv1 gene; structurally different, alternative exon–intron structure; and missing, no overlap with IWGSC. (*B*) Bar plot showing proportion of HC TGACv1 protein-coding genes supported by protein similarity or PacBio data. Genes are classified based on overlap with the full set of IWGSC genes.

We compared the TGACv1, CSS (The International Wheat Genome Sequencing Consortium 2014), and Chromosome 3B (Choulet et al. 2014) gene models. Of the 100,344 HC genes in the International Wheat Genome Sequencing Consortium (IWGSC) annotation (PGSB/MIPS version 2.2 and INRA version 1.0 from Ensembl release 29), we were able to transfer 97,072 (97%) to the TGACv1 assembly with stringent alignment parameters (at least 90% coverage and 95% identity). Fewer (72%) of the IWGSC (The International Wheat Genome Sequencing Consortium 2014) LC, unsupported, repeat associated, and noncoding loci could be aligned (at least 90% coverage and 95% identity), likely reflecting differences between the assemblies of repeat rich and difficult to assemble regions. Of the TGACv1 HC genes, 61% overlapped with an aligned IWGSC HC gene and 78% to the full IWGSC gene set (Supplemental Information S8.5.7). Less agreement was found between TGACv1 LC and ncRNA genes and the IWGSC annotation, with only 8% overlapping IWGSC HC loci and 40% overlapping the full IWGSC gene set (Fig. 3A). Of the 22,904 (22%) HC TGACv1 genes not overlapping a transferred IWGSC gene, 19,810 (86%) had cross-species protein similarity support with 6665 (29%) fully supported by a PacBio transcript (Fig. 3B). We identified 13,609 TGACv1 genes that were overlapped by transcripts originating from two or more IWGSC genes in our annotation, indicating that they were likely fragmented in the CSS assembly. In 8175 of these cases (60%), we were able to find a PacBio read fully supporting our gene model. These differences reflect improvements in contiguity, a more comprehensive representation of the wheat gene space in our assembly, and improved transcriptome support for annotation.

### Alternative splicing

Alternative splicing is an important mRNA processing step that increases transcriptome plasticity and proteome diversity (Staiger and Brown 2013). The TGACv1 annotation includes high-quality alternative splicing variants identified from PacBio transcriptome reads. To provide a more comprehensive representation of alternative splicing, we subsequently integrated transcript assemblies generated from six strand-specific Illumina libraries (Supplemental Information S8.6; Supplemental Table S8.1). This added a further 121,997 transcripts, increasing the number of genes with splice variants from 15% in the TGACv1 annotation to 31% in the supplemented set of transcripts (i.e., incorporating Illumina RNA-seq assemblies), as well as increasing the average number of transcripts per gene from 1.26 to 1.88. When considering only HC genes, the number of alternatively spliced genes was increased from 27.48% to 48.80% (2.36 transcripts per gene), similar to that observed in a wide range of plant species (Zhang et al. 2015).

Intron retention (IR) was the prevalent alternative splicing event in wheat (34%) followed by alternative 3′ splice sites (A3SS; 27%), exon skipping (ES; 20%), alternative 5′ splice sites (A5SS; 19%), and mutually exclusive exons (MXE; 0.04%). This was similar to previous analyses of Chromosome 3B (Pingault et al. 2015), and IR is also predominant in barley (Panahi et al. 2015). Alternative splicing coupled to nonsense mediated decay (NMD) regulates gene expression (Lykke-Andersen and Jensen 2015). We found 22% of all transcripts (17% of all genes) and 29% of multiexonic HC protein-coding transcripts (33% genes) may be potential targets for NMD. IR was the most common splicing event leading to NMD sensitivity, with 40% of IR transcripts identified as potential NMD targets (34% ES, 38% A5SS, 34% A3SS, 26% MXE). This suggests a potentially substantial role for alternative splicing/NMD in regulating gene expression in wheat.

### Gene families

HC and LC gene families were analyzed separately using OrthoMCL version 2.0 (Li et al. 2003; Supplemental Figs. S10.1, S10.2). Splice variants were removed from the HC gene data set, keeping the representative transcript for each gene model (see Supplemental Information S8.5.6, S10.1), and data sets were filtered for premature termination codons and incompatible reading frames. For the HC gene set, a total of 87,519 coding sequences were clustered into 25,132 gene families. The vast majority of HC gene families contained members from the A, B, and D genomes, consistent with the relatively recent common ancestry of the A and B genomes and the proposed hybrid origin of the D genome from ancestral A and B genomes (Marcussen et al. 2014). Subsets of gene families and singleton genes (those not clustered into any family) were classified to identify (1) genes and families that are A, B, or D genome specific; (2) gene families with expanded numbers in one genome; and (3) wheat gene families that are expanded relative to other species. These gene sets were analyzed for overrepresented Gene Ontology (GO) terms, shown in Supplemental File S2. Gene families that were significantly expanded in wheat compared with *Arabidopsis*, rice, sorghum, and *Brachypodium* include those encoding proteins involved in chromosome maintenance and reproductive processes, as well as protein and macromolecule modification and protein metabolism processes. The D genome has expanded gene families encoding phosphorylation, phosphate metabolism, and macromolecule modification activities, while the B genome has expanded gene families encoding components of chromosome organization, DNA integration and conformation/unwinding, and telomere maintenance. The B genome is derived from the *Sitopsis* section of the Triticeae, which has contributed genomes to many polyploid Triticeae species (Riley et al. 1961), suggesting B genomes may have contributed gene functions for establishing and maintaining polyploidy in the Triticeae. This is supported by the location of the major chromosome pairing *Ph1* locus on Chromosome 5B (Griffiths et al. 2006).

### Genome organization

A corrected version of the POPSEQ genetic map (Chapman et al. 2015) was used to order TGACv1 scaffolds along chromosomes (Supplemental information S5). This uniquely assigned 128,906 (17.5 %) of the 735,943 TGACv1 scaffolds to 1051 of 1187 genetic bins (class 1) (Supplemental Information S5) to form the final TGACv1 map. The total length of these scaffolds is 8,551,191,083 bp, representing 63.68% of the TGACv1 assembly and 50.52% of the 17-Gbp wheat genome. A further 13,019 (1.77%) scaffolds were ambiguously assigned to different cM positions on the same chromosome (class 2), 489 (0.07%) scaffolds were assigned to homoeologous chromosomes (class 3), and 3320 (0.45%) scaffolds had matching markers with conflicting bin assignment (class 4).

The TGACv1 map also assigns unique chromosomal positions to 3927 (3.05%) scaffolds that were not previously assigned to a chromosome arm (class 5). The CSS-based chromosome arm assignments of 380 (0.295%) class1 scaffolds and 11 (0.08%) class 2 scaffolds disagree with the map-based chromosome assignments (classes 6, 7). A list of scaffold classifications can be found in Supplemental Information S6.

The TGACv1 map encompasses 38,958 of the 53,792 scaffolds containing at least one annotated HC protein-coding gene (72.42%), comprising gene sequences of 307,085,968 bp (73.28% of total predicted gene sequence space). In total, we were able to assign genetic bins to 75,623 (72.65%) of the HC genes.

**Figure 4.**
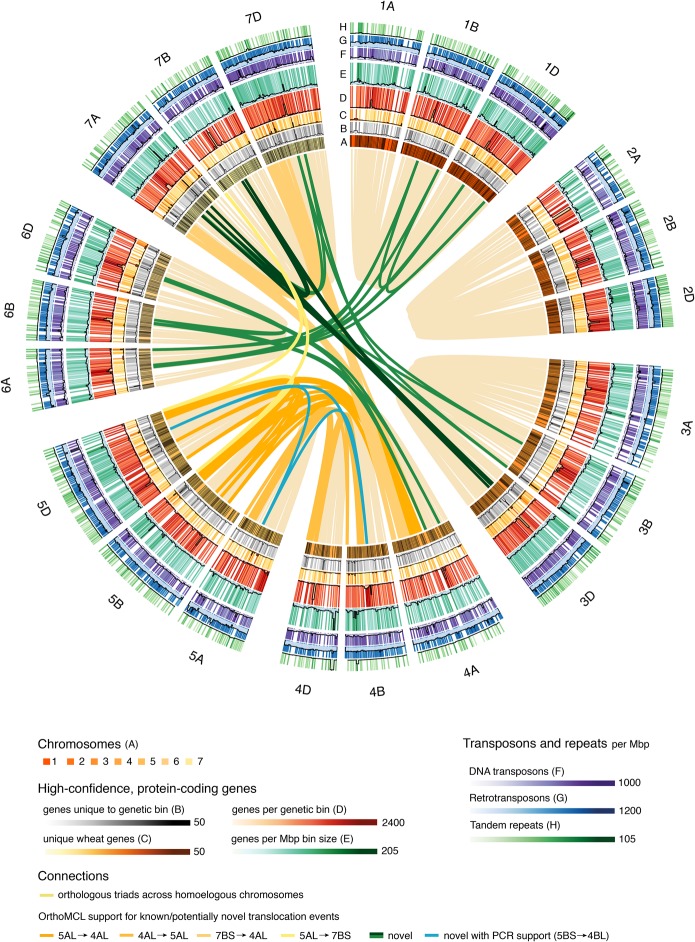
Circular representation of the TGACv1 CS42 assembly. Chromosomes, genetic bins, and genomic features are visualized on the *outer* rings (*A*–*H*) and interchromosomal links identify known and potentially novel translocation events. (*A*) The seven chromosome groups of the *A*, *B*, and *D* genomes, scaled by number of genetic bins (black bands). (*B*–*H*) Combined heatmap/histogram representations of genomic features per genetic bin. With the exception of *D*, all counts are normalized by the size of the genetic bin in Mbp, calculated as the total size of all scaffolds assigned to the bin. (*B*) Distribution of unique genes, i.e., genes that did not have orthologs in a genome-wide OrthoMCL screen. (*C*) Distribution of wheat-specific genes. (*D*,*E*) Number of HC protein-coding genes. (*F*) Distribution of DTC, DTM, and DTH DNA transposons (Supplemental Information S1; Supplemental Table S7.1). (*G*) Distribution of RLX, RLC, RLG, RXX, and RIX retrotransposons. (*H*) Distribution of tandem duplications. Light yellow links connect homoeologous OrthoMCL triads. Dark yellow-colored links connect genetic bins harboring OrthoMCL outlier triads (Supplemental Information S1, section S6) that identify known translocation events. Dark green links connect genetic bins harboring at least three OrthoMCL outlier triads that may support novel translocation events. The cyan link shows a novel PCR-validated translocation event between Chromosomes 5BS-4BL.

Chromosomal locations of related genes were identified by anchoring to the TGACv1 map and are displayed in Figure 4. Analysis of OrthoMCL outlier triads (Supplemental Information S1, sections S6, S10) provided genomic support for known ancestral reciprocal translocations between chromosome arms 4AL and 5AL, a combination of pericentromeric inversions between chromosome arms 4AL and 5AL, and a reciprocal exchange between chromosome arms 4AL and 7BS (Devos et al. 1995). Several putative novel chromosomal translocations were also identified (Fig. 4; Supplemental File S3). As these may have originated in the parental lines used in the POPSEQ map rather than in CS42, nine genes in the predicted translocations (six previously known and three novel) were tested using PCR assays on Chinese Spring chromosomal deletion stocks (Sears et al. 1966). Three known translocation events—4AL-5AL and 7BS-4AL (Devos et al. 1995) and 5AL-7BS (Ma et al. 2013)—and one previously unidentified translocation, 5BS-4BL, were validated by PCR assays.

### Gene expression

To explore global gene expression patterns, we mapped multiple wheat RNA-seq data sets to the TGACv1 transcriptome (Supplemental Information S1; Supplemental Table S11.1). Seventy-five percent of RNA-seq reads mapped to the TGACv1 transcriptome (Supplemental Information S1; Supplemental Table S11.1), and 78% of the HC protein-coding transcripts were expressed above the background level of 2 tpm (Wagner et al. 2013). Interestingly, 23% of the LC genes were also expressed above 2 tpm. Expression levels of genes across chromosomes were similar, with the exception of 19 genetic bins that had increased expression (defined as “hotspots” with a median expression level >20 tpm, containing on average 5 genes) across the six tissues examined (Supplemental Information; Supplemental Fig. S11.1). Hotspots tended to be enriched for genes encoding components of the cytoskeleton, ribosome biogenesis, and nucleosome assembly that were expressed at high levels in all tissues. Other notable hotspots were enriched in genes of photosystem I formation in leaf tissues, and nutrient reservoir activity in seed tissues.

**Figure 5.**
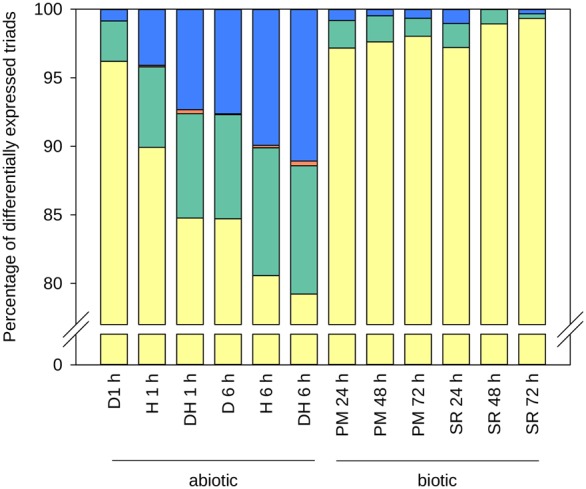
Response of differentially expressed (DE) triads to stress treatments according to the number and pattern of DE homoeologs. Triads were classified as having one homoeolog DE (yellow), two homoeologs DE with same direction of change (green), three homoeologs DE with same direction of change (orange), or opposite direction of change between DE homoeologs (blue). The stresses applied were drought (D), heat (H), drought and heat combined (DH), powdery mildew (PM), and stripe rust (SR), with the duration of stress application indicated in hours (h).

The more complete and accurate annotation provided an opportunity to analyze patterns of transcript levels in homoeologous triads. Transcript levels of 9642 triads were analyzed in response to biotic and abiotic stress using publicly available RNA-seq data sets, selected as they all used 7-d-old seedlings, were replicated, and assessed dynamic transcriptional responses to standardized treatments (Supplemental Information S1; Supplemental Table S11.2). Across treatments, 26% (2424 of 9159) of expressed triads showed higher expression in one or two genomes in at least one stress condition (rather than balanced expression of three genomes) (see Supplemental Information S11.5). Abiotic stress led to more differentially regulated transcripts, compared with biotic stress responses, across all three genomes. To assess the conservation of this stress response between homoeologs, we classified each homoeolog as either up-regulated (greater than twofold change, UP), down-regulated (less than 0.5-fold change, DOWN), or flat (between 0.5-fold to twofold change). We then assessed whether the individual homoeolog response to stress compared with control conditions was consistent (Supplemental Information S1; Supplemental Table S11.3). Eighty percent (±5.1% SE) of triads were not differentially expressed in response to the stress treatments and were excluded from further analysis. The most frequent pattern of differential triad expression was a single homoeolog UP or DOWN, with the other two remaining flat (79%–99% across conditions) (Fig. 5). Triads in which either all homoeologs were expressed in the same pattern (“3 UP” or “3 DOWN”) were rare, as were triads in which homoeologs were expressed in opposite directions. This is consistent with Liu et al. (2015), who identified between 13% and 41% of homoeolog triads in which homoeologs did not respond to the same degree in response to stress conditions.

The genomic context of differences in homoeolog expression was explored in genomic regions containing at least five HC genes in syntenic order on all three genomes, of which at least one homoeolog was expressed over background levels in root, shoot, and endosperm tissue at 10 and 20 d post anthesis (DPA; DRP000768 and ERP004505) (Supplemental Information S1; Supplemental Table S11.1; Pfeifer et al. 2014). Of the four blocks meeting these criteria, one showed equal expression of all 15 homoeologs in at least one of the tissues, while the other three blocks showed unbalanced expression of at least one homoeolog (Supplemental Information S1; Supplemental Fig. S11.2). All blocks exhibited major structural and promoter sequence differences, as well as variant transcription start sites (Supplemental Information S1; Supplemental Fig. S11.3). These multiple types of genomic differences all have the potential to contribute to unbalanced expression. To facilitate further expression studies the expression atlas at http://www.wheat-expression.com has been updated with the TGACv1 annotation and expression data from 424 RNA samples (Borrill et al. 2015).

### Gene families of agronomic interest

#### Wheat disease–resistance genes

Plant disease–resistance (*R-*) genes termed nucleotide binding site–leucine rich receptors (NBS-LRRs) (Dodds and Rathjen 2010) are challenging to assemble as they are often organized in multigenic clusters with many tandem duplications and rapid pseudogenization. The TGACv1 assembly contains 2595 NBS-containing genes (Table 4) of which 1185 are NBS-LRR genes. Among these, 98% have complete transcripts compared with only 2% in the CSS assembly. We also used NLR-parser (Steuernagel et al. 2015) to predict the coiled-coil (CC-) NBS-LRR subclass of *R*-genes. We identified 859 complete CC-NBS-LRR genes supported by specific MEME motifs (Jupe et al. 2012) compared with 225 in the CSS assembly (Table 4). The total of 1185 wheat NBS-NLRs was consistent with that found in diploid wheat progenitors (402 NLRs in *T. urartu*) and diploid relatives (438 in *O. sativa*) (Sarris et al. 2016). Nearly 90% of CS42 *R*-genes were unambiguously assigned to chromosome arms, and 57% (674/1185) were anchored to the TGACv1 map. The number of *R*-genes per scaffold ranged from one to 31, compared with only two to three *R*-gene per scaffold in the CSS wheat assembly (The International Wheat Genome Sequencing Consortium 2014). This finding is corroborated by BAC sequence assemblies (Supplemental Information S1; Supplemental Fig. S12.1).

**Table 4.**
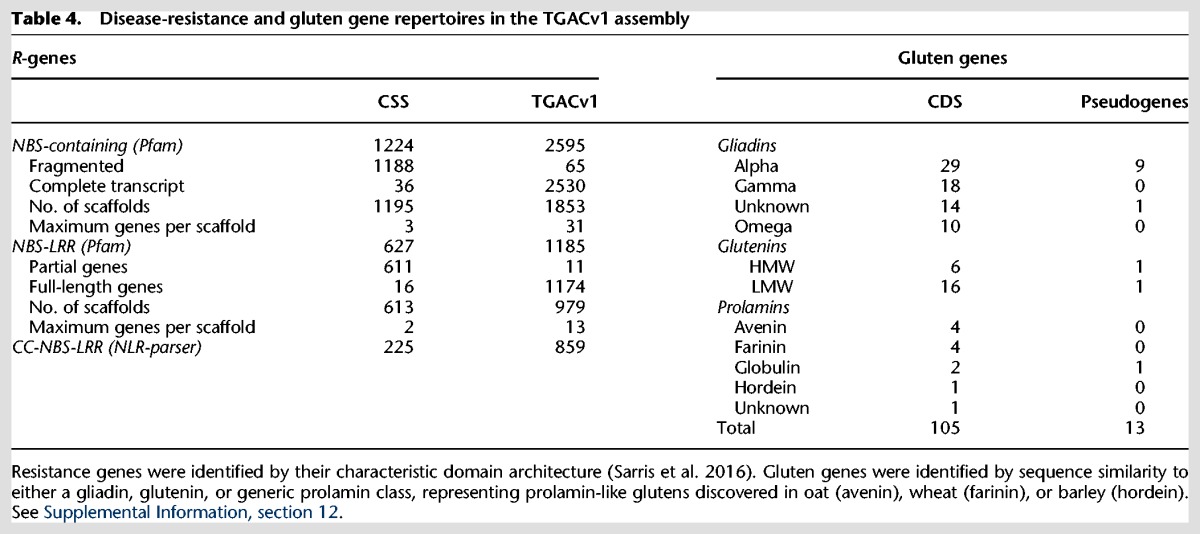
Disease-resistance and gluten gene repertoires in the TGACv1 assembly

#### Gluten genes

Glutens form the major group of grain storage proteins, accounting for 10%–15% of grain dry weight, and confer viscoelastic properties essential for bread-making (Shewry et al. 1995). Gluten genes encode proteins rich in glutamines and prolines that form low-complexity sequences composed of PxQ motifs, and occur in tandem repeats in highly complex loci that have posed significant challenges for their assembly and annotation. We characterized the gluten genes in the TGACv1 assembly and showed that most of the known genes were fully assembled. Gluten loci, while still fragmented, exhibit much greater contiguity than in the CSS assembly (The International Wheat Genome Sequencing Consortium 2014) with up to six genes per scaffold (Supplemental Information S1; Supplemental Fig. S12.2). We identified all assembly regions with nucleotide similarity to publicly available gluten sequences, adding an additional 33 gluten genes to the annotation and manually correcting 21 gene models. In total, we identified 105 full-length or partial gluten genes and 13 pseudogenes in the TGACv1 assembly (Table 4; Supplemental information S1, section S12.2).

#### The gibberellin biosynthetic and signaling pathway

**Figure 6.**
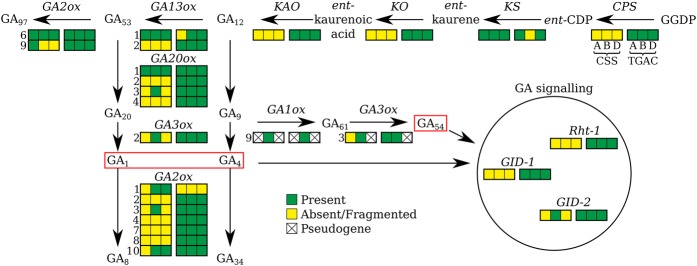
Genes encoding the gibberellin (GA) biosynthetic and signaling pathway in bread wheat. The GA biosynthesis, inactivation, and signal transduction pathway, illustrating the representation of the gene sequences in CSS and TGACv1 assemblies. If more than one paralog is known for a gene, its number according to the classification by Pearce et al. (2015) is indicated on the *left* of the box. Bioactive GAs are boxed in red.

Mutations in the gibberellin (GA) biosynthetic and signal transduction pathways have been exploited in wheat, where gain-of-function mutations in the GA signaling protein Rht-1 confer GA insensitivity and a range of dwarfing effects. Most modern wheat cultivars carry semi-dominant *Rht-1* alleles (Phillips 2016), but these alleles also confer negative pleiotropic effects, including reduced male fertility and grain size. Hence, there is considerable interest in developing alternative dwarfing alleles based on GA-biosynthetic genes such as *GA20ox2*. A prerequisite for this is access to a complete set of genes encoding the biosynthetic pathway. Figure 6 shows that the TGACv1 assembly contains full-length sequences for 67 of the expected 72 GA pathway genes, in contrast to only 23 genes in the CSS assembly (The International Wheat Genome Sequencing Consortium 2014). Two paralogs of *GA20ox3* on Chromosome 3D are separated by 460 kb, and *GA1ox-B1* and *GA3ox-B3* are separated by 3.2 kb, suggesting common ancestry of these two enzymes with different catalytic activities (Pearce et al. 2015).

## Discussion

Access to a complete and robust wheat genome assembly is essential for the continued improvement of wheat, a staple crop of global significance with 728 M tonnes produced in 2014 (http://fenix.fao.org/faostat/beta/en/#home). The capacity to assemble and annotate wheat genomes accurately, rapidly, and cost-effectively addresses key social, economic, and academic priorities by facilitating trait analyses, by exploiting diverse germplasm resources, and by accelerating plant breeding. However, polyploidy and the extensive repeat regions in wheat have limited the completeness of previous assembly efforts (Brenchley et al. 2012; The International Wheat Genome Sequencing Consortium 2014; Chapman et al. 2015), reducing their utility.

Here we report a much more complete wheat genome assembly, representing ∼80% of the 17-Gb genome in large scaffolds. We combined high-quality PCR-free libraries and precisely size-selected LMP libraries (Heavens et al. 2015) with the w2rap assembly software (Clavijo et al. 2017) to generate contiguous and complete assemblies from relatively low (about 33×) Illumina PE read coverage and LMP libraries. The contiguity of the TGACv1 assembly allowed us to create a greatly improved gene annotation supported by extensive transcriptome data. Over 78% of the 104,091 HC protein-coding genes are fully supported by RNA-seq data. These improvements identified 22,904 genes that were absent from previous wheat gene sets (The International Wheat Genome Sequencing Consortium 2014; Choulet et al. 2014), almost all of which have a homolog in other species (Fig. 3B). The robustness of the annotation is further supported by the use of high-quality PacBio data and agreement with proteomic data, with 42% of the HC gene models supported by sequenced peptides. This new wheat gene set provides an improved foundation for wheat research. Finally, incorporation of strand-specific Illumina RNA-seq libraries into the annotation showed that nearly half of the HC genes were alternatively spliced, in line with observations in many other plants (Zhang et al. 2015).

A well-defined gene set in large sequence scaffolds is an essential foundation for trait analyses in wheat. We identified the complement of disease-resistance genes, gluten protein genes that confer nutritional and bread-making quality of wheat grains, and the set of GA biosynthetic and signal transduction genes that are important determinants of crop height and yield. An accurate gene set is also essential for understanding expression of gene families in complex allopolyploid genomes. We observed that 20% of homoeologous triads showed differential expression in seedling leaves subject to biotic and abiotic stress conditions. This is consistent with coexpression analyses in developing grains (Pfeifer et al. 2014), where most differentially expressed genes were single homoeologs that were up/down-regulated. Taken together, these results identify widespread subfunctionalization of homoeologous genes due to differential regulation. The new assembly and annotation will enable the identification of multiple sequence differences in promoters, transcription start sites, gene splicing, and other features among strict homoeologs, providing a foundation for systematic analyses of the causes of these differences.

Generating complete and accurate wheat genome assemblies is essential for capturing the full range of genetic variation in wheat genomes. By identifying this variation, genomics will directly facilitate trait analyses and accelerate plant breeding. Our rapid, accurate, and cost-effective assembly approach is suitable for assembling multiple wheat and other Triticeae genomes in robust and comparable ways, using relatively inexpensive sequencing technologies based on PCR-free libraries and open-source software. We anticipate that researchers with access to suitable computational infrastructure will use the approaches described here to sequence multiple wheat varieties, including elite varieties, unimproved landraces, and progenitor species. These assemblies will reveal a wide spectrum of genetic variation, including large-scale structural changes such as translocations and chromosome additions that are known to play a major role in the adaptation of the wheat crop to different growing environments. By adopting this pan-genomics approach, we will enrich our understanding of complex genome evolution and the plasticity of genome regulation and empower new approaches to wheat improvement.

## Methods

### DNA library preparation and sequencing

A full description of the DNA preparation and sequencing methods is in Supplemental Information. PCR-free PE libraries were sequenced using 2× 250-bp reads on HiSeq2500 platforms for contig generation. TALL libraries and Nextera LMP libraries (Heavens et al. 2015) were used for scaffolding. Insert size distributions (Supplemental Information S1; Supplemental Figs. S4.1–S4.3) were checked by mapping to the CS42 Chromosome 3B pseudomolecule (Choulet et al. 2014) using the DRAGEN coprocessor (http://www.edicogenome.com/dragen/).

### Genome assembly

Assembly was performed using the Wheat/Whole-Genome Robust Assembly Pipeline, w2rap (Clavijo et al. 2017). It combines the w2rap-contigger, based on DISCOVAR de novo (Weisenfeld et al. 2014), an LMP preparation approach based on FLASH (Magoc and Salzberg 2011) and Nextclip (Leggett et al. 2014), and scaffolding with SOAPdenovo2 (Luo et al. 2012). The w2rap-contigger takes advantage of DISCOVAR (Weisenfeld et al. 2014; Love et al. 2016) algorithms to preserve sequence variation during assembly but has been further developed to enable processing of much larger data volumes and complex genomic repeats. The paired-end read data set was assembled into contigs on a SGI UV200 machine with 7TB of shared RAM. The contig assembly took 38 d using 64 cpus, with the default settings of the w2rap-contigger from https://github.com/bioinfologics/w2rap-contigger/releases/tag/CS42_TGACv1. Newer versions of w2rap can achieve similar results in half the time or less, using close to half the memory. Scaffolding with the LMP data took a total of 10 d and was executed on the same hardware but used 128 cpus and <1 TB of RAM. Contigs were scaffolded using the PE, LMP, and TALL reads and the SOAPdenovo2 (Luo et al. 2012) prepare→map→scaffold pipeline, run at *k* = 71. Contigs and scaffolds were quality controlled using KAT spectra-cn plots (Mapleson et al. 2017) to assess motif representation.

### Gene annotation

A high-quality gene set for wheat was generated using a custom pipeline integrating wheat-specific transcriptomic data, protein similarity, and evidence-guided gene predictions generated with AUGUSTUS (Stanke and Morgenstern 2005). Full methods are in Supplemental Information S8. RNA-seq reads (ERP004714, ERP004505, and 250-bp PE strand-specific reads from six different tissues) were assembled using four alternative assembly methods (Trapnell et al. 2010; Haas et al. 2013; Pertea et al. 2015; Song et al. 2016) and integrated with PacBio transcripts into a coherent and nonredundant set of models using Mikado (https://github.com/lucventurini/mikado). PacBio reads were then classified according to protein similarity and a subset of high-quality (e.g., full length, canonical splicing, nonredundant) transcripts used to train an AUGUSTUS wheat-specific gene prediction model. AUGUSTUS was then used to generate a first draft of the genome annotation, using as input Mikado-filtered transcript models, reliable junctions identified with Portcullis (https://github.com/maplesond/portcullis), and peptide alignments of proteins from five close wheat relatives (*B. distachyon*, maize, rice, *S. bicolor*, and *S. italica*). This draft annotation was refined by correcting probable gene fusions, missing loci and alternative splice variants. The annotation was functionally annotated, and all loci were assigned a confidence rank based on their similarity to known proteins and their agreement with transcriptome data.

## Data access

All data generated in this study have been submitted to the European Nucleotide Archive (ENA; http://www.ebi.ac.uk/ena) under accession numbers PRJEB15378, PRJEB15378 (PE and LMP reads used for genome assembly and scaffolding), PRJEB11773 (genome assembly), and PRJEB15048 (Illumina and PacBio reads used for genome annotation). The assembly and annotation are available in Ensembl Plants (release 32; Ensembl Plants, http://plants.ensembl.org/Triticum_aestivum/Info/Index) and from the Earlham Institute Open Data site (EI; http://opendata.earlham.ac.uk/Triticum_aestivum/TGAC/v1/). BLAST services for these data sets are available via Grassroots Genomics (Grassroots; https:// wheatis.tgac.ac.uk/grassroots-portal/blast).

## Supplementary Material

Supplemental Material

## References

[CLAVIJOGR217117C1] The 1000 Genomes Project Consortium. 2010 A map of human genome variation from population-scale sequencing. Nature 467: 1061–1073. 20981092 10.1038/nature09534 PMC3042601

[CLAVIJOGR217117C2] Abdel-Ghany SE, Hamilton M, Jacobi JL, Ngam P, Devitt N, Schilkey F, Ben-Hur A, Reddy ASN. 2016 A survey of the sorghum transcriptome using single-molecule long reads. Nat Commun 7: 11706. 27339290 10.1038/ncomms11706 PMC4931028

[CLAVIJOGR217117C3] Aird D, Ross MG, Chen W-S, Danielsson M, Fennell T, Russ C, Jaffe DB, Nusbaum C, Gnirke A. 2011 Analyzing and minimizing PCR amplification bias in Illumina sequencing libraries. Genome Biol 12: R18. 21338519 10.1186/gb-2011-12-2-r18 PMC3188800

[CLAVIJOGR217117C4] Berthelot C, Brunet F, Chalopin D, Juanchich A, Bernard M, Noël B, Bento P, Da Silva C, Labadie K, Alberti A, 2014 The rainbow trout genome provides novel insights into evolution after whole-genome duplication in vertebrates. Nat Commun 5: 3657. 24755649 10.1038/ncomms4657 PMC4071752

[CLAVIJOGR217117C5] Bickhart DM, Rosen BD, Koren S, Sayre BL, Hastie AR, Chan S, Lee J, Lam ET, Liachko I, Sullivan ST, 2017 Single-molecule sequencing and chromatin conformation capture enable de novo reference assembly of the domestic goat genome. Nat Genet (in press). 10.1038/ng.3802. PMC5909822 28263316

[CLAVIJOGR217117C6] Bishara A, Liu Y, Weng Z, Kashef-Haghighi D, Newburger DE, West R, Sidow A, Batzoglou S. 2015 Read clouds uncover variation in complex regions of the human genome. Genome Res 25: 1570–1580. 26286554 10.1101/gr.191189.115 PMC4579342

[CLAVIJOGR217117C7] Blanc G, Wolfe KH. 2004 Widespread paleopolyploidy in model plant species inferred from age distributions of duplicate genes. Society 16: 1667–1678. 10.1105/tpc.021345 PMC514152 15208399

[CLAVIJOGR217117C8] Borrill P, Adamski N, Uauy C. 2015 Genomics as the key to unlocking the polyploid potential of wheat. New Phytologist 208: 1008–1022. 26108556 10.1111/nph.13533

[CLAVIJOGR217117C9] Brenchley R, Spannagl M, Pfeifer M, Barker GLA, D'Amore R, Allen AM, McKenzie N, Kramer M, Kerhornou A, Bolser D, 2012 Analysis of the bread wheat genome using whole-genome shotgun sequencing. Nature 491: 705–710. 23192148 10.1038/nature11650 PMC3510651

[CLAVIJOGR217117C10] Chaisson MJP, Wilson RK, Eichler EE. 2015 Genetic variation and the de novo assembly of human genomes. Nat Rev Genet 16: 627–640. 26442640 10.1038/nrg3933 PMC4745987

[CLAVIJOGR217117C11] Chapman JA, Ho I, Sunkara S, Luo S, Schroth GP, Rokhsar DS. 2011 Meraculous: de novo genome assembly with short paired-end reads. PLoS One 6: e23501. 21876754 10.1371/journal.pone.0023501 PMC3158087

[CLAVIJOGR217117C12] Chapman JA, Mascher M, Buluç A, Barry K, Georganas E, Session A, Strnadova V, Jenkins J, Sehgal S, Oliker L, 2015 A whole-genome shotgun approach for assembling and anchoring the hexaploid bread wheat genome. Genome Biol 16: 26. 25637298 10.1186/s13059-015-0582-8 PMC4373400

[CLAVIJOGR217117C13] Choulet F, Alberti A, Theil S, Glover N, Barbe V, Daron J, Pingault L, Sourdille P, Couloux A, Paux E, 2014 Structural and functional partitioning of bread wheat chromosome 3B. Science 345: 1249721. 25035497 10.1126/science.1249721

[CLAVIJOGR217117C14] Clavijo B, Garcia Accinelli G, Wright J, Heavens D, Barr K, Yanes L, Di Palma F. 2017 W2RAP: a pipeline for high quality, robust assemblies of large complex genomes from short read data. bioRxiv 2017: 110999.

[CLAVIJOGR217117C15] Devos KM, Dubcovsky J, Dvorˇák J, Chinoy CN, Gale MD. 1995 Structural evolution of wheat chromosomes 4A, 5A, and 7B and its impact on recombination. Theor Appl Genet 91: 282–288. 24169776 10.1007/BF00220890

[CLAVIJOGR217117C16] Dodds PN, Rathjen JP. 2010 Plant immunity: towards an integrated view of plant–pathogen interactions. Nat Rev Genet 11: 539–548. 20585331 10.1038/nrg2812

[CLAVIJOGR217117C17] Gan X, Stegle O, Behr J, Steffen JG, Drewe P, Hildebrand KL, Lyngsoe R, Schultheiss SJ, Osborne EJ, Sreedharan VT, 2011 Multiple reference genomes and transcriptomes for *Arabidopsis thaliana*. Nature 477: 419–423. 21874022 10.1038/nature10414 PMC4856438

[CLAVIJOGR217117C18] Gnerre S, MacCallum I, Przybylski D, Ribeiro FJ, Burton JN, Walker BJ, Sharpe T, Hall G, Shea TP, Sykes S, 2011 High-quality draft assemblies of mammalian genomes from massively parallel sequence data. Proc Natl Acad Sci 108: 1513–1518. 21187386 10.1073/pnas.1017351108 PMC3029755

[CLAVIJOGR217117C19] Gordon D, Huddleston J, Chaisson MJP, Hill CM, Kronenberg ZN, Munson KM, Malig M, Raja A, Fiddes I, Hillier LW, 2016 Long-read sequence assembly of the gorilla genome. Science 352: aae0344. 27034376 10.1126/science.aae0344 PMC4920363

[CLAVIJOGR217117C20] Griffiths S, Sharp R, Foote TN, Bertin I, Wanous M, Reader S, Colas I, Moore G. 2006 Molecular characterization of Ph1 as a major chromosome pairing locus in polyploid wheat. Nature 439: 749–752. 16467840 10.1038/nature04434

[CLAVIJOGR217117C21] Haas BJ, Papanicolaou A, Yassour M, Grabherr M, Blood PD, Bowden J, Couger MB, Eccles D, Li B, Lieber M, 2013 De novo transcript sequence reconstruction from RNA-seq using the trinity platform for reference generation and analysis. Nat Protoc 8: 1494–1512. 23845962 10.1038/nprot.2013.084 PMC3875132

[CLAVIJOGR217117C22] Heavens D, Accinelli GG, Clavijo B, Clark MD. 2015 A method to simultaneously construct up to 12 differently sized Illumina Nextera long mate pair libraries with reduced DNA input, time, and cost. Biotechniques 59: 42–45. 26156783 10.2144/000114310

[CLAVIJOGR217117C23] The International Wheat Genome Sequencing Consortium. 2014 A chromosome-based draft sequence of the hexaploid bread wheat (*Triticum aestivum*) genome. Science 345: 1251788. 25035500 10.1126/science.1251788

[CLAVIJOGR217117C24] Jupe F, Pritchard L, Etherington GJ, MacKenzie K, Cock PJ, Wright F, Sharma SK, Bolser D, Bryan GJ, Jones JD, 2012 Identification and localisation of the NB-LRR gene family within the potato genome. BMC Genomics 13: 75. 22336098 10.1186/1471-2164-13-75 PMC3297505

[CLAVIJOGR217117C25] Kozarewa I, Ning Z, Quail MA, Sanders MJ, Berriman M, Turner DJ. 2009 Amplification-free Illumina sequencing-library preparation facilitates improved mapping and assembly of (G+C)-biased genomes. Nat Methods 6: 291–295. 19287394 10.1038/nmeth.1311 PMC2664327

[CLAVIJOGR217117C26] Lam HYK, Clark MJ, Chen R, Chen R, Natsoulis G, O'Huallachain M, Dewey FE, Habegger L, Ashley EA, Gerstein MB, 2011 Performance comparison of whole-genome sequencing platforms. Nat Biotechnol 30: 78–82. 22178993 10.1038/nbt.2065 PMC4076012

[CLAVIJOGR217117C27] Leggett RM, Clavijo BJ, Clissold L, Clark MD, Caccamo M. 2014 NextClip: an analysis and read preparation tool for Nextera Long Mate Pair libraries. Bioinformatics 30: 566–568. 24297520 10.1093/bioinformatics/btt702 PMC3928519

[CLAVIJOGR217117C28] Li L. 2003 OrthoMCL: identification of ortholog groups for eukaryotic genomes. Genome Res 13: 2178–2189. 12952885 10.1101/gr.1224503 PMC403725

[CLAVIJOGR217117C29] Lieberman-Aiden E, van Berkum NL, Williams L, Imakaev M, Ragoczy T, Telling A, Amit I, Lajoie BR, Sabo PJ, Dorschner MO, 2009 Comprehensive mapping of long-range interactions reveals folding principles of the human genome. Science (New York NY.) 326: 289–293. 10.1126/science.1181369 PMC2858594 19815776

[CLAVIJOGR217117C30] Liu Z, Xin M, Qin J, Peng H, Ni Z, Yao Y, Sun Q. 2015 Temporal transcriptome profiling reveals expression partitioning of homeologous genes contributing to heat and drought acclimation in wheat (*Triticum aestivum L*.). BMC Plant Biol 15: 152. 26092253 10.1186/s12870-015-0511-8 PMC4474349

[CLAVIJOGR217117C31] Love RR, Weisenfeld NI, Jaffe DB, Besansky NJ, Neafsey DE. 2016 Evaluation of DISCOVAR de novo using a mosquito sample for cost-effective short-read genome assembly. BMC Genomics 17: 187. 26944054 10.1186/s12864-016-2531-7 PMC4779211

[CLAVIJOGR217117C32] Luo R, Liu B, Xie Y, Li Z, Huang W, Yuan J, He G, Chen Y, Pan Q, Liu Y, 2012 SOAPdenovo2: an empirically improved memory-efficient short-read de novo assembler. Gigascience 1: 18. 23587118 10.1186/2047-217X-1-18 PMC3626529

[CLAVIJOGR217117C33] Lykke-Andersen S, Jensen TH. 2015 Nonsense-mediated mRNA decay: an intricate machinery that shapes transcriptomes. Nat Rev Mol Cell Biol 16: 665–677. 26397022 10.1038/nrm4063

[CLAVIJOGR217117C34] Ma J, Stiller J, Berkman PJ, Wei Y, Rogers J, Feuillet C, Dolezel J, Mayer KF, Eversole K, Zheng Y-L, 2013 Sequence-based analysis of translocations and inversions in bread wheat (*Triticum aestivum L*.). PLoS One 8: e79329. 24260197 10.1371/journal.pone.0079329 PMC3829836

[CLAVIJOGR217117C35] Magoc T, Salzberg SL. 2011 FLASH: fast length adjustment of short reads to improve genome assemblies. Bioinformatics 27: 2957–2963. 21903629 10.1093/bioinformatics/btr507 PMC3198573

[CLAVIJOGR217117C36] Mapleson D, Garcia Accinelli G, Kettleborough G, Wright J, Clavijo BJ. 2017 KAT: a K-mer analysis toolkit to quality control NGS datasets and genome assemblies. Bioinformatics 33: 574–576. 27797770 10.1093/bioinformatics/btw663 PMC5408915

[CLAVIJOGR217117C37] Marcussen T, Sandve SR, Heier L, Spannagl M, Pfeifer M, Jakobsen KS, Wulff BBH, Steuernagel B, Mayer KFX, Olsen O-A, 2014 Ancient hybridizations among the ancestral genomes of bread wheat. Science 345: 1250092. 25035499 10.1126/science.1250092

[CLAVIJOGR217117C38] Moore G, Devos K, Wang Z, Gale M. 1995 Cereal genome evolution: grasses, line up and form a circle. Curr Biol 5: 737–739. 7583118 10.1016/s0960-9822(95)00148-5

[CLAVIJOGR217117C39] Mostovoy Y, Levy-Sakin M, Lam J, Lam ET, Hastie AR, Marks P, Lee J, Chu C, Lin C, Džakula Ž, 2016 A hybrid approach for de novo human genome sequence assembly and phasing. Nat Methods 13: 587–590. 27159086 10.1038/nmeth.3865 PMC4927370

[CLAVIJOGR217117C40] Panahi B, Mohammadi SA, Khaksefidi RE, Fallah Mehrabadi J, Ebrahimie E. 2015 Genome-wide analysis of alternative splicing events in *Hordeum vulgare*: Highlighting retention of intron-based splicing and its possible function through network analysis. FEBS Lett 589: 3564–3575. 26454178 10.1016/j.febslet.2015.09.023

[CLAVIJOGR217117C41] Pearce S, Huttly AK, Prosser IM, Li Y-d, Vaughan SP, Gallova B, Patil A, Coghill JA, Dubcovsky J, Hedden P, 2015 Heterologous expression and transcript analysis of gibberellin biosynthetic genes of grasses reveals novel functionality in the GA3ox family. BMC Plant Biol 15: 130. 26044828 10.1186/s12870-015-0520-7 PMC4455330

[CLAVIJOGR217117C42] Pendleton M, Sebra R, Pang AWC, Ummat A, Franzen O, Rausch T, Stütz AM, Stedman W, Anantharaman T, Hastie A, 2015 Assembly and diploid architecture of an individual human genome via single-molecule technologies. Nat Methods 12: 780–786. 26121404 10.1038/nmeth.3454 PMC4646949

[CLAVIJOGR217117C43] Pertea M, Pertea GM, Antonescu CM, Chang T-C, Mendell JT, Salzberg SL. 2015 StringTie enables improved reconstruction of a transcriptome from RNA-seq reads. Nat Biotechnol 33: 290–295. 25690850 10.1038/nbt.3122 PMC4643835

[CLAVIJOGR217117C44] Pfeifer M, Kugler KG, Sandve SR, Zhan B, Rudi H, Hvidsten TR, International Wheat Genome Sequencing Consortium, Mayer KFX, Olsen O-A. 2014 Genome interplay in the grain transcriptome of hexaploid bread wheat. Science 345: 1250091. 25035498 10.1126/science.1250091

[CLAVIJOGR217117C45] Phillips AL. 2016 Genetic control of gibberellin metabolism and signalling in crop improvement. In Annual plant reviews, Vol. 49, pp. 405–430. John Wiley & Sons, Chichester, UK.

[CLAVIJOGR217117C46] Pingault L, Choulet F, Alberti A, Glover N, Wincker P, Feuillet C, Paux E. 2015 Deep transcriptome sequencing provides new insights into the structural and functional organization of the wheat genome. Genome Biol 16: 29. 25853487 10.1186/s13059-015-0601-9 PMC4355351

[CLAVIJOGR217117C47] Putnam NH, O'Connell BL, Stites JC, Rice BJ, Blanchette M, Calef R, Troll CJ, Fields A, Hartley PD, Sugnet CW, 2016 Chromosome-scale shotgun assembly using an in vitro method for long-range linkage. Genome Res 26: 342–350. 26848124 10.1101/gr.193474.115 PMC4772016

[CLAVIJOGR217117C48] Riley R, Kimber G, Chapman V. 1961 Origin of genetic control of diploid-like behavior of polyploid wheat. J Heredity 52: 22–25.

[CLAVIJOGR217117C49] Sarris PF, Cevik V, Dagdas G, Jones JDG, Krasileva KV. 2016 Comparative analysis of plant immune receptor architectures uncovers host proteins likely targeted by pathogens. BMC Biol 14: 8. 26891798 10.1186/s12915-016-0228-7 PMC4759884

[CLAVIJOGR217117C50] Sears ER. 1966 Nullisomic-tetrasomic combinations in hexaploid wheat. In Chromosome manipulations and plant genetics, pp. 29–45. Springer, Boston, MA.

[CLAVIJOGR217117C51] Shewry PR, Tatham AS, Barro F, Barcelo P, Lazzeri P. 1995 Biotechnology of breadmaking: unraveling and manipulating the multi-protein gluten complex. Biotechnology 13: 1185–1190. 9636290 10.1038/nbt1195-1185

[CLAVIJOGR217117C52] Song L, Sabunciyan S, Florea L. 2016 CLASS2: accurate and efficient splice variant annotation from RNA-seq reads. Nucleic Acids Res 44: e98. 26975657 10.1093/nar/gkw158 PMC4889935

[CLAVIJOGR217117C53] Staiger D, Brown JWS. 2013 Alternative splicing at the intersection of biological timing, development, and stress responses. Plant Cell 25: 3640–3656. 24179132 10.1105/tpc.113.113803 PMC3877812

[CLAVIJOGR217117C54] Stanke M, Morgenstern B. 2005 AUGUSTUS: a web server for gene prediction in eukaryotes that allows user-defined constraints. Nucleic Acids Res 33: W465–W467. 15980513 10.1093/nar/gki458 PMC1160219

[CLAVIJOGR217117C55] Steuernagel B, Jupe F, Witek K, Jones JDG, Wulff BBH. 2015 NLR-parser: rapid annotation of plant NLR complements. Bioinformatics 31: 1665–1667. 25586514 10.1093/bioinformatics/btv005 PMC4426836

[CLAVIJOGR217117C57] Trapnell C, Williams BA, Pertea G, Mortazavi A, Kwan G, van Baren MJ, Salzberg SL, Wold BJ, Pachter L. 2010 Transcript assembly and quantification by RNA-seq reveals unannotated transcripts and isoform switching during cell differentiation. Nat Biotechnol 28: 511–515. 20436464 10.1038/nbt.1621 PMC3146043

[CLAVIJOGR217117C58] Treangen TJ, Salzberg SL. 2012 Repetitive DNA and next-generation sequencing: computational challenges and solutions. Nat Rev Genet 13: 36–46. 10.1038/nrg3117 PMC3324860 22124482

[CLAVIJOGR217117C59] Vogel JP, Garvin DF, Mockler TC, Schmutz J, Rokhsar D, Bevan MW, Barry K, Lucas S, Harmon-Smith M, Lail K, 2010 Genome sequencing and analysis of the model grass *Brachypodium distachyon*. Nature 463: 763–768. 20148030 10.1038/nature08747

[CLAVIJOGR217117C60] Wagner GP, Kin K, Lynch VJ. 2013 A model based criterion for gene expression calls using RNA-seq data. Theory Biosci 132: 159–164. 23615947 10.1007/s12064-013-0178-3

[CLAVIJOGR217117C61] Weisenfeld NI, Yin S, Sharpe T, Lau B, Hegarty R, Holmes L, Sogoloff B, Tabbaa D, Williams L, Russ C, 2014 Comprehensive variation discovery in single human genomes. Nat Genet 46: 1350–1355. 25326702 10.1038/ng.3121 PMC4244235

[CLAVIJOGR217117C62] Weisenfeld NI, Kumar V, Shah P, Church D, Jaffe DB. 2016 Direct determination of diploid genome sequences. Genome Res (this issue). 10.1101/gr.214874.116. PMC5411770 28381613

[CLAVIJOGR217117C63] Zhang C, Yang H, Yang H. 2015 Evolutionary character of alternative splicing in plants. Bioinformatics Biol Insights 9: 47–52. 10.4137/BBI.S33716 PMC4721685 26819552

[CLAVIJOGR217117C64] Zimin AV, Puiu D, Luo M-C, Zhu T, Koren S, Marcais G, Yorke JA, Dvorak J, Salzberg SL. 2017 Hybrid assembly of the large and highly repetitive genome of *Aegilops tauschii*, a progenitor of bread wheat, with the mega-reads algorithm. Genome Res (this issue). 10.1101/gr.213405.116. PMC5411773 28130360

