# Supplementary material for: An improved assembly and annotation of the allohexaploid wheat genome identifies complete families of agronomic genes and provides genomic evidence for chromosomal translocations
Source: Genome Res. 2017 May;27(5):885–96. doi: 10.1101/gr.217117.116 (PMC5411782; doi:10.1101/gr.217117.116)
Supplement: Supplemental Material [file supp_27_5_885__index.html]

An improved assembly and annotation of the allohexaploid wheat genome identifies complete families of agronomic genes and provides genomic evidence for chromosomal translocations — Supplemental Material 

# An improved assembly and annotation of the allohexaploid wheat genome identifies complete families of agronomic genes and provides genomic evidence for chromosomal translocations

## Supplemental Material

- Supplemental\_File\_S2.xlsx
- Supplemental\_File\_S3.xlsx
- Supplemental\_File\_S4.tsv
- Supplemental\_File\_S5.tsv
- Supplemental\_File\_S6.tsv
- Supplemental\_File\_S7.xlsx
- Supplemental\_File\_S8.txt
- Supplemental\_File\_S9.xlsx
- Supplemental\_File\_S10.xlsx
- Supplemental\_File\_S11.xlsx
- Supplemental\_File\_S12.tsv
- Supplemental\_File\_S13.xlsx
- Supplemental\_File\_S14.xlsx
- Supplemental\_File\_S23.txt
- Supplemental\_Files\_S15-S26.zip
- Supplemental\_Information\_S1.pdf
