# Supplementary material for: An improved assembly and annotation of the allohexaploid wheat genome identifies complete families of agronomic genes and provides genomic evidence for chromosomal translocations
Source: Genome Res. 2017 May;27(5):885–96. doi: 10.1101/gr.217117.116 (PMC5411782; doi:10.1101/gr.217117.116)
Supplement: Supplemental Material [file supp_gr.217117.116_Supplemental_Information_S1.pdf]

Supplemental material for *An improved assembly and annotation of the allohexaploid wheat genome identifies complete families of agronomic genes and provides genomic evidence for chromosomal translocations.*

March 14, 2017

Contents

1 Germplasm and DNA isolation 3
1.1 Germplasm . . . 3
1.2 DNA isolation . . . 3

2 Sequencing library preparation 4
2.1 Amplification-free paired-end library construction protocol . . . 4
2.2 Tight, Amplification-free, Large insert Libraries (TALL) paired-end library construction protocol . . . 4
2.3 Long mate-pair library construction protocol . . . 4

3 RNA sequencing 6
3.1 Stranded RNA sequencing on Illumina HiSeq2500 . . . 6
3.2 Isoform Sequencing (Iso-Seq™) . . . 6

4 Genomic assembly and scaffolding 7
4.1 Contig assembly . . . 7
4.2 Scaffolding . . . 7
4.3 Contamination screening and filtering . . . 9
4.4 Chromosome arm binning . . . 9
4.5 Sequence length and content filter . . . 9
4.6 Assignment of scaffold identifiers . . . 9
4.7 Comparison of TGACv1 scaffolds to Chapman and CSS assemblies . . . 9
4.8 Assembly validation . . . 11
4.8.1 Contig validation by mate-pair link support . . . 11
4.8.2 Scaffold validation by gene order between 3B and TGACv1 3B sequences . . . 11
4.9 Assessment of chromosome arm assignment accuracy . . . 12

5 Integration with genetic maps and chromosomal alignments 13

6 Detection and confirmation of chromosomal translocations 14
6.1 Detection of translocations from OrthoMCL output . . . 14
6.2 PCR assays of suspected translocations . . . 14
6.3 Cross-validation of translocations by using the genetic map . . . 15

7 Repeat analysis 17

8 Construction of the wheat gene set 18
8.1 Reference guided transcriptome reconstruction . . . 18
8.1.1 Alignment of Illumina RNA-seq data . . . 18
8.1.2 Alignment of PacBio RNA-seq data . . . 19
8.1.3 Transcript assembly . . . 19
8.2 Gene predictor training . . . 20
8.3 Gene prediction using evidence guided AUGUSTUS . . . 20
8.3.1 Generation of external hints for gene prediction . . . 20
8.3.2 Gene prediction . . . 21
8.4 Gene model refinement . . . 22

8.5 Assignment of gene biotypes and confidence classification . . . 22
8.5.1 Cross species protein similarity ranking . . . 23
8.5.2 Wheat transcript support ranking . . . 23
8.5.3 Assignment of a locus biotype . . . 23
8.5.4 Removal of spurious genes . . . 24
8.5.5 Assignment of high and low confidence tags . . . 24
8.5.6 Assignment of a representative gene model . . . 26
8.5.7 Assessment of the TGACv1 annotation . . . 26
8.5.8 Evaluation of non-coding RNAs . . . 29
8.6 Alternative splicing analysis . . . 30
8.7 Functional annotation of protein coding transcripts . . . 30
8.8 Data Access . . . 30

9 Proteomics 31

10 Orthologous gene family analyses 32
10.1 OrthoMCL gene family clustering of wheat subgenome genes . . . 32
10.2 OrthoMCL gene family clustering of the bread wheat genome and related species . . . 32
10.3 GO over-/under-representation for specific groups/singletons . . . 33
10.4 Expanded gene families in OrthoMCL and GO over-representation within . . . 33

11 Gene expression analyses 35
11.1 Expression quantification and analysis . . . 35
11.1.1 Gene expression quantification . . . 35
11.1.2 Differential gene expression analysis . . . 35
11.1.3 Visualisation of gene expression . . . 36
11.2 Gene expression across 17 diverse RNA-seq studies . . . 36
11.3 Gene expression patterns across chromosome regions . . . 36
11.4 Analysis of homoeolog gene expression in stress conditions . . . 36
11.5 Homoeologous gene expression analysis . . . 37
11.6 Gene expression in syntenic loci . . . 38

12 Gene families of agronomic importance 41
12.1 Disease resistance genes . . . 41
12.2 Gluten genes . . . 41
12.3 Gibberellin genes . . . 42
12.4 BAC analysis . . . 42

13 Authors' contributions 43

14 File list 43

15 References 44

1 Germplasm and DNA isolation

1.1 Germplasm

A single seed descent line of *Triticum aestivum* Chinese Spring (called CS42) was used for DNA extraction. The provenance of the line has been traced to original Sears material.

1.2 DNA isolation

High molecular weight wheat DNA was isolated from leaf material of 2–3 week old CS42 plants that had been kept in the dark for 48 hours to reduce starch levels. Leaf material (60–80g) was frozen in liquid N2 and ground to a fine powder in a mortar and pestle. Ground leaf tissue was transferred into ice-cold SEB buffer + mercaptoethanol (ME), using a ratio of 15ml SEB+ME per gram of leaf material. The leaf tissue and buffer was gently mixed for 20 seconds every 2 minutes for 10–15 minutes on ice, and then filtered twice through two layers of Miracloth with gentle squeezing. 1/20 volume of SEB+ME+ 10% v/v Triton X100 was added and mixed for 20 seconds every 2 minutes for a total of 10 minutes. The mixture was centrifuged at 600× g for 20 minutes at 4°C in 250ml polypropylene centrifuge bottles. The supernatant was removed gently with a pipette, and 1ml SEB+ME added to each pellet to gently resuspend it. SEB+ME was added to a total of 20ml and the crude nuclei were centrifuged again. This step was repeated twice, and washed nuclei were resuspended in a total of 7.5ml SEB+ME. 20% w/v SDS was added to final concentration of 2% w/v, and the mixture inverted gently to lyse the nuclei. The lysed nuclei were heated at 60°C for 10 minutes in a waterbath, cooled to room temperature, and 5M sodium perchlorate added to a final concentration of 1M to further disrupt protein-nucleic acid interactions. The lysate was centrifuged at 500× g for 20 minutes at 10°C to pellet starch grains, and the supernatant transferred to a new 15ml tube using a cut-off 1ml pipette tip to minimise shearing DNA. The nucleic acid solution was extracted with an equal volume of phenol/chloroform/isoamyl alcohol (25:24:1) and gently rocked (18 cycles/ minutes for 15 minutes. The mixture was centrigued at 3000× g for 10 minutes in a swinging bucket rotor, the supernatant transferred to a new tube, and re-extracted. The final aqueous phase was dialysed in TE pH 7.0 at 4°C overnight. RNase T1 and RNAse A were added to the dialysate to 50U/ml and 50µg/ml respectively, gently mixed by inversion, and incubated at 37°C for 45–60 minutes. Proteinase K was added to a final concentration of 150µg/ml and incubated for a further 45–60 minutes. The DNA was then extracted twice with phenol/chloroform/iso-amyl alcohol, and DNA was precipitated from the final aqueous phase by the addition of 1/10 volume of 3M sodium acetate (pH 5.2) and 2.5 volumes of ethanol. DNA was precipitated by centrifugation at 5000× g for 30 minutes at 4°C. The pellet was rinsed in 1ml of 70% ethanol, air dried for 1 hour, and resuspended in TE buffer. Final yields were 50–100µg DNA per 100g of leaf material.

Buffers

TKE	
Tris (0.1M)	6.055g
KCI (1M)	37.275g
EDTA (0.1M)	18.61g
MBG water to	500ml

Store at 4°C. Do not adjust pH.

SEB	
Sucrose	171.2g
PEG 800	1.2g
Carbamic acid	1.3g
Spermine	0.35g (Place at 37°C if forms a solid block)
Spermidine	1g
TKE	100ml
MBG water	1000ml

Adjust to pH 9.5 with concentrated HCl if needed.

Add 2ml Mercaptoethanol (BME) to the SEB just before use.

2 Sequencing library preparation

2.1 Amplification-free paired-end library construction protocol

A total of 600ng of DNA was sheared in a 60µl volume on a Covaris S2 (Covaris, Massachusetts, USA) for 1 cycle of 40 seconds with a duty cycle of 5%, cycles per burst of 200 and intensity of 3. The fragmented molecules were then end repaired in 100µl volume using the NEB End Repair Module (NEB, Hitchin, UK) incubating the reaction at 22°C for 30 minutes. Post incubation 58µl beads of CleanPCR beads (GC Biotech, Alphen aan den Rijn, The Netherlands) were added using a positive displacement pipette to ensure accuracy and the DNA precipitated onto the beads. They were then washed twice with 70% ethanol and the end repaired molecules eluted in 25µl Nuclease free water (Qiagen, Manchester, UK). End repaired molecules were then A tailed in 30µl volume using in the NEB A tailing module (NEB) incubating the reaction at 37°C for 30 minutes. To the A tailed library molecules 1µl of an appropriate Illumina TruSeq Index adapter (Illumina, San Diego, USA) was added and mixed then 31µl of Blunt/ TA ligase (NEB) added and incubated at 22°C for 10 minutes. Post incubation 5µl of stop ligation was added and then the reaction incubated at room temperature for 5 minutes. Following this incubation 67µl beads of CleanPCR beads (GC Biotech, Alphen aan den Rijn, The Netherlands) were added and the DNA precipitated onto the beads. They were then washed twice with 70% ethanol and the library molecules eluted in 100µl nuclease free water. Two further CleanPCR bead based purifications were undertaken to remove any adapter dimer molecules that may have formed during the adapter ligation step. The first with 0.9× volume beads, the second with 0.6× and the final library eluted in 25µl Resuspension Buffer (Illumina).

Library QC was performed by running a 1µl aliquot on a High Sensitivity BioAnalyser chip (Agilent, Stockport, UK) and the DNA concentration measured using the High Sensitivity Qubit (Thermo Fisher, Cambridge, UK). To determine the number of viable library molecules the library was subjected to quantification by the Kappa qPCR Illumina quantification kit (Kapa Biosystems, London, UK) and a test lane run at 10pM on a MiSeq (Illumina) with 2×300bp reads to allow the library to be characterised prior to generation of the 60× coverage required on the Hiseq2500s (Illumina) with a 2×250bp read metric.

2.2 Tight, Amplification-free, Large insert Libraries (TALL) paired-end library construction protocol

A total of 3µg of DNA was sheared in a 60µl volume on a Covaris S2 (Covaris, Massachusetts, USA) for 1 cycle of 40 seconds with a duty cycle of 5%, cycles per burst of 200 and intensity of 3. The fragmented DNA was then subjected to size selection on a Blue Pippin (Sage Science, Beverly, USA). The 40µl in each of collection wells was replaced with fresh buffer and the separation and elution current checked prior to loading the sample. To 30µl of the end repaired molecules 10µl of R2 marker solution was added and then loaded onto a 1.5% Cassette. The Blue Pippin was configured to collect fragments at 800bp using the tight settings. Post size selection, the 40µl from the collection well was recovered and the size isolated estimated on High Sensitivity BioAnalyser Chip and DNA concentration determined using a Qubit HS Assay.

The size selected molecules were then end repaired in 100µl volume using the NEB End Repair Module (NEB, Hitchin, UK) incubating the reaction at 22°C for 30 minutes. Post incubation 100µl beads of CleanPCR beads (GC Biotech, Alphen aan den Rijn, The Netherlands) were added and the DNA precipitated onto the beads. They were then washed twice with 70% ethanol and the end repaired molecules eluted in 25µl Nuclease free water (Qiagen, Manchester, UK).

End repaired molecules were then A tailed in 30µl volume using in the NEB A tailing module (NEB) incubating the reaction at 37°C for 30 minutes. To the A tailed library molecules 1µl of an appropriate Illumina TruSeq Index adapter (Illumina, San Diego, USA) was added and mixed then 31µl of Blunt/ TA ligase (NEB) added and incubated at 22°C for 10 minutes. Post incubation 5µl of stop ligation was added and then the reaction incubated at room temperature for 5 minutes. Following this incubation 67µl beads of CleanPCR beads (GC Biotech, Alphen aan den Rijn, The Netherlands) were added and the DNA precipitated onto the beads. They were then washed twice with 70% ethanol and the library molecules eluted in 100µl nuclease free water. Two further 1× CleanPCR bead based purifications were undertaken to remove any adapter dimer molecules that may have formed during the adapter ligation step and the final library eluted in 25µl Resuspension Buffer (Illumina).

Library QC was performed by running a 1µl aliquot on a High Sensitivity BioAnalyser chip (Agilent, Stockport, UK) and the DNA concentration measured using the High Sensitivity Qubit (Thermo Fisher, Cambridge, UK). To determine the number of viable library molecules the library was subjected to quantification by the Kappa qPCR Illumina quantification kit (Kapa Biosystems, London, UK) and then sequenced on the Hiseq2500s (Illumina) with a 2×150bp read metric.

2.3 Long mate-pair library construction protocol

For the Tagmentation reactions 3µg and 6µg of Genomic DNA was prepared in 308µl and then 80µl 5× Tagment Buffer Mate Pair (Illumina) added followed by 12µl Mate Pair Tagmentation Enzyme (Illumina) and the reaction gently vortexed to mix. This was then incubated for 30 minutes at 55°C, 100µl of Neutralize Tagment Buffer (Illumina) added and then incubated at room temperature for 5 minutes. A 1× volume bead clean-up was performed with CleanPCR beads and the DNA eluted in 165µl of Nuclease free Water. A 1µl aliquot was run on a BioAnalyser 1200 chip and DNA concentration determined using a Qubit HS Assay.

Strand Displacement was performed by combining 162µl of tagmented DNA, 20µl 10× Strand Displacement Buffer (Illumina), 8µl dNTPs (Illumina) and 10µl Strand Displacement Polymerase (Illumina). This was then incubated at room temperature for 30 minutes. A 0.75× volume bead clean-up was performed with CleanPCR beads and the DNA eluted in 16µl of Nuclease free Water and the eluted DNA from the 3µg and 6µg reactions pooled. A 1µl aliquot was diluted 1:6 and run on a BioAnalyser 1200 chip and DNA concentration determined using a Qubit HS Assay.

Size selection was performed on a Sage Science ELF (Sage Science, Beverly, USA). The 30µl in each of collection wells was replaced with fresh buffer and the collection and elution current checked prior to loading the sample. To 30µl of the pooled Strand Displaced reaction 10µl of loading solution was added and then loaded onto a 0.75% Cassette which was configured to separate the sample for 3 hours 30 minutes and then eluting each fraction for 35 minutes. Post size selection, the 30µl from each of the 12 collection wells was recovered and the DNA concentration determined using a Qubit HS Assay.

Circularisation was performed by combining 30µl of size fractionated DNA, 12.5µl of 10× circularisation buffer (Illumina), 3µl Circularisation Enzyme (Illumina) and 85µl nuclease free water. These were then incubated at 30°C overnight. Linear DNA was digested by adding 3.75µl Exonuclease (Illumina) and incubating at 37°C for 30 minutes followed by 70°C for 30 minutes to denature the enzyme and 5µl of stop ligation (Illumina) added. During exonuclease treatment 240µl of M280 Dynabeads (Thermo Fisher) were prepared by washing twice with 600µl Bead Bind Buffer (Illumina) before resuspending in 1560µl Bead Bind Buffer. Circularised DNA was then sheared in a 130µl volume on a Covaris S2 for 2 cycles of 37secs with a duty cycle of 10%, cycles per burst of 200 and intensity of 4.

To 130µl fragmented DNA 130µl of washed M280 beads was added, mixed and then placed on a lab rotator at room temperature for 20 minutes. Library molecules bound to M280 beads were then washed four times with 200µl Bead Washer Buffer (Illumina) and twice with 200µl Resuspension Buffer (Illumina).

A master mix containing 1105µl nuclease free water, 130µl 10× End Repair Reaction Buffer (NEB, Hitchin, UK) and 65µl end repair enzyme mix (NEB) was prepared and 100µl added to each tube, mixed with the beads and incubated at room temperature for 30 minutes. End repaired library molecules bound to M280 beads were then washed four times with 200µl Bead Washer Buffer and twice with 200µl Resuspension Buffer.

A master mix containing 325µl nuclease free water, 39µl A Tailing 10× Reaction Buffer (NEB) and 26µl A tailing enzyme mix (NEB) was prepared and 30µl added to each tube, mixed with the beads and incubated at 37°C for 30 minutes. To the A tailed library molecules 1µl of the appropriate Illumina Index adapter (Illumina) was added and mixed then 31µl of Blunt/ TA ligase (NEB) added and incubated at room temperature for 10 minutes. Post incubation 5µl of stop ligation added and then the adapter ligated library molecules bound to M280 beads were then washed four times with 200µl Bead Washer Buffer and twice with 200µl Resuspension Buffer.

A master mix containing 240µl nuclease free water, 300µl 2× Kappa HiFi (Kappa Biosystems) and 60µl Illumina Primer Cocktail (Illumina) was prepared and 50µl added to each tube, mixed with the beads and the contents, including beads, transferred to a 200µl PCR tube. Each sample was then subjected to amplification on a Veriti Thermal Cycler (Thermo Fisher) with the following conditions: 98°C for 3 minutes, 8, 10 or 12 cycles of PCR depending upon copy number entering circularisation of 98°C for 10 seconds, 60°C for 30 seconds, 72°C for 30 seconds followed by 72°C for 5 minutes and Hold at 4°C.

Post amplification the PCR tubes were placed on a magnetic plate, the beads allowed to pellet and then 45µl of the PCR transferred to a 2ml Lobind Eppendorf Tube. To this 31.5µl beads of CleanPCR beads were added to precipitate the DNA, the beads washed twice with 70% ethanol and the final library eluted in 20µl resuspension buffer. Library QC was performed by running a 1µl aliquot on a High Sensitivity BioAnalyser chip (Agilent) and the DNA concentration measured using the High Sensitivity Qubit (Thermo Fisher). Each library was then equimolar pooled (except for the largest insert library which was considerably weaker than the others which was at 10% concentration) based on DNA concentration. The quantification of the pool was determined by the Kappa qPCR Illumina quantification kit (KAPPA) with the pool run at 10pM on a MiSeq with a 2×300bp reads read metric.

Reads generated were then processed through NextClip which takes LMP FASTA reads and looks to categorise them into four groups based on the presence of the Nextera adapter junction sequence. Category A pairs contain the adaptor in both reads, Category B pairs contain the adaptor in only read 2, Category C pairs contain the adaptor in only read 1, Category D pairs do not contain the adaptor in either read. NextClip also uses a k-mer-based approach to estimate the PCR duplication rate while reads are examined. Filtered reads in categories A, B and C were then mapped back to the Wheat Chromosome 3B reference using BWA mem with the default parameters. This uses the reference sequence and measures from the leftmost to the rightmost aligned bases within the reads to determine the insert size.

Once characterised the libraries with inserts centred at 9kbp (Fraction 4) and 11kbp (Fraction 3) were then sequenced to greater depth as 2×250bp reads on HiSeq2500s.

3 RNA sequencing

3.1 Stranded RNA sequencing on Illumina HiSeq2500

Quality checked libraries were quantified to range from 2.2nM to 9.87nM. Each library was then diluted to 2nM with NaOH and 5µl transferred into 995µl HT1 (Illumina) to give a final concentration of 10pM. 135µl of the diluted library pool was then transferred into a 200µl strip tube, spiked with 1% PhiX Control v3 and placed on ice before loading onto the Illumina cBot. The library was hybridised to the flow cell using HiSeq Rapid Paired End Cluster Generation Kit v2, following the Illumina RR_TemplateHyb_FirstExt_VR recipe. Following the hybridisation procedure, the flow cell was loaded onto the Illumina HiSeq2500 instrument following the manufacturer's instructions. The sequencing chemistry utilised was HiSeq Rapid SBS v2 using HiSeq Control Software 2.2.58 and RTA 1.18.64. Each library was run across a single lane for 250 cycles for each paired end read. Reads in bcl format were demultiplexed based on the 6bp Illumina index by CASAVA 1.8, allowing for a one base-pair mismatch per library, and converted to FASTQ format by bcl2fastq.

3.2 Isoform Sequencing (Iso-Seq™)

The procedures used followed the Pacific Biosciences protocol. `http://www.pacb.com/wp-content/uploads/Procedure-Checklist-Isoform-Sequencing-Iso-Seq-Analysis-using-the-Clontech-SMARTer-PCR-cDNA-Synthesis-Kit-and-SageELF-Size-Selection-System.pdf`

4 Genomic assembly and scaffolding

4.1 Contig assembly

We generated 1.1 billion 250bp paired-end reads from two PCR-free CS42 libraries (see Table S4.1) which provided 32.78× coverage of the CS42 genome (approx. 30× 31-mer coverage). Insert size distributions of each library were checked by mapping to the CS42 chromosome 3B pseudo-molecule (Choulet et al., 2014) using the DRAGEN co-processor (EdicoGenome, 2014).

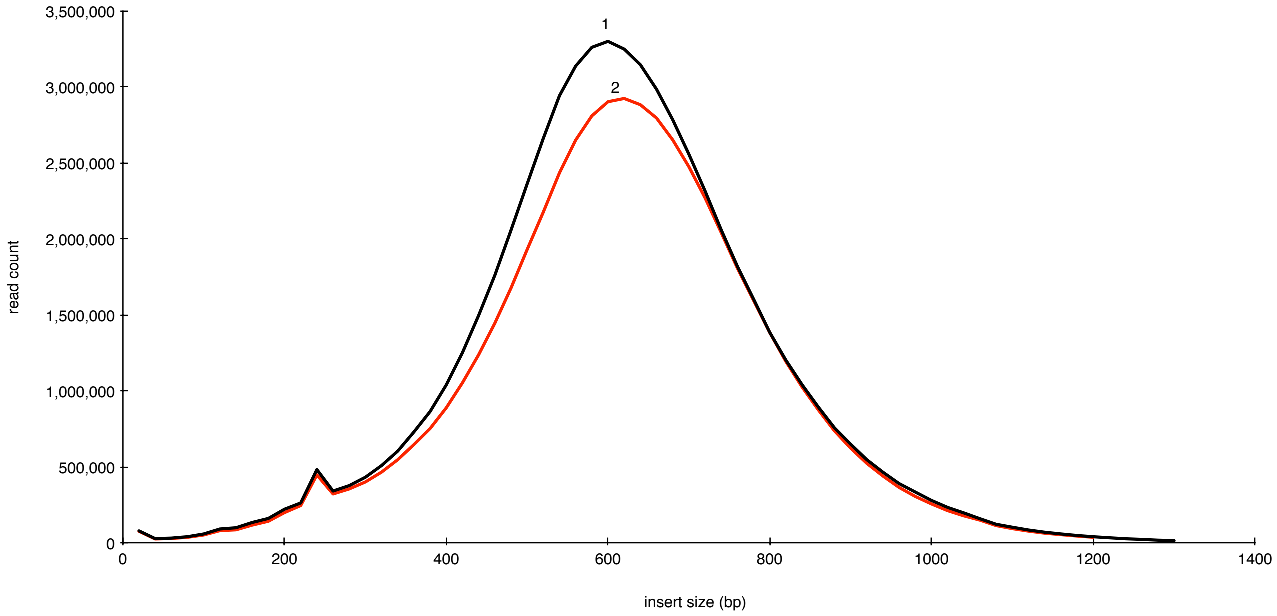

Figure S4.1: Insert size distributions of the two PE libraries.

A method based on DISCOVAR *de novo* (Weisenfeld et al., 2014) was chosen to assemble contigs as this approach utilises PCR-free libraries to reduce coverage bias, and uses long 250bp reads generated by the latest Illumina sequencing technology. Originally developed to assemble human genomes, the algorithm is designed to retain the majority of the variation present in the reads when generating the assembly, including variation between homologous chromosomes and repeat copies. This is important when assembling a repeat-rich hexaploid genome such as wheat to prevent collapsing of repeats and homologous/homoeologous regions during assembly. Contig assembly starts by correcting errors in the reads by creating “friend stacks” for each read in a read pair, then retaining only “true friends”, reads that perfectly match the original read pair (with an offset). A consensus sequence is called for each stack, in most cases including the gap between the read pairs consistent with the library fragmentation step. Overlaps between each stack are used to generate a ‘joint consensus’ for the original DNA fragment, typically yielding a single unambiguous joint consensus, a “closed pair”. The unipath graph is created from the consensus sequences, simplified to remove artifacts from the laboratory process, then the closed pair sequences are applied to the graph to join paths that overlap and pull apart regions containing collapsed repeats. The version of the contigger used is available in Github (`https://github.com/bioinfologics/w2rap-contigger/releases/tag/CS42_TGACv1`) and is fully described elsewhere (Clavijo et al., 2017). Contigs were QC’ed using KAT (Mapleson et al., 2017) `spectra-cn` plots to check motif representation. Importantly, our data generation was tailored to generate maximum complexity, precisely sized, low bias sampling.

4.2 Scaffolding

Multiple Nextera Long Mate Pair libraries were constructed as described above, QC’ed by alignment to the 3B pseudomolecule, and chosen for sequencing as described in our published LMP protocol (see Table S4.2; Heavens et al. (2015)). Raw reads were pre-processed using a pipeline based on NextClip (Leggett et al., 2014). Briefly, this pipeline merges overlapping read pairs with FLASH (Magoc and Salzberg, 2011), generates a read 2 by reverse complementing the read 1 sequence, then runs Nextclip to identify and trim reads containing the Nextera adaptor.

Table S4.1: Paired-end library details

	Library type	Read count	Read length (bps)	Insert size (bps)	Read coverage
1	PCR-free	658,890,225	250	620	19.38
2	PCR-free	455,733,257	250	600	13.4

Table S4.2: Summary of library sequencing. *Library 4 was sequenced twice, once generating 150bp reads and once generating 250bp reads.

Library	Type	Read count	Read length (bp)	Insert size (bp)	Read coverage	Fragment coverage
1	TALL	118,575,256	150	690	2.09	4.81
2	TALL	309,422,248	150	690	5.46	12.56
3	TALL	434,404,265	150	690	7.67	17.63
4*	MP	151,086,835	150	2,480	2.67	22.04
		508,236,686	250	2,480	14.95	74.14
5	MP	170,061,926	150	4,300	3.00	43.02
6	MP	142,304,055	150	5,250	2.51	43.95
7	MP	432,253,166	250	9,300	12.71	236.47
8	MP	173,921,104	250	9,180	5.12	93.92
9	MP	404,721,706	250	11,600	11.90	276.16

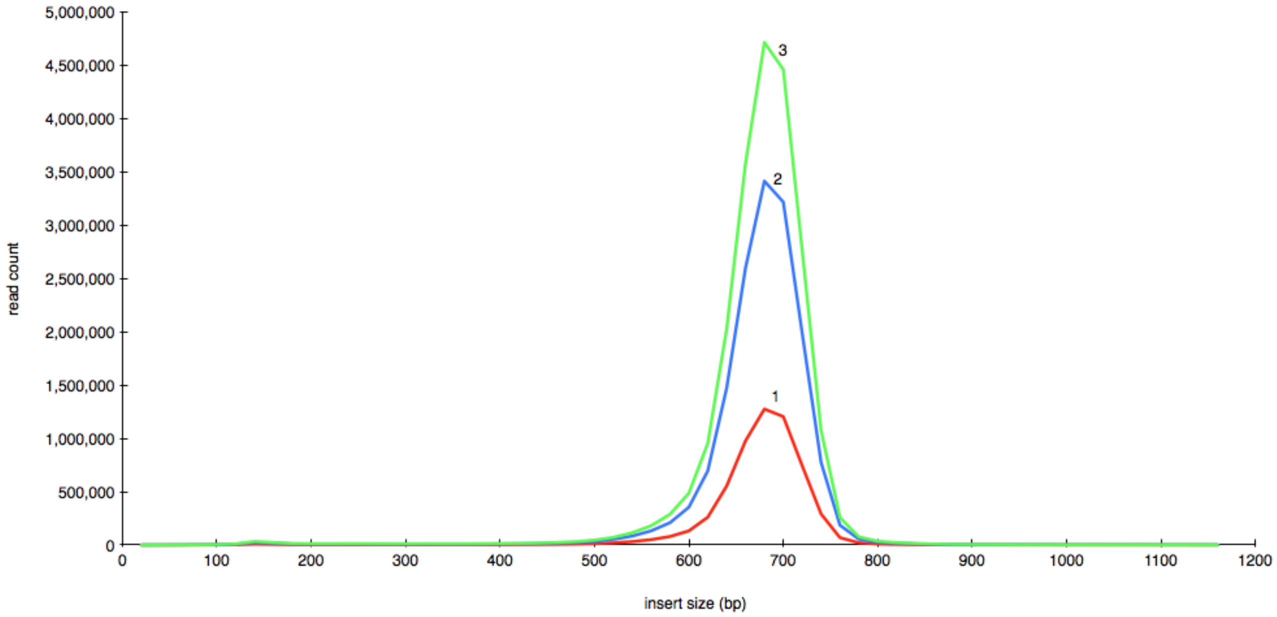

Figure S4.2: Insert size distribution for TALL libraries.

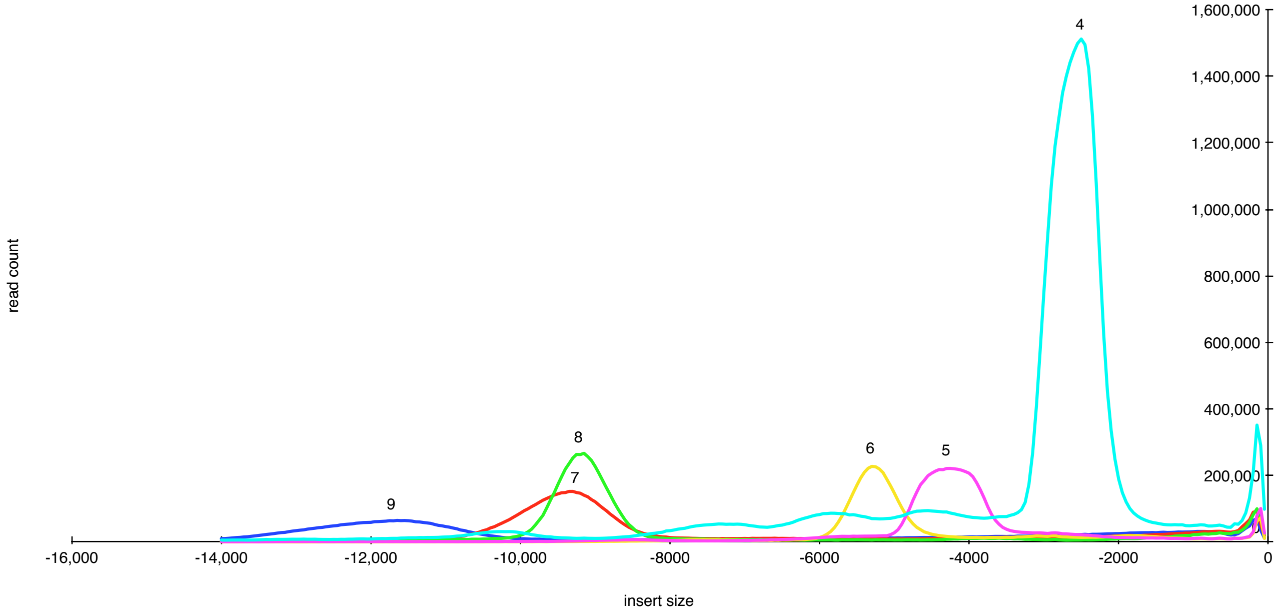

Figure S4.3: Insert size distribution for LMP libraries.

Table S4.3: Summary contig and N-remapped scaffolds for the TGACv1 assembly of Chinese Spring 42.

	Size (Gb)	Sequence count (≥500bp)	N20 (kb)	N50 (kb)	N80 (kb)	NG50 (kb)	L50	%N
Contigs	13.26	2,977,539	40.7	16.7	3.1	8.7	200,473	0.1
Scaffolds	14.07	1,333,497	175.6	83.9	25.4	63.1	47,111	5.6

In addition to the PE reads used to generate contigs, three Tight, Amplification-free, Large insert Library (TALL) and six mate-pair libraries were used for scaffolding. The TALL library protocol generates paired-end reads with a tight insert-size distribution without PCR-amplification and provided additional coverage for scaffolding. Contigs were scaffolded using SOAPdenovo2 (Luo et al., 2012). A *k*-mer length of 71 was used for the prepare and mapping stage. SOAPdenovo2 replaces N-stretches (gaps) in contigs with Cs and Gs during scaffolding, so to correct this contigs were mapped back to the scaffolds and the gaps converted back to Ns. Contig and scaffold contiguity statistics are shown in Table S4.3.

4.3 Contamination screening and filtering

The scaffolds were checked for contamination against the NCBI nucleotide database using BLAST+ and the results joined to NCBI's taxonomy database. Filtering was applied to show hits of more than 98% identity over 90% of scaffold length. From this list, scaffolds identified with a taxonomy containing "BEP" (the grass BEP clade), "Poales" (the order encompassing grasses) or "eudicotyledons" (the dicot group of angiosperms) were kept and the remaining scaffolds were considered to be contamination. These were mainly short contigs containing PhiX.

4.4 Chromosome arm binning

Scaffolds were classified into chromosome-arm bins using arm-specific Chromosome Survey Sequence (CSS) reads (The International Wheat Genome Sequencing Consortium, 2014). Scaffolds from 3B were not separated into short/long arm bins as individual arm datasets were not generated for this chromosome in the CSS project. The `sect` method of KAT was used to compute kmer coverage over each scaffold using each CSS read set. Each non-repetitive kmer in a scaffold was scored proportionally to coverage on each CSS arm and scaffolds were classified using the following set of rules:

1. Scaffolds with less than 10% of the kmers producing a vote were left as unclassified (marked as Chromosome arm "U"). These are mostly small and/or repetitive sequences.
2. Scaffolds with a top score towards a CSS set at least double the second top score were classified to the highest scoring chromosome arm.
3. Scaffolds with a top score towards a CSS set less than double the second top score were left as unclassified (marked as Chromosome arm "U", but with the two top scores and CSS sets included in the sequence name). This category contains scaffolds that are classified as combinations of the two arms from the same chromosome, probably due to imprecise identification during flow-sorting. It also contains scaffolds from regions of the genome with specific flow-sorting biases, and assembly chimeras.

4.5 Sequence length and content filter

Rather than using a simple length cutoff to include scaffolds in the final assembly, a content filter was applied to the scaffolds classified into each chromosome-arm bin to ensure short scaffolds containing unique content were not excluded from the assembly. Scaffolds were sorted by length, longest first. Scaffolds longer than 5kbp were automatically added to the assembly. Scaffolds between 5kbp and 500bp were added from longest to smallest if 20% of the kmers in the scaffold were not already present in the assembly. Scaffolds shorter than 500bp were excluded.

4.6 Assignment of scaffold identifiers

For assigned scaffolds, the arm assignment is included in the FASTA identifier. For unassigned scaffolds with more than 10% voting kmers, the highest and second highest vote is included in the FASTA identifier to indicate possible arms. Per chromosome statistics for the final classified scaffolds are given in Table S4.4.

4.7 Comparison of TGACv1 scaffolds to Chapman and CSS assemblies

We compared our 3B scaffolds to 3B scaffolds from the Chapman (Chapman et al., 2015) and CSS (The International Wheat Genome Sequencing Consortium, 2014) assemblies. Although there are more scaffolds in the TGACv1 3B assembly than the Chapman 3B scaffolds, they are more contiguous and represent a much higher portion of the chromosome. To compare gene content between assemblies, the 7703 genes identified on 3B (Choulet et al., 2014) were aligned to the 3B scaffolds from each assembly using GMap (Wu and Watanabe, 2005). Genes were counted if they aligned with at least 95% identity over 80% of their length. We could align 91.2% of 3B genes to our 3B scaffolds compared to around 70% that aligned to Chapman and CSS 3B scaffolds indicating the increased completeness of our assembly.

Table S4.4: Assembly statistics for classified scaffolds.

Arm	Total (bp)	N20	N50	N80	N%	Count
1AL	355,144,189	159,693	80,107	30,798	5.57	19,140
1AS	200,141,416	176,516	85,799	32,413	5.48	11,382
1BL	427,850,462	212,050	105,411	41,787	5.43	19,349
1BS	224,120,373	204,783	99,660	39,287	5.36	11,813
1DL	292,316,462	127,480	65,923	23,018	6.59	19,204
1DS	155,677,507	123,950	62,097	19,441	6.74	12,849
2AL	408,449,610	164,629	84,674	33,270	5.49	19,410
2AS	318,533,889	183,072	90,023	33,061	5.40	17,435
2BL	423,469,708	227,122	117,486	45,691	5.14	16,714
2BS	317,593,121	215,046	108,705	45,716	5.19	12,136
2DL	335,204,207	133,166	70,105	26,700	6.67	19,424
2DS	245,159,861	140,704	72,904	24,794	6.56	16,533
3AL	381,464,830	165,249	84,656	33,372	5.64	17,063
3AS	277,280,281	188,759	93,882	40,580	5.27	10,234
3B	789,970,040	223,860	116,546	47,041	5.13	29,090
3DL	340,636,885	136,140	68,689	24,264	6.53	22,646
3DS	228,916,862	145,224	72,644	23,143	6.42	16,817
4AL	363,230,010	179,374	89,157	33,873	5.46	18,295
4AS	276,247,067	181,019	91,272	35,335	4.98	14,167
4BL	272,849,020	240,935	127,687	58,815	4.99	7,632
4BS	310,515,948	224,543	110,746	45,899	4.90	14,697
4DL	306,806,261	171,404	80,284	28,140	6.31	18,791
4DS	171,621,745	137,248	68,499	21,787	6.30	13,021
5AL	413,139,451	161,674	81,944	33,128	5.90	18,826
5AS	231,190,161	180,634	89,316	35,125	5.14	11,705
5BL	466,173,773	207,503	107,733	43,825	5.21	19,325
5BS	182,789,732	209,845	107,461	40,181	5.16	9,793
5DL	345,449,775	130,074	65,820	23,183	7.02	23,851
5DS	173,821,965	133,804	64,345	18,898	6.58	14,481
6AL	302,563,130	168,100	85,773	33,526	5.53	14,457
6AS	264,274,034	160,498	81,455	30,863	5.68	14,315
6BL	362,924,849	203,268	110,331	45,402	5.22	13,913
6BS	299,250,616	185,879	100,360	38,835	5.51	13,349
6DL	236,649,310	143,791	71,511	24,364	6.34	16,246
6DS	178,741,401	146,601	65,202	21,073	6.62	13,586
7AL	334,861,391	184,024	92,381	37,818	5.49	13,158
7AS	259,954,140	187,229	99,434	47,521	5.56	7,777
7BL	406,571,657	203,402	107,841	45,705	5.17	15,233
7BS	287,930,109	222,106	119,366	48,224	4.95	10,813
7DL	273,279,341	135,861	69,599	23,246	6.84	18,964
7DS	303,641,845	133,599	68,218	24,284	6.63	19,510
U	680,947,588	192,507	78,842	6,368	6.58	88,799
Total	13,427,354,022	180,094	88,778	32,825	5.73	735,943

4.8 Assembly validation

4.8.1 Contig validation by mate-pair link support

To validate the contigs produced by the w2rap-contigger, we used the Nextera 11kbp mate pair library as an independent dataset, before it was incorporated into the assembly during scaffolding. We used this library to find unsupported regions in the contigs, by assessing the link support.

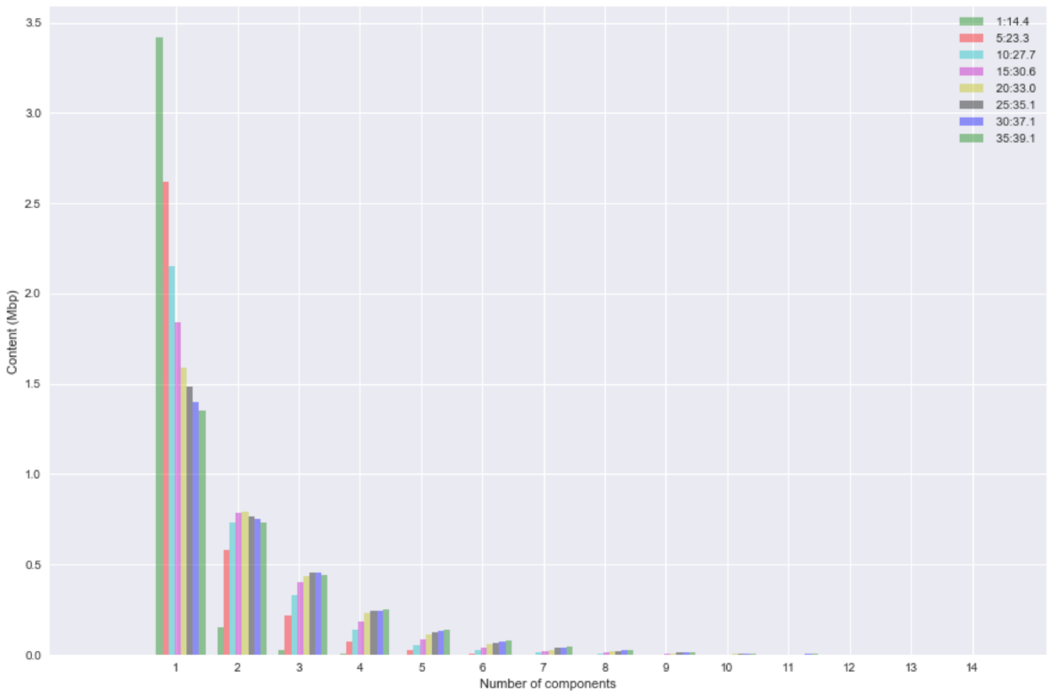

Figure S4.4: Content on contigs by number of components in the contig when splitting at breakpoints with support <percentile>:<link threshold>

The mate pair library was aligned using BWA to all contigs longer than 33kbp (3× the length of the library), the links were projected on each contig to obtain a measure of bridging link coverage. This reflected the amount of support at each position across the contig. Breakpoints were identified on any contig position with low link support; subsequently, the amount of sequence contained on contigs divided in different number of components and a corrected N50 for the set of broken contigs were computed. To choose link thresholds, a sample of 100 contigs was taken and the percentiles of the accumulated link distribution was computed (0, 0.1, 0.2, 0.3, 0.4, 0.5, 0.6, 0.7, 0.8, 0.9, 1, 5, 10, 15, 20, 25, 30, 35th percentile), ; this procedure was repeated 500 times and the mean of those percentile distributions was used for the breakpoint calculations. These analyses are shown in Figures S4.4 and S4.5.

Both plots show a very small amount of affected content and a very small change of N50 in the lower percentiles of coverage, with values of linkage that are much higher than the usual accepted thresholds of 3 to 5 links. At the 1st percentile, with more than 14 links, most of the content is on single-component contigs, with only a small amount of content on 2-component contigs and negligible content on contigs broken into more components. Higher percentiles are included to show how the assembly breaks down as expected once the requirement for links is higher than the typical coverage, but we do not consider any of those thresholds to represent significant risk of misassemblies.

The code for this analysis is available on `https://github.com/bioinfologics/assembly_validation/tree/master/link_support`.

4.8.2 Scaffold validation by gene order between 3B and TGACv1 3B sequences

As a proxy to assess the accuracy of the scaffold linkage, we used the alignment of 3B genes to contigs and scaffolds to assess the coherence between our assembly and the 3B pseudo-molecule reference. We looked for blocks on our contigs and scaffolds where two or more genes aligned and compared the order of genes in these blocks to gene order in the 3B pseudo-molecule. In all cases, on both contigs and scaffolds we found gene order in full agreement with 3B (Table S4.5).

This provides extra evidence that at least on the genic level, our assemblies are consistent with the existing reference, with the scaffolds generating precise linkage over longer ranges.

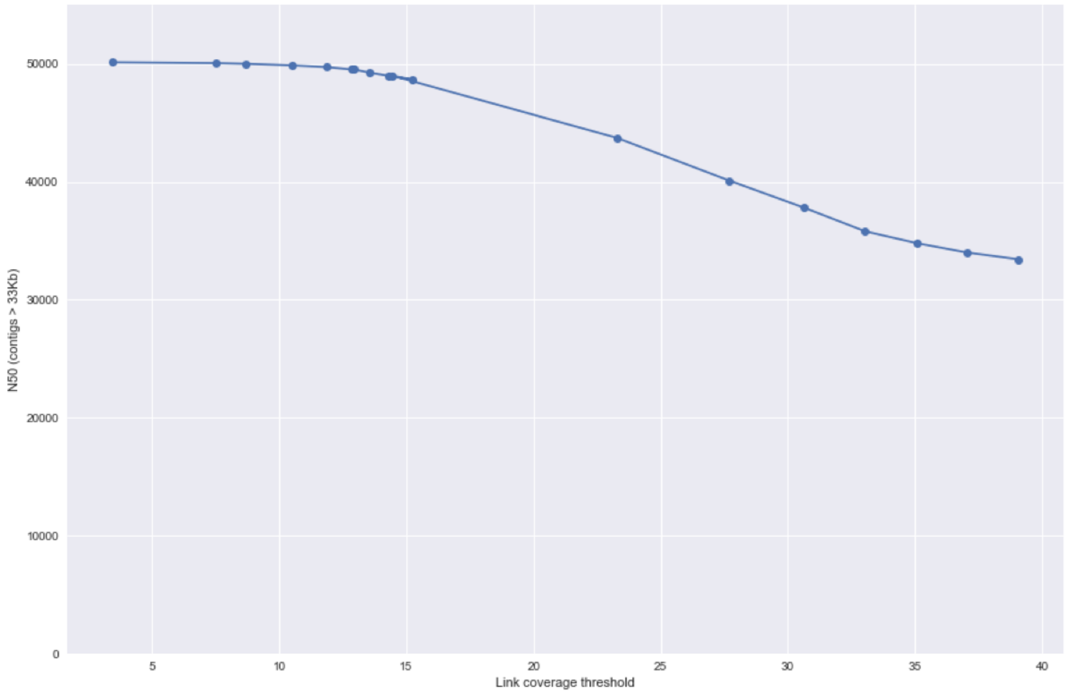

Figure S4.5: N50 for the contig subset when splitting at breakpoints by link coverage threshold.

Table S4.5: Identification of gene blocks.

	Number of blocks	Genes in syntenic blocks	Sequence contained in blocks (Mb)
TGACv1 contigs	1,266	3,224	15.0
TGACv1 scaffolds	1,503	4,792	60.7

4.9 Assessment of chromosome arm assignment accuracy

Table S4.6: Assessment of chromosome arm assignment accuracy.

	Genes aligned	Genes aligned to 3B classified scaffolds	Genes aligned to potential 3B scaffolds	Genes aligned to other arms
TGACv1 contigs	6,859	6,185	50	624
TGACv1 scaffolds	7,124	6,487	169	468

We aligned the genes identified on chromosome 3B (Choulet et al., 2014) to our assembled contigs and scaffolds in order to assess how accurately our algorithm assigned sequences to chromosome arms. We aligned these sequences with GMAP, using as a minimum threshold 95% alignment identity over 80% of the sequence length. We found that for contigs, 90.2% of genes aligned to 3B classified scaffolds with a further 0.7% aligning to potential 3B scaffolds (unclassified but with 3B as a suggested assignment). 9.1% aligned to other arms. For scaffolds, 91.1% aligned to 3B classified sequences, 2.4% to potential 3B scaffolds and 6.7% to other arms.

5 Integration with genetic maps and chromosomal alignments

To order the TGACv1 scaffolds along the wheat genome, we used the "Synthetic W7984" × Opata M85 map, hereafter WGS map, described in Chapman et al. (2015). The WGS map was constructed using a whole genome shotgun approach on 78 double haploid (DH) lines derived from W7984/Opata F1 hybrids. In order to anchor the TGACv1 scaffolds to the WGS map, we used the 437,973 scaffolds of the W7984 assembly, which were assigned to the genetic bins of the WGS map, as markers. Given the relatively low functional population size and high number of markers, even small frequencies of scoring error will result in high rates of ordering ambiguities between markers within short genetic distances. We corrected the genetic distances between bins by iterating over the bins b and merging bins b_i and b_{i+1} into b'_i if:

- $|b_i - b_{i+1}| < 1.6$ recombinations (1 recombination represents 0.586cM on the WGS map)
- b'_i did not span more than 2.5cM [Abraham Korol - pers. comm.]

The map position for each b'_i was calculated as the arithmetic mean of all bins merged into it. A mapping between the original WGS map bins and our corrected version can be found in (Supplementary File **S4**). Marker sequences were then aligned against all TGACv1 scaffolds with megablast (blast version 2.2.28, multithreaded). Only the best BLAST hit (`-max_target_seqs 1`) for each marker was taken into consideration. Markers that could be aligned equally well to more than one scaffold were discarded. BLAST hits were filtered by e-value (less than 10×10^{-10}), percent identity (at least more than 98.5%), and alignment length (at least 1kbp of the marker sequence is aligned).

TGACv1 scaffolds were then anchored to the corrected WGS map by assigning them to the genetic bin of their matching marker sequences. In order to deal with ambiguous bin assignments due to multiple markers matching a scaffold, we classified the anchored scaffolds according to the following scheme:

1. *unique*: all matching markers are assigned to the same bin.
2. *ambiguous*: matching markers are assigned to the same chromosome but to different genetic bins.
3. *homoeolog*: matching markers are assigned to the same chromosome of different subgenomes.
4. *conflict*: matching markers are assigned to different, non-homeologous chromosomes.
5. *novel*: subset of class1:unique comprising scaffolds that do not have a CSS-based chromosome arm assignment.
6. *cc_unique*: subset of *unique*, comprising scaffolds with conflicting CSS-based and genetic map-based chromosome assignments (cc: Chapman/Clavijo conflict).
7. *cc_ambiguous*: subset of *ambiguous*, comprising scaffolds with conflicting CSS-based and genetic map-based chromosome assignments.

The final TGACv1 map was constructed only from uniquely anchored scaffolds, i.e. scaffolds of classes 1, 5, and 6. The map is available in Supplementary File **S5** and scaffold classifications for all anchored scaffolds in Supplementary File **S6**. Python scripts for generation of the TGACv1 map are available at `https://github.com/krasileva-group/tgac-map`.

6 Detection and confirmation of chromosomal translocations

6.1 Detection of translocations from OrthoMCL output

Potential chromosome translocation events were identified as outlier triads of orthologous sequences (as identified by OrthoMCL, see Section 10). These triads are defined as three orthologous sequences that belong to the same OrthoMCL group, with two of the sequences being assigned to two different homoeologous chromosomes (e.g. 5B, 5D) and the third sequence, the "outlier", to a different non-homoeologous chromosome (e.g. 4A). The translocated sequence is assumed to have moved from the missing chromosome (source) of the homoeologous triplet (5A in the example case) to the chromosome on which the outlier sequence is located (destination).

In the present analysis, we further included orthologs with multiple copies on either of the three involved chromosomes. As chromosomal translocations typically do not involve just a single gene but a whole chromosomal region and such copies could have occurred independently of a translocation, these occurrences would not prevent either of the copies (or all) to be translocated.

6.2 PCR assays of suspected translocations

Triads with sequences that are annotated as being transposon-associated were ignored. In order to validate these potential translocation events via PCR, primer pair candidates for the outlier sequence were designed using Polymarker (Ramirez-Gonzalez et al., 2015) without specifying marker SNPs. The candidate pairs were then checked for specificity via `blastn` (using Blast 2.2.28, multithreaded with `-task blastn-short`, `-evalue 20`, `-dust no`). Primer pairs were discarded if any off-target Blast hit with up to 3 mismatches/indels was found.

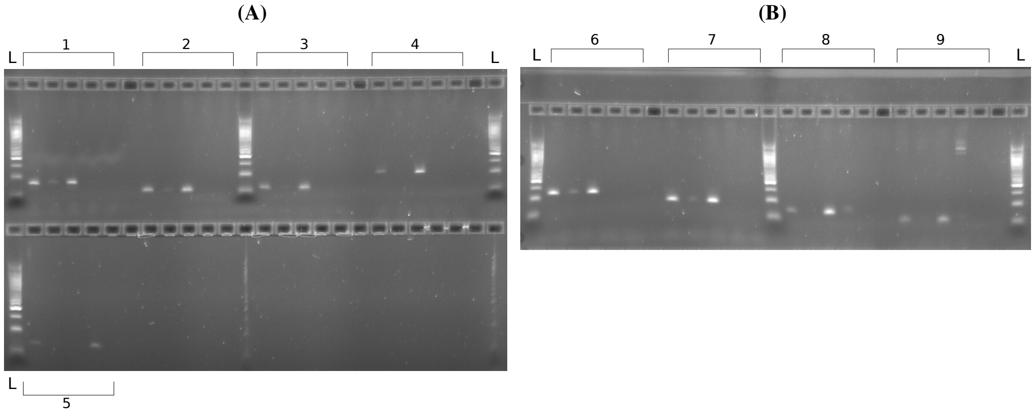

Figure S6.1: Gel images for gels 1–2 (panels **A**–**B**, respectively). Each gel contains five lanes per primer pair as described in the text. See Table S6.1 for details on each primer pair.

Primer pair	Gel	Lanes	Translocation type	Translocation group	Fragment length	Annealing temperature (°C)
1	1	1–5	4AL_5AL	group17899	78	55
2	1	7–11	4AL_5AL	group17588	57	55
3	1	13–17	7BS_4AL	group1175	58	55
4	1	19–23	5BL_4BS	group17187	101	55
5	1	25–29	7AL_3AL	group12953	67	55
6	2	1–5	4AL_5AL	group16803	118	60
7	2	7–11	5AL_4AL	group3295	89	60
8	2	13–17	7BS_4AL	group6850	69	60
9	2	19–23	5AL_7BS	group7391	50	60

Table S6.1: Primer pairs.

We tested a set of 9 genes corresponding to 3 known and 3 novel predicted translocations by PCR amplification of wild type and appropriate nullisomic lines (Sears, 1966). Five reactions were set up for each primer pair using the following template genomic DNA:

- 10ng Chinese Spring wheat
- 1ng Chinese Spring wheat
- 10ng nullisomic gDNA for the predicted source chromosome
- 10ng nullisomic gDNA for the destination chromosome predicted to receive the locus

- a negative control without DNA.

A reaction volume of 25μL was used with the following final concentrations:

- 1× Flexi Buffer
- 2mM $MgCl_2$
- 0.2mM dNTP each
- 0.5μM forward primer
- 0.5μM reverse primer
- 0.025U/μL of GoTaq Hot Start Polymerase

PCR was performed using a AB Verity with the following programme:

- 2min at 95°C
- 32 cycles of:
  - 30s at 95°C
  - 30s at 55°C
  - 10s at 72°C
- A final extension for 30s at 72°C

For primer pairs where non-specific bands were observed, the annealing temperature was increased from 55°C to 60°C in order to improve the stringency/specificity. We ran 10μL of the amplicons on 4% agarose E-gels (Invitrogen) and scored PCRs that amplified the Chinese Spring control cleanly. As primers could produce some off-target amplifications (e.g. homoeologous copies) we scored departures and arrival nullisomics as negative if they produced a band at the same intensity as 1ng of Chinese Spring or lower, bands of the same intensity were scored as ambiguous. Details on all the primers and the experiments are reported in Supplementary File **S7**.

6.3 Cross-validation of translocations by using the genetic map

Overall 436 (35%) of all 1240 triads (supporting 152, or 40.75%, of 373 potential translocation events) could be anchored to the TGACv1 map (Supplementary File: **S3**). Of these, 416 (33.55%) triads (supporting 146 — 11.77% — potential translocation events) could be anchored without conflict between their CSS-based chromosome assignment and their genetic bin on the TGACv1 map. In 8 out of 20 conflicting triads the chromosome on the TGACv1 map is identical to the source chromosome of the potential translocation event (Table S6.2), rendering the event undetectable when relying solely on TGACv1 map information.

Table S6.2: Triads with conflicts between TGACv1 map and CSS chromosome arm assignment

Gene/Representative transcript	OrthoMCL group	Translocation		TGACv1 genetic bin	Note
		Source	Destination		
TRIAE_CS42_5DS_TGACv1_457137_AA1482860.1	group11550	2DL	5DS	2D:65.70	map chromosome is source chromosome
TRIAE_CS42_7BL_TGACv1_576879_AA1858370.1	group14472	3B	7BL	3B:45.71	map chromosome is source chromosome
TRIAE_CS42_7BL_TGACv1_576879_AA1858380.1	group14473	3B	7BL	3B:45.71	map chromosome is source chromosome
TRIAE_CS42_7BL_TGACv1_576879_AA1858390.1	group14474	3B	7BL	3B:45.71	map chromosome is source chromosome
TRIAE_CS42_5DL_TGACv1_436092_AA1457960.1	group15184	5AL	4AL	3A:49.69	
TRIAE_CS42_5DL_TGACv1_435790_AA1454970.1	group15512	5AL	4AL	5B:129.99	
TRIAE_CS42_5DL_TGACv1_436307_AA1459890.1	group15869	5AL	4AL	4A:106.06	
TRIAE_CS42_4DL_TGACv1_342399_AA1112520.1	group17975	4AL	5AL	6B:70.29	
TRIAE_CS42_4DL_TGACv1_342399_AA1112540.1	group17976	4AL	5AL	6B:70.29	
TRIAE_CS42_4DL_TGACv1_342399_AA1112570.1	group17977	4AL	5AL	6B:70.29	
TRIAE_CS42_6AS_TGACv1_485809_AA1552570.1	group20423	2AL	6AS	2A:82.29	map chromosome is source chromosome
TRIAE_CS42_5DL_TGACv1_433289_AA1408400.1	group22982	5AL	2AL	7B:51.03	
TRIAE_CS42_5DL_TGACv1_436092_AA1457970.1	group23416	5AL	4AL	3A:49.69	
TRIAE_CS42_5AS_TGACv1_392779_AA1264470.1	group23830	7AL	5AS	7A:63.00	map chromosome is source chromosome
TRIAE_CS42_2AS_TGACv1_112471_AA0338700.1	group3312	2BS	5BL	7A:68.56	
TRIAE_CS42_4DL_TGACv1_342436_AA1113710.2	group4786	4AL	5AL	7D:73.08	
TRIAE_CS42_2BS_TGACv1_146689_AA0470800.1	group5765	2DS	3DL	3B:48.27	
TRIAE_CS42_5AS_TGACv1_393155_AA1269210.1	group6137	3AL	5AS	3A:70.72	map chromosome is source chromosome
TRIAE_CS42_1BS_TGACv1_050024_AA0166120.1	group7241	3AL	1BS	3A:57.65	map chromosome is source chromosome
TRIAE_CS42_7DL_TGACv1_605399_AA2006440.1	group7349	7AL	4BL	4D:47.93	

7 Repeat analysis

Transposons were detected and classified by a homology search against the REdat_9.7_Triticeae section (13,229 elements, 100Mbp) from the PGSB transposon library (Spannagl et al., 2016). The program vmatch (`http://www.vmatch.de`) was used for that purpose as a fast and efficient matching tool suited for large and highly repetitive genomes with the following parameters: identity greater or equal to 70%, minimal hit length 75bp, seedlength 12bp; the exact commandline is:

```
-d -p -l 75 -identity 70 -seedlength 12 -exdrop 5
```

The vmatch output was filtered for redundant hits via a priority based approach, which assigns higher scoring matches first and either shortens (less than 90% coverage and at least 50bp rest length) or removes lower scoring overlaps to obtain an overlap free annotation.

Full-length LTR-retrotransposons elements were identified with LTRharvest (Ellinghaus et al., 2008), which reported 354,315 non overlapping candidate sequences under the following parameter settings:

```
overlaps best -seed 30 -minlenltr 100 -maxlenltr 2000 -mindistltr 3000
-maxdistltr 25000 -similar 85 -mintsd 4 -maxtsd 20 -motif tgca
-motifmis 1 -vic 60 -xdrop 5 -mat 2 -mis -2 -ins -3 -del -3
```

All candidates where annotated for PfamA domains with hmmer3 (Eddy, 2011) and stringently filtered for false positives by several criteria, the main ones being the presence of at least one typical retrotransposon domain (e.g. RT, RH, INT, GAG) and a tandem repeat content below 25%. The filtering steps led to a final set of 44,579 high confidence full-length LTR retrotransposons. The composition of repeats in the assembled genome can be observed in Table S7.1.

	% of Genome	% of TE	Number	Total (Mb)	Average length (bp)
**Mobile Element (TXX)**	81.10	100.00	9,673,829	10,268.4	1061
**Class I: Retroelement (RXX)**	67.70	83.50	7,249,022	8571.7	1182
LTR Retrotransposon (RLX)	67.30	83.00	7,171,177	8522.1	1188
Ty1/copia (RLC)	14.20	17.50	1,555,328	1792.5	1152
Ty3/gypsy (RLG)	30.80	37.90	2,971,111	3895.2	1311
Unclassified LTR (RLX)	20.80	25.60	2,621,553	2627.7	1002
non-LTR Retrotransposon (RXX)	0.40	0.50	77,845	49.6	638
LINE (RIX)	0.40	0.50	72,414	47.6	657
SINE (RSX)	0.00	0.00	5431	2.2	375
**Class II: DNA Transposon (DXX)**	12.90	15.90	2,233,197	1636.3	733
DNA Transposon Superfamily (DTX)	12.80	15.70	2,123,788	1616.6	761
CACTA superfamily (DTC)	12.40	15.30	1,951,401	1567.8	803
hAT superfamily (DTA)	0.01	0.01	1642	0.6	393
Mutator superfamily (DTM)	0.16	0.20	61,612	20.3	329
Tc1/Mariner superfamily (DTT)	0.04	0.05	37,550	5.0	134
PIF/Harbinger (DTH)	0.12	0.15	34,127	15.1	443
unclassified (DTX)	0.06	0.07	37,456	7.7	206
DNA Transposon Derivative (DXX)	0.13	0.16	102,275	16.5	162
MITE (DXX)	0.13	0.16	102,275	16.5	162
Helitron (DHH)	0.01	0.01	1965	1.5	765
unclassified DNA transposon (DXX)	0.01	0.02	5169	1.7	331
Unclassified Element (TXX)	0.48	0.59	191,610	60.3	315
**Retro-TE/DNA-TE ratio**	5.20				
**Gypsy/Copia ratio**	2.20				

Table S7.1: Repeat composition of the bread wheat genome.

8 Construction of the wheat gene set

The wheat gene set for wheat was generated using a custom pipeline integrating wheat-specific transcriptomic resources, including PacBio transcriptomic data, similarity to proteins of related species, and evidence-guided ab initio predictions generated with AUGUSTUS (Stanke et al., 2006).

The pipeline was divided in five different phases. In the first phase, RNA-Seq models were generated with 4 different assembly methods utilising data from multiple tissues and conditions, and integrated together with PacBio transcripts into a coherent and non-redundant set of models using Mikado (Venturini et al., 2016). In the second phase, PacBio reads were classified based on protein similarity and a subset of high quality (e.g. full length, canonical splicing, non-redundant) transcripts employed to train an AUGUSTUS wheat-specific gene prediction model. In the third phase, AUGUSTUS was used to generate a first draft of the genome annotation, using as input Mikado-filtered transcript models, reliable junctions identified with Portcullis (Mapleson et al., 2016), and peptide alignments of proteins from five different species closely related to wheat (*Brachypodium distachyon* 314 v. 3.1, *Zea mais* 284 v. 6a, *Oryza sativa* 204 v. 7.0, *Sorghum bicolor* 313 v. 3.1, and *Setaria italica* 312 v. 2.2, all downloaded from Phytozome (Goodstein et al., 2012)). In the fourth stage, this draft annotation was refined and polished by identifying and correcting probable gene fusions, missing loci and alternative splice variants. Finally, the polished annotation was functionally annotated and all loci were assigned a confidence rank based on their similarity to known proteins and their agreement with wheat transcriptomic data.

8.1 Reference guided transcriptome reconstruction

8.1.1 Alignment of Illumina RNA-seq data

Data preparation RNA-Seq data from three different datasets was utilised for the annotation: ERP004714 (used for the annotation provided in The International Wheat Genome Sequencing Consortium (2014)), ERP004505 (used for the grain-development analyses in Pfeifer et al. (2014)) and an internally generated dataset of 250bp paired-end strand-specific reads from six different tissues (PRJEB15048; Table S8.1). In total, the three datasets comprised over 3.2 billion paired-end reads. For each dataset, read samples were collapsed by tissue and filtered using trim-galore v. 0.3.7 (BabrahamLab, 2014), with the command line options:

`-q 20 --phred33 --stringency 5 --fastqc --length 60`

Due to concerns of high concentration of ribosomal RNA in the internally produced samples, reads from that dataset were further filtered using SortMeRNA v. 2.0 (Kopylova et al., 2012), with the command line options:

`--num_alignments 1 --fastx --paired_in`

and using RFam (5S and 5.8S) and Silva (Archea 16S-23S, Bacteria 16S-23S, Eukariota 18S-28S) as databases.

Alignment with STAR Filtered reads were aligned to the wheat genome using a forked version of STAR-2.5.0-alpha (Dobin et al. (2013), commit `f82c5a0028`; see (`https://github.com/alexdobin/STAR/issues/85`)). The genome was indexed using the option

`--genomeChrBinNbits 14`

in accordance with STAR documentation, and the process had to be performed on a UV supercomputer due to the memory requirements (∼2TB of RAM). Reads were aligned with stringent parameter in a two pass approach to ensure alignment accuracy, a first pass using the custom command-line options

```
--outFilterMismatchNmax 3 --alignEndsType EndToEnd
--alignIntronMin 20 --alignIntronMax 200000
--outSJfilterIntronMaxVsReadN 10000 10000 10000
```

to increase the accuracy of the alignments and

`--outSAMattributes NH HI NM MD AS XS`

to ensure the compatibility of the output with downstream tools such as Cufflinks (Trapnell et al., 2010). All 1,519,861reliable junctions detected by STAR in at least one sample during this first pass were collapsed, and given as input for a second round of alignments, with the same command line parameters but also providing the merged junction file with the options:

`--limitSjdbInsertNsj 2000000 --sjdbOverhang 250`

Finally, the alignments from all samples were filtered with portcullis v. 0.10.1 (Mapleson et al., 2016) to exclude spliced reads with non-canonical junctions that were on manual review identified as predominantly due to misalignment.

Alignment with TopHat2 As the original IWGSC annotation had been created using the aligner TopHat2 (Kim et al., 2013), we also aligned reads from the ERP004714 dataset using this program. To retrieve splicing junctions related to the original annotation, IWGSC models were aligned against our reference using GMAP v. 2015-09-29 (Wu and Watanabe, 2005), with the command line options:

`--min-identity=0.99 --min-trimmed-coverage=0.90 -n 1`

and subsequently collapsed and filtered for models only with canonical junctions using gffread from Cufflinks v. 2.2.2beta (Trapnell et al., 2012; Roberts et al., 2011a,b). 281,562 unique splicing junctions from the aligned models were retrieved with a custom Python3 script from the surviving 85,242 models and provided to TopHat v.2.1.0 (patched to use Bowtie2.2.5 (Langmead and Salzberg, 2012) long indices; the patch was subsequently integrated into the later TopHat v.2.1.1). Reads from ERP004714 were then aligned in single pass using the CLI options

```
-a 13 -i 20 -I 400000 -g 20 --no-discordant -N 1 --read-edit-dist 1 --read-realign-edit-dist 1 --read-gap-length
1 --library-type fr-unstranded
```

and additionally providing the junction file from above.

Table S8.1: Sequencing reads used in this study. ERP004714: Grain, Leaf, Root, Spike and Stem, ERP004505: 10DPA, AL_20DPA, AL.SE_30DPA, REF_20DPA, SE_20DPA, SE_30DPA and TC_20DPA, PRJEB15048: seedling, root, leaf, stem, spike and seed.

	ERP004714	ERP004505	PRJEB15048
Number of samples	5	7	6
Number of reads	1,536,051,415	873,709,556	824,241,135
Number of filtered reads	1,412,029,174	873,550,049	731,931,657
Average no. filtered reads per sample	282,405,834.8	124,792,864.1	121,988,609.5
Aligned reads (STAR)	1,203,100,456	744,087,908	488,750,691
Aligned reads (STAR second pass)	1,267,816,403	759,278,032	579,642,183
Aligned reads (TopHat2)	1,299,830,440	NA	NA

Table S8.2: Number of PacBio reads, per sample and size-fraction.

Stage	Size Fraction	Leaf	Root	Seed	Seedling	Spike	Stem	Total
Reads of insert	0.7 - 2 kbps	345,566	482,417	410,969	227253	353,196	210,462	2,029,863
	2-3 kbps	267,379	410,186	364,988	330,525	375,062	376,717	2,124,857
	3-5 kbps	367,571	356,396	301,030	110,628	311,537	370,739	1,817,901
	Total	980,516	1,248,999	1,076,987	668,406	1,039,795	957,918	5,972,621
IsoSeq + Quiver	0.7 - 2 kbps	69,817	116,164	86,031	77,211	98,848	79,909	527,980
	2-3 kbps	55,789	125,622	77,619	97,894	90,340	104,293	551,557
	3-5 kbps	73,513	73,351	56,315	34,818	88,516	103,272	429,785
	Total	199,119	315,137	219,965	209,923	277,704	287,474	1,509,322
Aligned		187,583	297,970	205,990	197,535	259,329	265,816	1,414,223
% aligned		94.21%	94.55%	93.65%	94.10%	93.38%	92.47%	93.70%

8.1.2 Alignment of PacBio RNA-seq data

Data preparation PacBio sequencing data from six tissues was analysed initially using the SMRTanalysis package (v2.3.0.140936), stopping at the quiver step. The "CircularConsensus" step of the ConsensusTools utility was called with the command-line options
`--minFullPasses 0 --minPredictedAccuracy 75`
while during the classification step the option
`--min_seq_len 300`
was invoked. The pipeline provided a total of over 1.5 million PacBio transcriptomic reads for downstream analyses (Table S8.2).

Read alignment PacBio reads were aligned using the gmapl utility from GMAP v. 2015-11-20 (Wu and Watanabe, 2005), with the command line options
`-f 2 --no-chimera -n 1 --min-trimmed-coverage=0.90 --min-identity=0.95 --split-output`
We further discarded alignments deemed to be translocations by GMAP (those reported in the .transloc file).

8.1.3 Transcript assembly

The illumina RNA-Seq alignments (18 from STAR and 5 from TopHat2) were assembled by tissue/condition using three different tools: CLASS v. 2.12 (Song et al., 2016), Cufflinks v. 2.2.2 beta (commit 753c109e31; Trapnell et al. (2010); Roberts et al. (2011a,b)) and StringTie v.1 .10 (Pertea et al., 2015). CLASS was called using the option `-F 0.05`; Cufflinks was invoked asking to limit the intron size to 200,000 and using both the fragment-bias correction and the multi-read rescue method:
`-I [200000] -b -u`
Samples from the internal dataset were assembled using also the option:

Table S8.3: Illumina and PacBio transcript assembly statistics. For each tool, assembled transcripts have been clustered into loci using `cuffcompare` (v.2.2.1, command line options "`-C -G`"; Trapnell et al. (2010))

Method	Loci	Transcripts	Average number of exons	Average cDNA size	Number of monoexonic transcripts
CLASS	181,259	3,188,679	5.48	1,304.55	326,210
Cufflinks	270,456	3,281,661	4.37	1,595.44	1,078,721
StringTie	285,728	3,826,431	4.47	1,554.83	1,117,717
Trinity	244,384	646,244	2.96	1,301.02	333,428
PacBio (4 samples)	81,752	1,020,650	6.80	2,109.06	131,357
PacBio (all 6 samples)	88,609	1,330,372	6.79	2,100.97	173,661

Table S8.4: Mikado transcript assembly statistics.

	Genes	Transcripts	Average number of exons	Average cDNA size	Number of monoexonic transcripts
Mikado (4 PacBio)	81,848	120,886	6.36	2,098.83	18,554
Mikado (6 PacBio)	83,144	128,030	6.29	2,182.37	19,175
Mikado (Illumina and PacBio)	273,243	373,861	4.07	1,377.70	93,564

`--library-type fr-firststrand`
StringTie was invoked asking for assemblies longer than 200bp ("`-m 200`"). In addition the alignments of reads from the internal dataset (6 tissues) were merged using the MergeSamFiles utility from picard (Wysokar et al., 2016). The merged BAM file was used as input for Trinity v.2.1.1 (Haas et al., 2013) in genome-guided mode, using the command line options:
`--SS_lib_type RF --genome_guided_max_intron 200000`
The assembled transcripts were then aligned against the genome using gmap from GMAP v. 2015-11-20 (Wu and Watanabe, 2005), using the command line options:
`-f 2 --min-trimmed-coverage=0.80 --min-identity=0.90`
Uniquely and multiply mapping transcripts were further filtered using a custom python3 script to retain only those alignments in which the assembled transcript mapped against the same region from which its original read cluster originated from. The number and features of transcripts detected by each method is reported in Table S8.3.

We used Mikado (Venturini et al., 2016) to integrate the ~11 million Illumina assemblies generated by multiple assembly tools (CLASS, Cufflinks, StringTie, Trinity) and ~1.4 million aligned PacBio reads. Mikado leverages transcript assemblies generated by multiple methods to improve transcript reconstruction. Loci are first defined across all input assemblies with each assembled transcript scored based on metrics relating to ORF and cDNA size, relative position of the ORF within the transcript, UTR length and presence of multiple ORFs. The best scoring transcript assembly is then returned along with additional transcripts (splice variants) compatible with the representative transcript.

We generated three Mikado selected transcript sets for use in gene predictor training or annotation (Table S8.4):

1. Alignments from 4 PacBio samples (Root, Seedling, Spike, Stem) were analysed with Mikado 0.11.0, without BLAST data and disabling the "chimera_split" algorithm. The transcript set was used in gene predictor training.
2. Mikado (v. 0.19.2) run on the full set of 6 PacBio samples, with BLAST data, and enabling the chimera_split option in "PERMISSIVE" mode.
3. The 70 RNA-Seq assemblies (23 alignments * 3 assemblers + Trinity) and PacBio alignments (Root, Seedling, Spike, Stem) were analysed using Mikado v. 0.18.0 with the "chimera_split" option set to PERMISSIVE.

For Mikado runs incorporating BLAST data transcripts passing the "prepare" step were blasted against filtered and masked proteins of *B. distachyon*, *O. sativa*, *S. bicolor*, *S. italica* and *Z. mays* using BLAST+ v. 2.2.30 and limiting each result to the best 15 matches.

8.2 Gene predictor training

The primary PacBio alignments from 4 samples (Root, Seedling, Spike, Stem) analysed with Mikado 0.11.0 were filtered for full-length complete and coding transcripts using Full-lengtherNEXT (v0.0.8; Fernandez and Guerrero (2012)) with open reading frames (ORFs) predicted using TransDecoder v2.0.1 (Grabherr et al., 2011). A reliable set of transcripts were selected for training AUGUSTUS having single full length ORF, with 5' and 3' UTR present, consistent Full-lengtherNEXT and TransDecoder CDS coordinates, a minimum CDS to transcript ratio of 50% and a single transcript per gene. We excluded genes with a genomic overlap within 1000bp of a second gene and gene models that are homologous to each other with a coverage and identify of 80%. The filtered PacBio set contained 9952 transcripts selected for training AUGUSTUS. The trained AUGUSTUS model resulted in 0.941 sn, 0.844 sp nucleotide level, 0.798 sn, 0.756 sp exon level and 0.455 sn, 0.367 sp at the gene level.

8.3 Gene prediction using evidence guided AUGUSTUS

Protein coding genes were predicted using AUGUSTUS (Stanke et al., 2006) by means of a Generalized Hidden Markov Model (GHMM) that takes both intrinsic and extrinsic information into account.

8.3.1 Generation of external hints for gene prediction

Junctions RNA-Seq junctions (defining introns) were derived from RNA-Seq alignments (From TGAC: Leaf, Stem, Spike, Seed, Seedling and Root samples; From accession ERP004505: 10DPA, AL_20DPA, AL.SE_30DPA, REF_20DPA, SE_20DPA, SE_30DPA and TC_20DPA samples; From accession ERP004714: Grain, Leaf, Root, Spike and Stem samples), using portcullis v.0.12.0 (Mapleson et al., 2016) and the default set of filtering parameters. Junctions that pass and fail the portcullis filter were classified as Gold and Silver respectively.

Table S8.5: Description of reference protein datasets used with AUGUSTUS (Stanke et al., 2006). Proteins were filtered at 50% identity and 80% coverage and junctions checked against the Illumina junctions as an additional filtering criterion. Any intron over 50kb resulted in the protein alignment being removed.

	B. distachyon	***O. sativa***	***S. bicolor***	***S. italica***	***Z. mays***
Total Proteins	52,972	49,061	47,205	43,001	88,760
Proteins Aligned	30,354	23,929	23,231	23,107	38.653
Proteins Aligned (%)	57.30%	48.77%	49.21%	53.74%	43.55%
Protein Alignments	105,190	89,739	83,561	86,381	142,217

Proteins Protein sequences from 5 species (*Brachypodium distachyon*, *Oryza sativa*, *Sorghum bicolor*, *Setaria italica* and *Zea mays*) were soft masked for low complexity (segmasker from NCBI BLAST+ 2.3.0) and aligned to the soft masked genome (using PGSB repeats) with exonerate v2.2.0 (Slater and Birney, 2005) with parameters:
`--model protein2genome --softmaskquery yes --softmasktarget yes --bestn 10 --minintron 20`

To identify a high confidence set of alignments, exonerate results were filtered at 50% identity and 80% coverage. Furthermore, alignments whose introns were either longer than 50kbps or that were not present in the set of Illumina RNA-Seq junctions were removed from further analysis (see Table S8.5).

PacBio transcript classification To generate high confidence evidence hints for gene prediction, Mikado filtered PacBio transcripts (Root, Seedling, Spike, Stem) were classified into the following three categories:

Gold : PacBio reads having a full length hit (complete/putative complete) with Full-LengtherNEXT and having a maximum of 2 complete 5'UTR exons and 1 complete 3'UTR exon;

Silver : Remaining models meeting the maximum 5'UTR and 3'UTR restrictions with an additional constraint of having at least 900bp CDS length;

Bronze : any remaining Mikado PacBio transcripts were assigned to the bronze category.

In addition, polished (Quiver high and low quality filtered) PacBio reads were filtered for splice sites that are concordant with Illumina RNA-Seq alignments and were used along with other evidences for the gene prediction.

Classification of Mikado transcripts The Mikado models (combining Illumina and PacBio assemblies) were classified into the following three categories:

Gold : Mikado transcripts having a full length hit (complete/putative complete) with Full-LengtherNEXT and having having a maximum of 2 complete 5'UTR exons and 1 complete 3'UTR exon;

Silver : Remaining models meeting UTR restrictions with an additional constraint of having at least 300bp CDS length;

Bronze : Any remaining Mikado transcripts were assigned to bronze category if they had a maximum intron length of 50kbp.

RNA-seq coverage hints Individual RNA-Seq bam files from STAR were merged together and reads were extracted from merged bam using picardtools (SamToFastq.jar v1.84; Wysokar et al. (2016)). The extracted PE reads were then normalised using a Trinity utility (v2.0.2; Grabherr et al. (2011)):
`insilico_read_normalization.pl --max\_cov 50 --pairs_together --KMER_SIZE 25`
and were used to create the normalised bam with picardtools (FilterSamReads.jar v1.84; Wysokar et al. (2016)). The wig file was generated using RSeQC v2.3.7 (bam2wig.py; Wang et al. (2012)) and then converted to a hints file using a utility provided with AUGUSTUS (v2.7; (Stanke et al., 2006)):
`wig2hints.pl --width=10 --margin=10 --minthresh=2 --minscore=4 --prune=0.1 --radius=4.5`

8.3.2 Gene prediction

AUGUSTUS (v2.7) was used to predict gene models for the Wheat CS42 TGACv1 genome assembly by utilising the evidence hints generated from five sets of cross species protein alignments, PacBio models, Mikado PacBio models, PacBio plus Illumina Mikado models and RNA-Seq junctions (defining introns). Interspersed repeats were provided as "nonexonpart" hints and RNA-Seq read density was provided as "exonpart" hints. We assigned higher bonus scores and priority based on evidence type and classification (Gold, Silver, Bronze) to reflect the reliability of different evidence sets (see supplementary AUGUSTUS config file **S8**); Statistics of the generated models are presented in Table S8.6).

Table S8.6: AUGUSTUS gene prediction statistics.

Gene Count	224,994
Total transcripts	224,994
Transcripts per gene	1
Transcript mean size (incl. intron) (bp)	3547.89
Transcript mean size cDNA (bp)	1447.66
Transcript median size cDNA (bp)	1239
Min cDNA	8
Max cDNA	15,613
Total exons	833,929
Exons per transcript	3.71
Exon mean size (bp)	390.58
Total exons (distinct)	827,714
Exon mean size (distinct) (bp)	392.09
CDS mean size (bp)	302.18
CDS mean size (distinct) (bp)	302.22
Transcript mean size CDS (bp)	959.71
Transcript median size CDS (bp)	747
Min CDS	3
Max CDS	14,259
5UTR mean size (bp)	154.03
5UTR mean size (distinct) (bp)	153.96
3UTR mean size (bp)	249.69
3UTR mean size (distinct) (bp)	249.73

8.4 Gene model refinement

The primary gene models generated by AUGUSTUS were corrected to remove long terminal introns spanning over 10kbp, identified from manual review as likely artefacts. To identify incorrectly split genes, AUGUSTUS gene models were compared against the high quality Mikado PacBio Gold and Silver set of gene models to identify cases where more than one AUGUSTUS model was contained within a PacBio model with at least 80% nucleotide precision (specificity), in which case we retained only the AUGUSTUS gene model with the highest nucleotide F1.

To add reliable alternative splice variants we ran PASA (Haas, 2003) with a filtered set of transcripts, removing from Mikado transcripts and PacBio reads those which had introns greater than 10kb, and retaining PacBio splice junctions that were consistent with RNA-Seq Illumina alignments. Transcripts were integrated into the annotation via a PASA utility:

```
validate_alignments_in_db.dB --MIN_INTRON_LENGTH=20 --MAX_INTRON_LENGTH=50000
 --MIN_PERCENT_ALIGNED=70 --MIN_AVG_PER_ID=95 --NUM_BP_PERFECT_SPLICE_BOUNDARY=3
```

A second round of updates to the annotation was generated with PASA assemblies constructed from only PacBio reads. To identify and correct gene annotation artefacts, any incorrectly fused PASA models were replaced with a PacBio Gold gene model when the latter was found to overlap with a nucleotide recall of at least 30%. PASA transcripts associated with the incorrectly fused PASA gene but not found to overlap with the PacBio Gold gene model were clustered into new loci and retained. Transcript models with cDNAs shorter than 300bp were removed from further analysis.

8.5 Assignment of gene biotypes and confidence classification

Gene models were classified as coding, non-coding and repeat associated and assigned as high or low confidence based on support from cross species protein similarity and wheat transcripts.

We decided to assign a confidence ranking to each transcript, in three levels:

Protein ranking : this rank is based on similarity - or lack thereof - of the transcript against publicly available protein datasets. The rankings go from 1 (best) to 5 (worst).

Transcript ranking: this rank is based on support for the model - or lack thereof - from our multiple sources of transcriptomic evidence. The rankings go from 1 (best) to 5 (worst).

Confidence: we assigned a general binary confidence tag ("High" vs "Low") for each transcript. To qualify to be considered a high-confidence *coding* transcript, a model has to fall in one of the following categories:

- Protein ranking P1 and transcript ranking T4 or better
- Protein ranking P2 and transcript ranking T4 or better
- Protein ranking P3 and transcript ranking T1

8.5.1 Cross species protein similarity ranking

Each gene model was assigned a protein rank (P1–P5) reflecting the level of coverage of the best identified homolog in a plant protein database. Protein ranks were assigned as:

Protein Rank 1 (P1) : proteins identified as full length in Full-LengtherNEXT with the UniProt database or at least 80% coverage in a supplementary BLAST database consisting of *A.thaliana*, *B. distachyon*, *O. Sativa*, *S. bicolor*, *S. italica* and *Z. mays* proteins

Protein Rank 2 (P2) : proteins with at least 60–80% coverage in the supplementary BLAST database;

Protein Rank 3 (P3) : proteins with at least 30–50% coverage in the supplementary BLAST database;

Protein Rank 4 (P4) : proteins with a low coverage hit (between 0–30%) in the supplementary BLAST database;

Protein Rank 5 (P5) : proteins with no hit in the supplementary BLAST database.

8.5.2 Wheat transcript support ranking

A transcript rank (T1–T5) was assigned based on the extent of support for the predicted gene model from either wheat PacBio reads or assembled wheat RNA-Seq data (all 10,943,015 transcripts assembled from all four transcript assembly methods).

We calculated a variant of annotation edit distance (AED) and used this to determine a transcript level ranking. First we define accuracy AC as:

$$AC = (SN + SP)/2$$

where SN is sensitivity and SP specificity, and then derived the AED:

$$AED = 1 - AC.$$

Rather than taking the union of all transcript evidence, we calculate AED at base, exon and splice junction level against all individual wheat transcripts used in our gene build (Illumina assemblies, cDNAs and PacBio reads), we then take the mean of base, exon and junction AED based on the transcript that best supported the gene model. AED statistics were calculated using the compare utility from Mikado (Venturini et al., 2016).

Transcript ranking was assigned based on:

Transcript Rank 1 (T1) : Full length support from cDNA or Pacbio read;

Transcript Rank 2 (T2) : full length support from Illumina assemblies;

Transcript Rank 3 (T3) : Best average AED less than 0.5;

Transcript Rank 4 (T4) : Best average AED between 0.5 and 1;

Transcript Rank 4 (T5) : No transcriptomic support (best average $AED = 1$).

8.5.3 Assignment of a locus biotype

Following the assignment of protein and transcript rankings, we assigned a locus biotype to each gene.

Repeat associated biotypes Genes were classified as repeat associated if all their transcripts aligned with at least 20% similarity and 30% coverage to the TransposonPSI library (v08222010; Haas (2010)) and had at least 40% coverage by PGSB interspersed repeats. In addition, genes with transcripts that had at least 20% similarity and 50% coverage to the TransposonPSI library or had at least 60% coverage by the PGSB interspersed repeats were also classified as repeat associated. In order to reduce the number of false positive calls, the combined set of putative repetitive transcripts identified above were further checked using a BLAST dataset (comprising protein sequences from *A. thaliana* TAIR10.31, *B. distachyon* v3.1, *H. vulgare* v1.31, *O. sativa* v7.0, *S. bicolor* v3.1, *S. italica* v2.2 and *Z. mays* v6a, all from Phytozome) filtered specifically for repeats, by excluding any sequence corresponding to one of the following parameters:

- Protein with a match for “retrotransposon”, “transposon” or both in their description
- At least 30% similarity and 60% coverage to a hit in TransposonPSI

Any assignment of repeat-associated status was judged a false positive call if the protein had a hit with at least 30% coverage against the filtered protein dataset above.

Non-coding RNAs Genes where all the transcript had a protein rank of P4 or P5 were checked to verify whether they could constitute putative non-coding RNAs. Transcript sequences were analysed with CPC v. 0.9.2 (Kong et al., 2007) in conjunction with Uniref90 from Uniprot (retrieved on 11th March 2016). Transcripts were called as putative non-coding RNAs if they met the following conditions:

- PR4 and CPC score lower or equal than -1
- PR5 and CPC score lower than 0

Table S8.7: Rankings and confidence of coding transcripts.

Protein Rank	Transcript Rank	Confidence	Transcript Count
P1	T1	High	66404
P1	T2	High	43423
P1	T3	High	20937
P1	T4	High	10013
P1	T5	Low	21469
P2	T1	High	3461
P2	T2	High	3545
P2	T3	High	3392
P2	T4	High	2084
P2	T5	Low	6213
P3	T1	High	1813
P3	T2	Low	4521
P3	T3	Low	3995
P3	T4	Low	3406
P3	T5	Low	12210
P4	T1	Low	781
P4	T2	Low	3116
P4	T3	Low	2846
P4	T4	Low	2494
P4	T5	Low	7484
P5	T1	Low	2079
P5	T2	Low	4638
P5	T3	Low	3944
P5	T4	Low	2915
P5	T5	Low	12364

Protein-coding genes Genes not assigned as non-coding were classified as protein coding; all the transcripts associated with them were assigned the same biotype.

8.5.4 Removal of spurious genes

After assigning a biotype to each gene, we performed a final polish of the annotation by marking for removal loci where all the transcripts met the following criteria:

- Putative non-coding transcripts lacking transcript support (TR5)
- Putative coding transcript lacking transcript and protein similarity support (TR5,PR5)
- Protein coding transcripts harbouring an in-frame stop-codon

Before discarding these transcripts, we performed an expression estimation against all of our samples using Kallisto v 0.42.5 (Bray et al., 2016); in parallel, we aligned all high-confidence protein coding transcripts from the previous annotation (The International Wheat Genome Sequencing Consortium, 2014) using GMAPL v. 2015-11-20 (Wu and Watanabe, 2005) and asking for the best match with coverage over 90% and identity over 95% (excluding chimeric alignments). Genes were retained if one of their transcripts met at least one of the following conditions:

- Expression level over 0.5 TPM in at least one of our samples, as measured by `Kallisto`
- BLAST hit from the Full-LengtherNEXT analysis with the UniProt database.
- Match against the IWGSC set, with *AED* lower than 1, as measured by Mikado compare

Any gene whose transcripts were all marked for removal, even after these last checks, was excluded from the final annotation. Table S8.7 reports the final number of coding transcripts per each rank.

8.5.5 Assignment of high and low confidence tags

Based on the above ranking, gene models were classified as high and low confidence as follows:

A **High confidence (biotype Protein_coding)** - any protein coding gene where any of its associated gene models meet the following criteria:

Table S8.8: TGACv1 annotation biotype and gene confidence assignment.

Confidence Level	Biotype	Gene Count
High	protein_coding	104091
High	ncRNA	10156
Low	Protein_coding_repeat associated	8556
Low	protein_coding	83217
Low	ncRNA_repeat_associated	1954
Low	ncRNA	9933

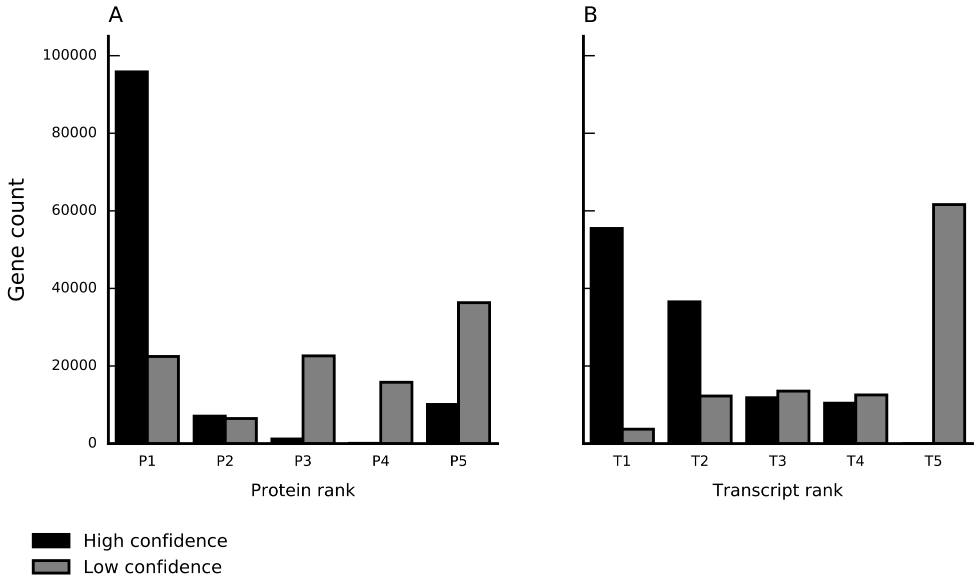

Figure S8.1: Assessment of confidence rankings for the protein coding portion of the wheat gene set. Protein **(A)** and transcript **(B)** classification for high and low confidence genes (gene level) based on classification of the representative gene model.

- PR1 and TR1 to TR4
- PR2 and TR1 to TR4
- PR3 and TR1

B **Low confidence (biotype Protein_coding)**: any protein coding gene where all of its associated transcript models do not meet the criteria to be considered as high confidence protein coding transcripts.

C **High confidence (biotype ncRNA)**: any ncRNA gene where any of its associated gene models meet the following criteria:

- TR1
- TR2

D **Low confidence (biotype ncRNA)**: any ncRNA gene where all of its associated transcript models do not meet the criteria to be considered as high confidence non-coding transcripts.

E **Low confidence (biotype Protein_coding_Repeat_associated, ncRNA_Repeat_associated)** all repeat associated genes are classed as low confidence.

This classification defines four locus biotypes (protein_coding, ncRNA, protein_coding_repeat_associated and ncRNA_repeat associated) and two locus level confidence classifications: "high" or "low". Transcript classifications were harmonised within each gene so that each of them only harbours transcripts of one classification, following the order of rankings in the list above.

The number of genes within each category can be found in Table S8.8, and a graphical summary of the genes associated with each protein and transcript ranking can be found in Figure S8.1.

8.5.6 Assignment of a representative gene model

We assigned a representative model for a gene by selecting a model with the highest confidence ranking (as described in Table S8.7, where a rank 1 is greater than a rank 5 model, i.e., PR1 is better than PR5, TR1 is better than TR5) and lowest *AED* by keeping the order:

1. highest protein rank
2. highest transcript rank
3. lowest *AED*.

For ncRNA genes, we assigned the representative model by considering the order:

1. highest transcript rank
2. lowest *AED*.

We compiled a summary of the annotation statistics in Table 3 of the manuscript.

8.5.7 Assessment of the TGACv1 annotation

Comparison with *B. dystachion* models. We assessed the coherence in gene length between a selected set of TGACv1 Triticum aestivum and Brachypodium distachyon genes. We have downloaded 2707 *Brachypodium distachyon* proteins identified as single copy in *Brachypodium distachyon*, *Oryza sativa*, *Sorghum bicolor*, *Setaria italica* and *Zea mays* from Phytozome 11 (BioMart URL link: `https://goo.gl/5Ujnkj`). The B.distachyon proteins were blasted (ncbi-blast-2.3.0+, maximum evalue 1×10^{-5}) against TGACv1 *T. aestivum* proteins and the reciprocal best hit was selected using a custom perl script. A high coherence in gene length was found between *B. distachyon* proteins and TGACv1 *T. aestivum* proteins (Figure S8.2).

Reconstruction of the gene space in multiple *T. aestivum* assemblies. We assessed how completely the "gene space" was represented in TGACv1 relative to publicly available wheat assemblies by aligning the 1,509,322 PacBio transcripts to each assembly (minimum 95% identity; Figure S8.3). Of the PacBio transcripts 93% could be aligned with greater than 90% coverage to TGACv1, 19% more than to the synthetic W7984 assembly (74%; Chapman et al. (2015)).

Comparison with IWGSC gene models We compared the previous annotation with ours (The International Wheat Genome Sequencing Consortium, 2014; Choulet et al., 2014) by aligning the gene models onto our assembly with GMAPL (version `2015-11-20`; Wu and Watanabe (2005)) with the following command line options:

```
gmapl --no-chimeras -n 1 -f 2 --min-trimmed-coverage=0.90 --min-identity=0.95
```

The alignment has been effectuated separately for the high confidence genes and the low confidence set. The alignments were compared against our annotation with Mikado compare (v. 0.22.0; Venturini et al. (2016)), and binned into four different classes:

1. TGAC model missed (class code in the refmap file: NA, X, x, P, p, i, I, ri, rI, u).

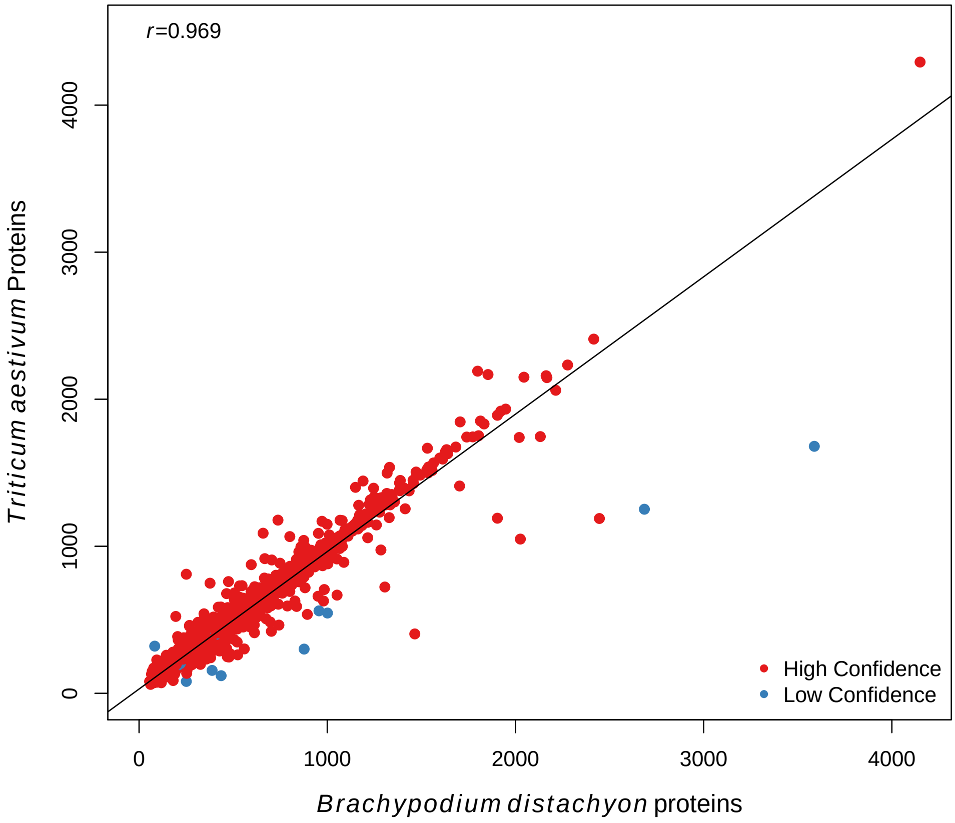

Figure S8.2: Coherence in gene length between *Triticum aestivum* and *Brachypodium distachyon* proteins. Blast analysis (1×10^{-5}) identified 2686 proteins that had reciprocal best hits to 2707 *Brachypodium distachyon* proteins identified as single copy in *B. distachyon*, *O. sativa*, *S. bicolor*, *S. italica*, *Z. mays* (Phytozome). A high coherence in gene length was found between *Triticum aestivum* and *Brachypodium distachyon*, with a correlation coefficient r equal to 0.969.

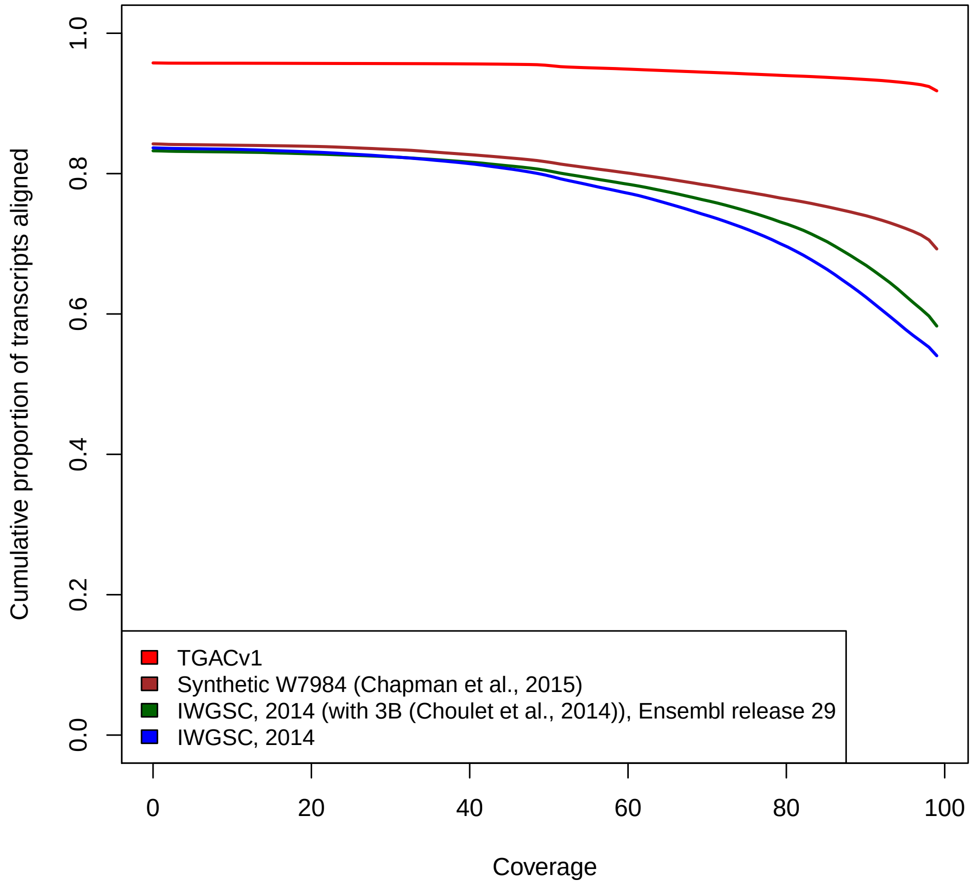

Figure S8.3: Assessment of gene content in different wheat assemblies. PacBio transcripts (1,509,322) were aligned with GMAP (version 2015-11-20; Wu and Watanabe (2005)) to TGACv1 and three public assemblies. The plot shows cumulative proportion of aligned sequences in each assembly.

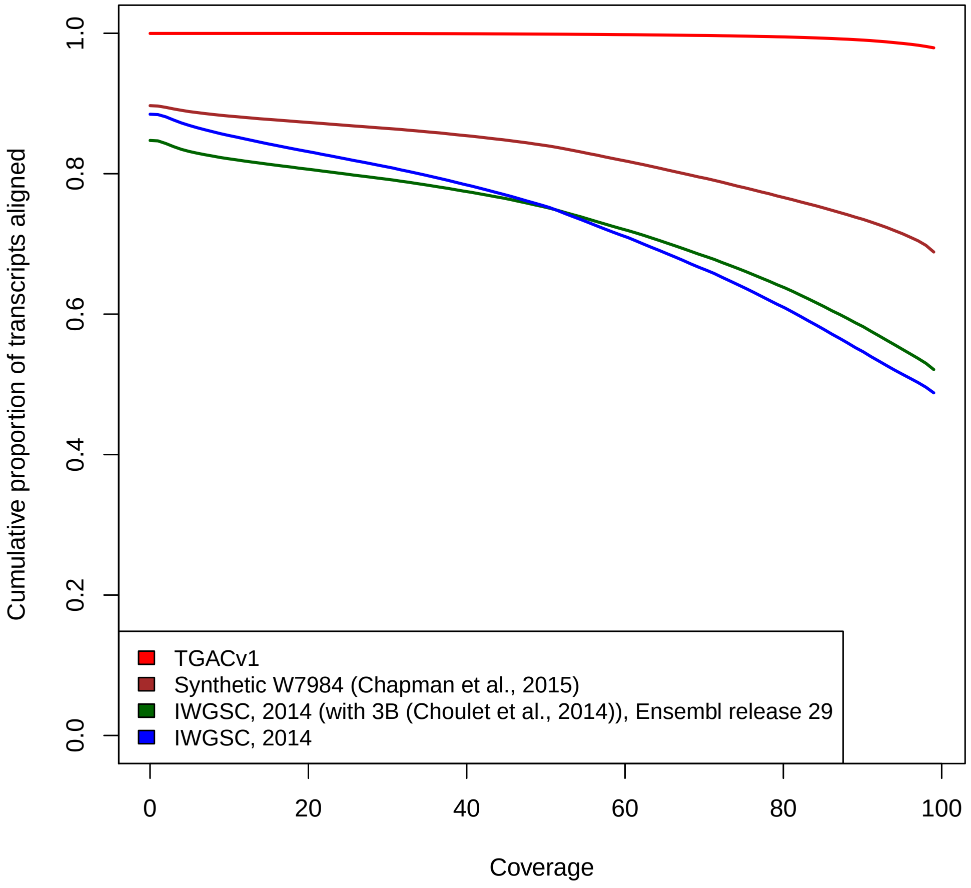

Figure S8.4: Assessment of TGACv1 gene content in public wheat assemblies. TGACv1 transcripts were aligned with GMAP (version 2015-11-20) to TGACv1 and three public assemblies. The plot shows cumulative proportion of aligned sequences in each assembly.

2. Structural difference between the TGAC model and the IWGSC model (class codes in the refmap file: f, j, J, n, h, O, C, mo, m, o, e).

3. IWGSC contained within the TGAC model (class codes in the refmap file: c).

4. Concordance between the two annotations (class codes in the refmap file: =, _)

Results are reported in Figure 3 of the manuscript.

To assess how much of the TGACv1 gene content was contained in other publicly available wheat assemblies we aligned TGACv1 genes and assessed the proportion of TGACv1 models aligned relative to alignment coverage (Figure S8.4).

8.5.8 Evaluation of non-coding RNAs

Comparison with coding models in *T. aestivum* We extracted the GFF3 of the 10,156 high-confidence ncRNA genes of the TGACv1 annotation using the `grep` utility from Mikado v0.24.0; only representative transcripts for each gene were retained. Likewise, we extracted the GFF3 of all coding genes (both high and low confidence). Mikado `compare` was then used to find the best match for each entry in the former GFF in the latter one. For the purposes of this evaluation, class codes in the TMAP file of `u`,`p` and `P` were considered as intergenic, `X` and `x` as matches on the opposite strand, and finally `i` and `I` as intronic.

Alignment against the genomes of progenitors We downloaded the genomes of two progenitors of *Triticum aestivum*, *Triticum urartu* and *Aegilops tauschii*, from EnsEMBL plants release 32. The representative transcripts of the 10,156 high-confidence ncRNA genes of the TGACv1 annotation were aligned against each of these genomes using GMAP v2015-11-20 (Wu and Watanabe, 2005), with the command line options:

```
gmap --no-chimeras -n 5 -f 2 --cross-species
```

The matches were then extracted from the GFF files, filtered for hits with identity and coverage greater than 90%, and merged into a unique list.

8.6 Alternative splicing analysis

RNA-Seq reads generated via the Illumina platform are often too short to cover a full transcript and unambiguously link alternative 5' and 3' splicing events. Furthermore, mapping of relatively short (100–300bp) reads can lead to misalignment and the identification of a substantial number of false positive splice junctions (Sturgill et al., 2013). With different assembly methods showing considerable variation in the number and structure of transcripts assembled we chose to take a conservative approach to annotating alternative splicing in the TGACv1 gene set, giving greater emphasis to long PacBio reads and excluding transcripts with severely truncated coding sequences. To provide a more comprehensive representation of alternative splicing we subsequently integrated transcripts assemblies generated from six strand specific Illumina libraries (Table S8.1, BioProject accession number PRJEB15048). RNA-Seq transcript assemblies were generated from the six samples using cufflinks (v2.2.1) and subsequently merged via cuffmerge (Roberts et al., 2011b), the TGACv1 gene models were provided as reference annotation. The merged transcripts assemblies were filtered to contain transcripts that are novel isoforms to the TGACv1 annotation, i.e. share at least one splice junction with the reference transcript. Splice variants identified from this additional analysis are provided as a separate track in the Ensembl wheat browser `http://plants.ensembl.org/Triticum_aestivum`, and can be retrieved from the Earlham Institute server (see Section 8.8) In order to analyse different alternative splicing events and to identify transcripts that are susceptible to nonsense mediated decay (NMD), a bioconductor package, spliceR (Vitting-Seerup et al., 2014), was used with the output generated from running cuffdiff (Trapnell et al., 2012).

8.7 Functional annotation of protein coding transcripts

All the proteins of our annotation were annotated using AHRD v.3.1 (Hallab et al., 2014). Sequences were blasted against TAIR10 *A. thaliana* protein sequences (Lamesch et al., 2012) and the plant sequences of UniProt v. 2016_05, both SwissProt and TREMBL datasets (The UniProt Consortium, 2014). Proteins were BLASTed using BLASTP+ v. 2.2.31 asking for a maximum e-value of 1. We adapted the standard example configuration file pathtest/resources/ahrd_example_input.yml, distributed with the AHRD tool, changing the following apart from the location of input and output files:

1. we included the GOA mapping from uniprot,
2. The regular expression used to analyse the TAIR header was amended to correct a parsing error to:

```
^>(?<accession>[aA][tT][0-9mMcC][gG]\\d+(\\.\\d+)?)\\s+\\| Symbols
:[^\\|]+\\|\\s+(?<description>([^\\|]+|))(\\s*\\|.*)?\$
```

Concurrently, we analysed the same set of sequences using InterProScan 5.18.57 (Jones et al., 2014). A custom Perl script was used to integrate the ranking, biotype, and functional classification from both tools into a unified file available at: `http://opendata.earlham.ac.uk/Triticum_aestivum/TGAC/v1/annotation/Triticum_aestivum_CS42_TGACv1_scaffold.annotation.gff3.functional_annotation.tsv.gz`.

8.8 Data Access

Sequencing reads generated for this study have been submitted to the European Nucleotide Archive under the accession code PRJEB15048. The annotation is available in Ensembl Plants genomic repository (release 32) at `http://plants.ensembl.org/Triticum_aestivum` and from the Earlham Institute server at `http://opendata.earlham.ac.uk/Triticum_aestivum/TGAC/v1/annotation`. The latter repository contains the following files:

- TGACv1 annotation, in GFF3 format:
  - `Triticum_aestivum_CS42_TGACv1_scaffold.annotation.gff3.gz`
- Sequences for the transcript models of TGACv1 cDNAs, CDS and proteins:
  - `Triticum_aestivum_CS42_TGACv1_scaffold.annotation.gff3.cdna.fa.gz`
  - `Triticum_aestivum_CS42_TGACv1_scaffold.annotation.gff3.cds.fa.gz`
  - `Triticum_aestivum_CS42_TGACv1_scaffold.annotation.gff3.pep.fa.gz`
- Functional annotation of TGACv1 models:
  - `Triticum_aestivum_CS42_TGACv1_scaffold.annotation.gff3.functional_annotation.tsv.gz`
- Annotation of alternative splicing events (see Section 8.6), in both GFF3 and GTF format:
  - `Triticum_aestivum_CS42_TGACv1_scaffold.annotation.AS.gff3.gz`
  - `Triticum_aestivum_CS42_TGACv1_scaffold.annotation.AS.gtf.gz`

9 Proteomics

Proteome profiling was conducted through reanalysis of Duncan et al. (2017). Briefly, organ and developmental stage samples were collected from both field and lab grown *Triticum aestivum* cv. Wyalkatchem. Frozen samples were crushed using mortar and pestle before protein extraction with the chloroform / methanol procedure (Wessel and Flügge, 1984) prior to tryptic digestion. A peptide level prefractionation was performed according to Yang et al. (2012) before reversed phase C18 LC/MS analysis on an Agilent 6550 Q-ToF. Spectra were matched against the combined high and low confidence protein coding peptide sequence set (249,547 sequences) with CometUI (2016.01 rev. 2; Eng et al. (2013)) precursor tolerance +/- 50 ppm, variable oxidation of methionine, fixed carbamidomethyl C. Results were validated through the Trans-Proteomic Pipeline, with the tools peptide and protein prophet (TPP v4.8.0; Deutsch et al. (2010)). A 2% peptide level FDR cutoff was calculated through the inclusion of reversed decoys of the protein sequences. Peptide matches to TGACv1 genes and transcripts are provided as Supplementary File **S9**.

10 Orthologous gene family analyses

10.1 OrthoMCL gene family clustering of wheat subgenome genes

Gene family clusters were defined from the bread wheat high-confidence class genes, separated for their subgenome origin (A, B and D) and undefined origin ("U") using OrthoMCL software version 2.0 Li (2003). In a first step, pairwise sequence similarities between all input protein sequences were calculated using BLASTP with an e-value cut-off of 1×10^{-5}. Markov clustering of the resulting similarity matrix was used to define the ortholog cluster structure, using an inflation value (`-I`) of 1.5 (OrthoMCL default).

The input datasets were:

- Bread Wheat A genome (high-conf): 32,452 genes
- Bread Wheat B genome (high-conf): 34,713 genes
- Bread Wheat D genome (high-conf): 32,724 genes
- Bread Wheat genes of unknown origin (high-conf): 4202 genes

Splice variants were removed from the data sets, keeping the representative gene model, and data sets were filtered for internal stop codons and incompatible reading frames. A total of 87,519 coding sequences from these three datasets were clustered into 25,132 gene families (clusters). An overview of the cluster structure is shown in Figure S10.1. We identified 13,070$\times$ 3 genes found in a 1:1:1:0 ratio in the A,B,D and U subgenomes (triads); this set was filtered to 9642 triads with $>90\%$ identity in pairwise BLASTP alignments between A,B and D genes (Supplemental file **S10**). The same OrthoMCL analysis was also performed with all TGACv1 gene models, both high- and low-confidence in a separate run (Figure S10.2).

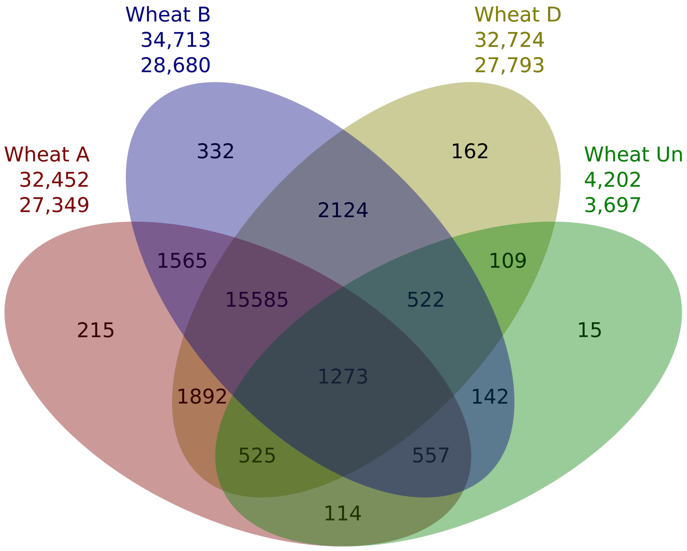

Figure S10.1: OrthoMCL clustering of bread wheat genes (HC class) from the A, B and D subgenome and unclassified origin ("Un"). The first number under the species name gives the total number of transcripts predicted, the second number is the number of transcripts in OrthoMCL clusters. The difference gives the number of singletons (genes not clustered).

10.2 OrthoMCL gene family clustering of the bread wheat genome and related species

Following the protocol used in section 10.1, OrthoMCL was used to define gene family clusters at a species level, using as datasets the bread wheat high-confidence class genes, the annotated gene sets of three grasses from diverse grass sub-families, and Arabidopsis thaliana (Figure S10.3). The input datasets were:

- Bread Wheat A genome (high-conf): 32,452 genes
- Bread Wheat B genome (high-conf): 34,713 genes
- Bread Wheat D genome (high-conf): 32,724 genes
- Bread Wheat genes of unknown origin (high-conf): 4,202 genes
- Sorghum bicolor v2.1: 33,032 genes
- Brachypodium distachyon v2.1: 31,694 genes

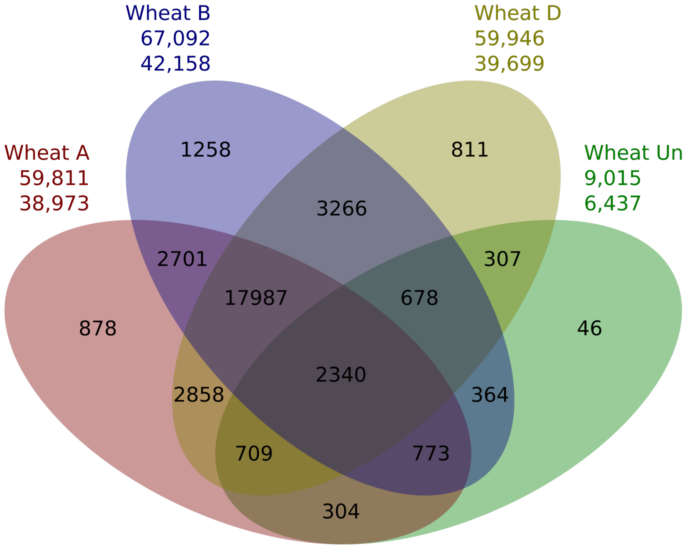

Figure S10.2: OrthoMCL clustering of bread wheat genes (all confidence classes) from the A, B and D subgenome and unclassified origin ("Un"). The first number under the species name gives the total number of transcripts predicted, the second number is the number of transcripts in OrthoMCL clusters. The difference gives the number of singletons (genes not clustered).

- Rice MSU7.0: 39,049 genes
- Arabidopsis thaliana TAIR10: 27,416 genes

Coding sequences from these five species were clustered into 29,862 gene families.

In a separate run, the same OrthoMCL analysis was performed with all bread wheat gene models given as as a single species (Figure S10.3).

10.3 GO over-/under-representation for specific groups/singletons

Over-/under-representation of gene ontology (GO) terms in specific gene families and subsets (see Section 10.4) were analysed via hypergeometric testing using the functions GOstats (Falcon and Gentleman, 2007) and GSEABase (Morgan et al., 2008)from the bioconductor R package against a universe of all genes with GO annotations. Revigo (Supek et al., 2011), which removes redundant and similar terms from long GO lists by semantic clustering was applied to visualise the enrichment results.

10.4 Expanded gene families in OrthoMCL and GO over-representation within

From the OrthoMCL analyses described in Sections 10.1 and 10.2, we extracted gene models from different distinct OrthoMCL subsets:

A **"Subgenome-specific" set**: Wheat genes in groups/clusters which are subgenome-specific (cluster/group contains only genes from subgenome A, B or D) and cluster size greater than 1;

B **"Subgenome-singletons" set**: Wheat genes which were not clustered within any of the OrthoMCL groups, termed "Singletons", separated by their subgenome origin;

C **"Wheat-subgenome-expanded" set**: Wheat genes in groups/clusters where the gene copy number is significantly (p-value less than 0.05) expanded in one of the subgenomes relative to the other subgenome including clusters (size greater than one) that only consist of the respective subgenome genes;

D **"Wheat-expanded(A/B/D)" set**: Wheat genes, separated by subgenome origin, in groups/clusters where the Wheat gene copy number is significantly expanded (p-value less than 0.05) relative to any of the other species contained including clusters (size greater than one) that only consist of Wheat genes.

The individual gene sets were analysed for over-represented GO terms from all GO categories "biological process", "molecular function" and "cellular component". Results are summarized and visualized in Supplemental file **S2**.

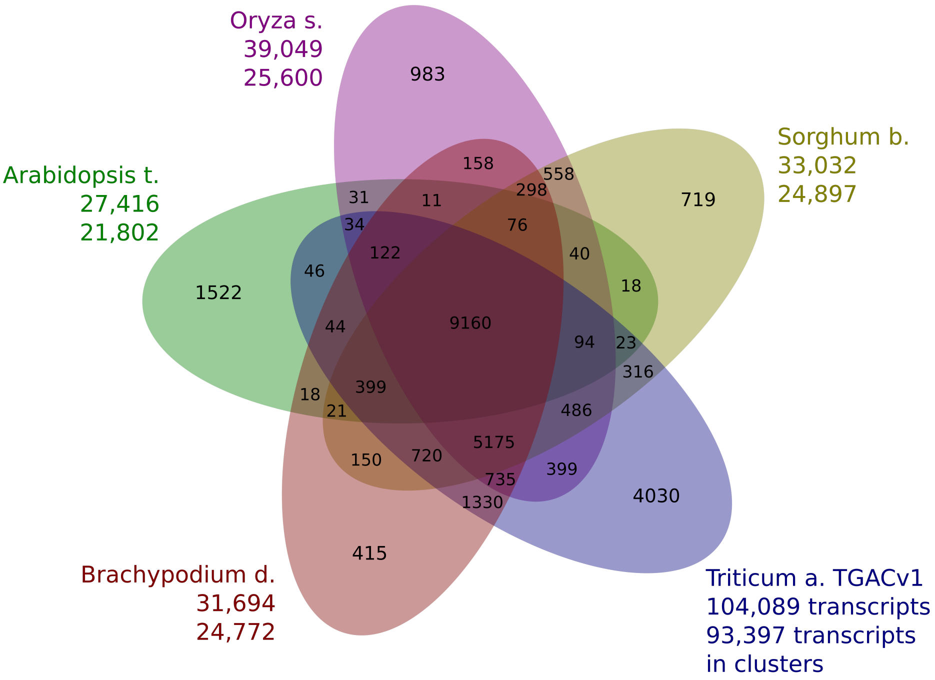

Figure S10.3: OrthoMCL clustering of bread wheat genes (HC confidence class) from the A, B and D subgenome and unclassified origin ("Un") together as a single species, against the gene complements of Arabidopsis, Sorghum, Rice and Brachypodium. The first number under the species name gives the total number of transcripts predicted, the second number is the number of transcripts in OrthoMCL clusters. The difference gives the number of singletons (genes not clustered).

11 Gene expression analyses

11.1 Expression quantification and analysis

11.1.1 Gene expression quantification

Wheat gene expression quantification was carried out as described in Borrill et al. (2016) using kallisto v0.42.3 (Bray et al., 2016) to pseudoalign reads to the complete TGAC transcriptome (including both high and low confidence genes) as a reference. The SRA studies included are listed in S11.1. For paired end reads *kallisto* was run using default parameters with 100 bootstraps (`-b 100`). For single end reads kallisto was run using 100 bootstraps (`-b 100`) in the single end read mode (`--single`), the average fragment length used was 150 bp (`-l 150`) with a standard deviation of 50 (`-s 50`) - these values were taken as an average of reported fragment lengths for studies included.

Table S11.1: SRA studies analysed with expVIP using TGAC gene models as a reference.

Study identifier	Summary	Total reads	Reads mapped to TGAC	Reads mapped to IWGSC	Reference
DRP000768	phosphate starvation in roots and shoots	118,053,746	104,886,994 (88%)	84,529,715 (72%)	Oono et al. (2013)
ERP003465	fusarium head blight infected spikelets	1,827,362,091	1,633,149,812 (89%)	1,357,197,955 (74%)	Kugler et al. (2013)
ERP004505	grain tissue-specific developmental timecourse	873,709,556	718,777,030 (54%)	475,184,621 (82%)	Pfeifer et al. (2014)
SRP004884	flag leaf downregulation of GPC	209,427,573	148,280,320 (72%)	121,855,143 (58%)	Cantu et al. (2011)
SRP013449	grain tissue-specific developmental timecourse	132,702,451	110,682,153 (83%)	82,417,257 (62%)	Gillies et al. (2012)
SRP017303	stripe rust infected seedlings	33,361,836	15,622,370 (47%)	13,732,210 (41%)	Cantu et al. (2013)
SRP022869	Septoria tritici infected seedlings	100,582,632	71,948,196 (72%)	63,155,877 (63%)	Yang et al. (2013)
SRP028357	shoots and leaves of nulli tetra group 1 and group 5	3,304,500,117	2,918,789,524 (88%)	2,258,692,000 (68%)	Leach et al. (2014)
SRP029372	grain tissue-specific developmental timecourse	101,477,759	26,992,810 (22%)	17,525,439 (17%)	Li et al. (2013)
SRP038912	comparison of stamen pistil and pistilloidy expression	217,315,378	196,322,732 (90%)	153,009,134 (70%)	Yang et al. (2015)
SRP041017	stripe rust and powdery mildew infection timecourse	395,463,786	325,434,104 (82%)	272,228,560 (69%)	Zhang et al. (2014a)
SRP041022	developmental time-course of synthetic hexaploid	134,641,113	120,448,445 (90%)	84,583,556 (63%)	Li et al. (2014)
ERP008767	grain tissue-specific expression at 12 days post anthesis	45,213,827	36,971,938 (82%)	26,420,708 (58%)	Pearce et al. (2015)
SRP045409	drought and heat stress time-course in seedlings	921,578,806	592,272,829 (64%)	533,928,182 (58%)	Liu et al. (2015)
ERP004714	developmental time-course of Chinese Spring	1,536,051,415	1,340,790,669 (88%)	1,066,712,760 (69%)	Choulet et al. (2014)
SRP056412	grain developmental timecourse with 4A dormancy QTL	1,875,916,011	1,082,551,207 (57%)	808,809,053 (43%)	Barrero et al. (2015)
PR-JEB15048	developmental time-course of Chinese Spring	824,241,135	631,301,185 (77%)	N/A	This study.

11.1.2 Differential gene expression analysis

Differential gene expression analysis was carried out on the kallisto output abundance files using sleuth (Pimentel et al., 2016). Default settings were used except that the maximum number of bootstraps considered was 30 (`max_bootstrap = 30`). For the integrated disease and stress analysis each sample was compared to the control sample from the study from which it originated. Genes with a FDR adjusted p-value (q-value) less than 0.001 were considered differentially expressed.

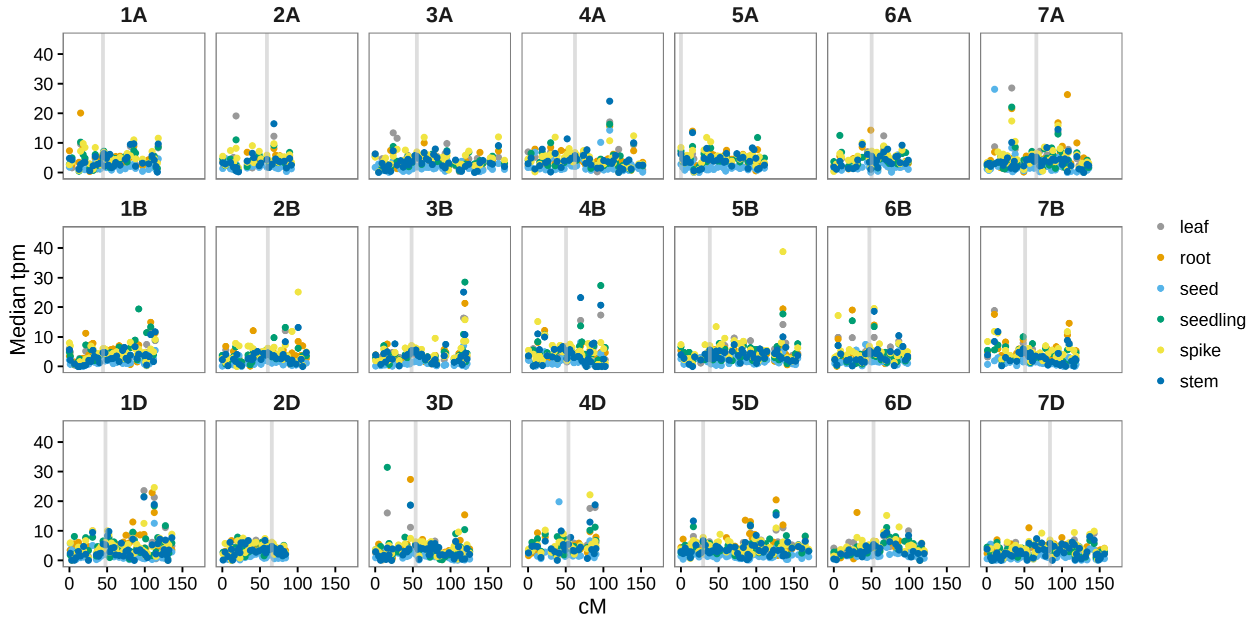

Figure S11.1: Median gene expression level per chromosome bin. cM position was determined by BLAST of TGAC scaffolds to the Chapman scaffold which had POPSEQ position information. Only bins with 3 or more genes were included. Outliers above expression level 45tpm were excluded from the graph. Grey vertical lines indicate centromere position.

11.1.3 Visualisation of gene expression

The quantified gene expression from kallisto were visualised using the expVIP platform (Borrill et al., 2016). It is displayed at `www.wheat-expression.com`.

11.2 Gene expression across 17 diverse RNA-seq studies

We used expVIP (Borrill et al., 2016) to analyse 16 wheat gene expression studies from the short read archive (SRA) from a range of tissues, developmental stages and stress conditions alongside the six RNA samples sequenced during the course of this study (Table S11.1). In total these 424 individual samples contained 12.6 billion reads of which 10 billion mapped to the TGAC transcriptome containing 273,739 genes. This average mapping rate of 75% of reads is higher than the 59% of reads which mapped to the previous IWGSC gene models suggesting that the TGAC transcriptome is more complete. We found that 95% of genes (260,079) had at least 1 read mapping to them, and 58% of genes (160,074) were expressed in at least one samples at over 2tpm which has been advocated as the cut-off for real expression over noise (Wagner et al., 2013). The percentage of genes expressed over the background noise level of 2tpm is relatively low (58%) which may be because the TGAC gene models also include non-coding RNAs which are generally expressed at very low expression levels and low confidence gene models which are not supported by evidence from other species. If we only include high confidence gene models 78% of genes are expressed at over 2tpm. To facilitate access to these RNA-seq datasets we have updated `http://www.wheat-expression.com/` to show gene expression levels for each TGAC gene of interest across all the 17 different studies. The visualisation interface can be filtered and sorted by the viewer according to the origin of each sample in terms of tissue, age, stress and variety. One gene and its homoeologs can be displayed as a bar graph or multiple genes can be displayed as a heatmap.

11.3 Gene expression patterns across chromosome regions

To investigate whether specific chromosomal domains influence the gene expression level we examined gene expression across the length of the chromosomes using genetic map assignments described in Section 5. We found that in general the median expression of genes was similar throughout most chromosomes (Figure S11.1). However certain chromosomal regions had much higher expression across several or all of the six tissues examined and these "enhanced expression regions" were located outside of centromeric regions.

11.4 Analysis of homoeolog gene expression in stress conditions

We identified 9642 triads which had a 1-1-1 relationship between the A, B and D genome copies (Section 10.1). To understand the roles of the three homoeologous copies within triads to a range of stress conditions we leveraged existing RNA-seq data for seedlings (Table S11.2). Gene expression quantification and differential expression analysis was carried out as described in Sections 11.1.1 and 11.1.2. Within each triad we classified changes in response to stress in each homoeolog as either up-regulation (over 2-fold change), down-regulation (under 0.5-fold change) or flat (between 0.5 to 2 fold change); all tests were considered statistically significant with q lower than 0.001. Each triad was then classified according to the number of homoeologs differentially expressed and their direction of change Table S11.3.

Table S11.2: RNA-seq samples used to analyse the response of homoeologous genes to stress conditions.

Study	Age	Conditions	Replicates
SRP041017	7 days	Stripe rust 24 h	3
		Stripe rust 48 h	3
		Stripe rust 72 h	3
		Powdery mildew 24 h	3
		Powdery mildew 48 h	3
		Powdery mildew 72 h	3
SRP045409	7 days	Drought stress 1 h	2
		Drought stress 6 h	2
		Heat stress 1 h	2
		Heat stress 6 h	2
		Drought and heat stress 1 h	2
		Drought and heat stress 6 h	2

Table S11.3: The expression patterns of homoeologs within triads in response to stress treatments. *For triads where two homoeologues are up or down regulated, the third homoeologue could not be expressed in the opposite direction to avoid double counting of "opposite" class triads.

Condition	0	1 up	1 down	2 up*	2 down*	3 up	3 down	Opposite
`drought_1h`	8,588	866	148	31	0	0	0	9
`heat_1h`	6,931	1,731	707	142	17	3	0	111
`drought_heat_1h`	5,941	2,008	1,129	214	68	10	1	271
`drought_6h`	6,248	1,521	1,354	148	110	1	1	259
`heat_6h`	5,288	1,780	1,728	195	211	5	3	432
`drought_heat_6h`	4,965	1,677	2,028	185	253	8	8	518
`mildew_24h`	8,793	607	218	15	2	0	0	7
`mildew_48h`	8,802	180	640	4	12	0	0	4
`mildew_72h`	9,184	24	425	0	6	0	0	3
`yellow_rust_24h`	9,069	267	290	3	7	0	0	6
`yellow_rust_48h`	9,455	13	172	0	2	0	0	0
`yellow_rust_72h`	9,342	41	257	0	1	0	0	1

In triads in which two homoeologs were up- or down-regulated, the A, B and D genome were represented equally (chi-squared test $p = 0.517$ and $p = 0.243$ respectively). Similarly in triads in which one homoeolog was down-regulated the three genomes were represented equally (chi-squared test $p = 0.537$). However in triads in which one homoeolog was up-regulated the three genomes did not respond equally, with the D genome being more responsive to stress conditions (the numbers of triads with one homoeolog up-regulated in which the A, B and D genome homoeolog was upregulated were 3390, 3494 and 3831 respectively, chi-squared test $p = 3.45 \times 10^{-7}$). In triads with opposite patterns of homoeolog expression the B genome was more frequently up-regulated than the other two genomes (the numbers of triads with opposite homoeolog expression patterns in which the A, B and D genome homoeolog was upregulated were 526, 606 and 538 respectively, chi-squared test $p = 0.035$), however all three genomes were as likely as each other to be the down-regulated genome in triads with opposite homoeolog expression patterns (chi-squared test $p = 0.0745$).

11.5 Homoeologous gene expression analysis

We decided to investigate expression of homoelogous genes using an ad-hoc approach similar to that described in Liu et al. (2015). We decided to focus on three studies for this analysis: SRP041017, SRP045409, and ERP004505. For each of our 9642 triads, we verified in each condition whether their expression was balanced by performing a paired fisher test between the A and B gene, B and D gene, and A and D gene; as in Liu et al. (2015), we compared the two expression values for the triads against the sum of all the expression values for the subgenome in the condition, minus the expression of the gene under analysis (equation (1)); Fisher test as implemented in Scipy v. 0.18.0 Jones et al. (2001)). We corrected our p-values using the standard Benjamini-Hochberg method for False Discovery Rate, as implemented in Stasmodels 0.6.1 (Seabold and Perktold, 2010).

$$F(x,y) = Fisher((tpm_x, tpm_y), ((\sum_{\chi=1}^{\Xi} tpm_\chi) - tpm_x, (\sum_{\upsilon=1}^{\Upsilon} tpm_\upsilon) - tpm_y)) \quad (1)$$

The probability for two homoeologous x, y genes to be expressed at an unbalanced level was calculated by performing a Fisher exact test of their expression, in TPM, versus the sum of all TPM values of the triads for their respective subgenomes Ξ and Υ excluding the couple of genes themselves.

Expression values for the analysed triplets, and the accession codes for the RNA-Seq raw data, can be found in Supplementary file **S11**, while the Fisher test evaluation results are reposted in Supplementary file **S12**.

Subsequently, we considered a pairwise comparison within a replicate as significant if the following conditions verified:

1. at least one of the two genes compared had to have an expression level greater than 0.01 TPMs (to exclude lowly expressed loci).

2. the comparison had to have a corrected p-value lower than 0.05.

3. Either one of the two genes had an expression of 0, or the absolute log2 Fold Change between the two genes was 1 or above.

Each comparison was assigned one of three signed values (0 for no differential expression, 1 for an over-expression of the first gene compared to the second, -1 for an under-expression of the first gene compared to the first, NA if neither gene was expressed at a sufficiently high level). A pair of genes was considered as unbalanced if all the replicates were found to have a significant and coherent difference in expression between the two members (ie. if in a couple the first gene was significantly under-expressed in a sample and significantly over-expressed in another replicate or without evidence for a difference in expression, the comparison would have been called as inconclusive). A triad was called as unbalanced if at least one of its internal pairs was unbalanced.

Expression values for the analysed triplets can be found in supplementary file **S11**, while the Fisher test evaluation results are reposted in supplementary file **S12**. The final evaluation is reported in supplementary file **S13**.

11.6 Gene expression in syntenic loci

Collinearity was detected between the high confidence genes annotated on the wheat sub-genomes using MCScanX (Wang et al., 2012). Protein sequences for the high confidence genes were used in all versus all BLASTP analysis (Section 10.1).

Conserved blocks were defined as a set of at least 5 genes (anchors) in the same order between 2 sub-genomes, with a maximum of 25 spacer genes between the anchors in a collinear block. A total of 91 pairwise collinear blocks were identified, from these 12 collinear blocks of the A, B and D sub-genomes were identified. (Supplemental file **S14**). Pairwise alignments between two syntenic blocks were calculated using LAST (Frith et al., 2010). Adjacent syntenic alignments were joined into single larger syntenic alignments using the UCSC Chain/Net pipeline (Kent et al., 2003). Expression levels of genes in the blocks were assessed using triplicated RNAseq data from Chinese Spring root and shoot tissues, and from 10day and 20 whole endosperm tissue (SRA studies DRP000768 and ERP004505). Gene expression levels were expressed as $\log_2(TPM+1)$. Unbalanced expression was defined as a significant difference in expression between homoeologues in any of the four tissues measured, defined as the expression of any homoeologue having greater than $4(TPM+1)$ expression levels than another homoeolog. Collinear relationships, synteny links, and expression levels for the genes on four syntenic blocks selected to illustrate different patterns of gene expression were plotted using SyntenyPlot (`https://github.com/lufuhao/SyntenyPlot`). Promoter motifs were identified using PlantCARE, and transcription start sites were identified from the TGACv1 annotation. Synteny views of genes in AA, BB, and DD scaffolds in 4 selected blocks are shown in Figure S11.2, showing different patterns of conservation of gene and repeat order and gene expression.

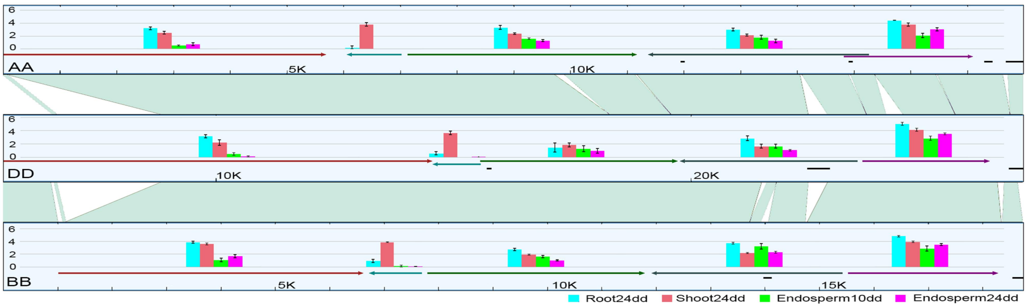

(A) This block illustrates a high degree of similarity in the A, B and D genomes, with similar patterns of gene expression. AA: reverse complement of TGACv1_scaffold_195481_3AL:1–18115, BB: TGACv1_scaffold_224116_3B:34006–53294, DD: TGACv1_scaffold_250027_3DL:14626–41915.

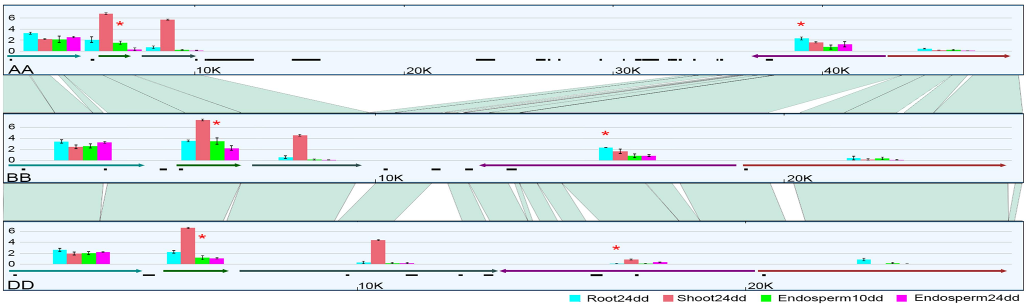

(B) This block shows the interspersion of a tract of repeats in the A genome compared to the B and D genomic blocks. A gene encoding a histone-lysine N methyltransferase in the D genome is expressed at lower levels in root tissues. AA: TGACv1_scaffold_288349_4AL:28586–78590, BB: reverse complement of TGACv1_scaffold_328157_4BS:112255–138693, DD: TGACv1_scaffold_361457_4DS:18746–46470.

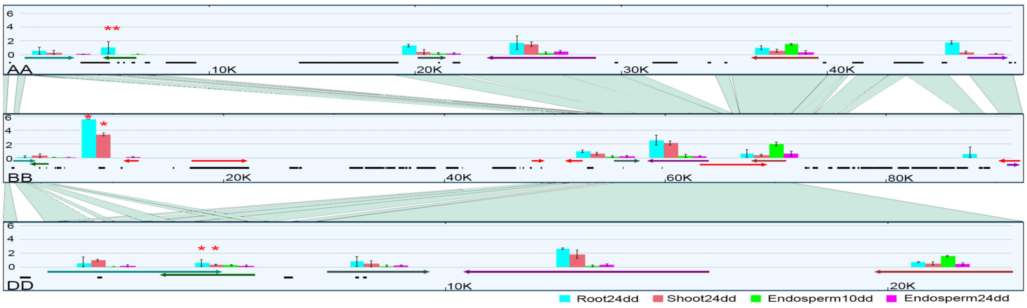

(C) This block shows unbalanced expression of an uncharacterised protein in the A, B and D genomes. There are major differences in the repeat composition in the A and B genomes compared to the D genome AA: TGACv1_scaffold_375286_5AL:25956–75758, BB: TGACv1_scaffold_404593_5BL:66316–159457, DD: reverse complement of TGACv1_scaffold_435472_5DL:7315–31129.

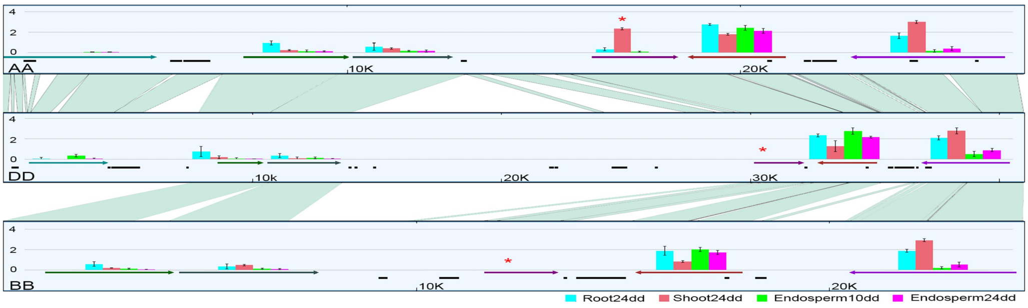

(D) A gene encoding a cytochrome P450 72A14-like protein is highly expressed in shoot tissues in the A genome, and the homoeologous B and D genes are not detectably expressed. AA: TGACv1_scaffold_392578_5AS:182276–210005, BB: TGACv1_scaffold_424311_5BS:12106–37625, DD: TGACv1_scaffold_456510_5DS:23146–64545.

Figure S11.2: Four syntenic blocks showing conserved gene order and different patterns of gene expression, and repeat and gene interspersions. Genome segments are arranged to reveal patterns of maximum conservation. Unbalanced gene expression is identified by a red asterisk above the bar graph of expression levels. The x axis scale is in bp. Bar plots the $\log_2(TPM+1)$ of gene expression (0-6) on the y axis. Asterisks above gene expression bar plots indicate unbalanced expression. Arrows indicate genes, with homoeologous genes shown in the same colour. Red arrows mark no-syntenic genes. Black boxes show repeat-masked regions.

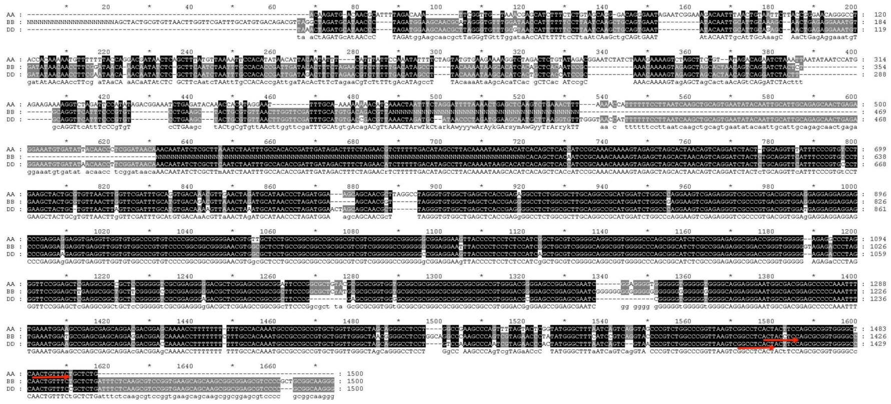

(A) Alignment of the fourth homoeologous group of genes in Figure **A**

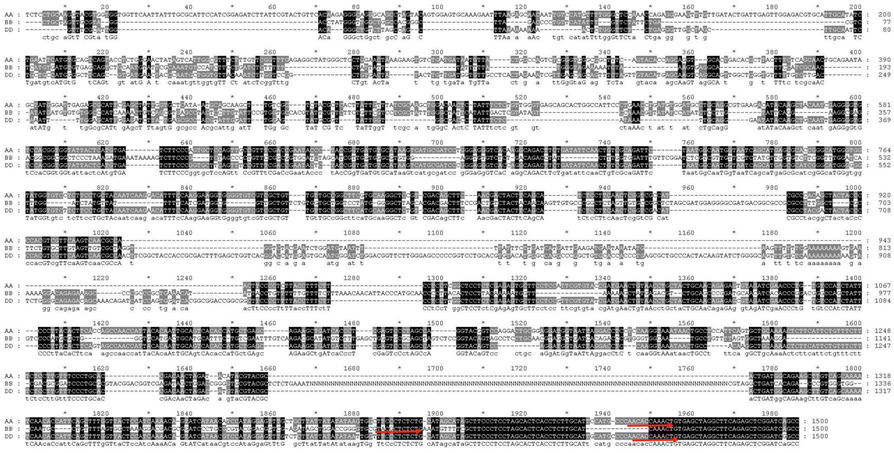

(B) Alignment of the second homoeologous group of genes in Figure **C**

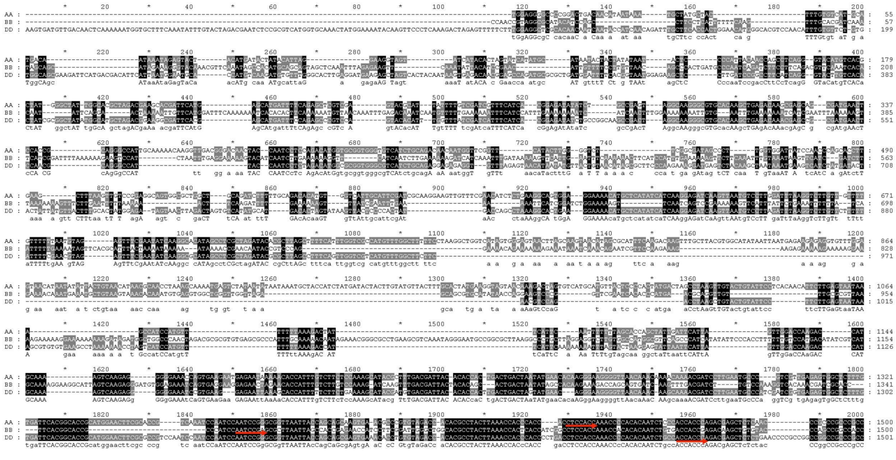

(C) Alignment of the fourth homoeologous group of genes in Figure **D**

Figure S11.3: Examples of promoter region divergence in homoeologous genes showing unbalanced expression in Figure S11.2, above. Promoter regions end are 1500bp upstream of the initiating ATG codon. Red arrows indicate the location of transcriptional start sites.

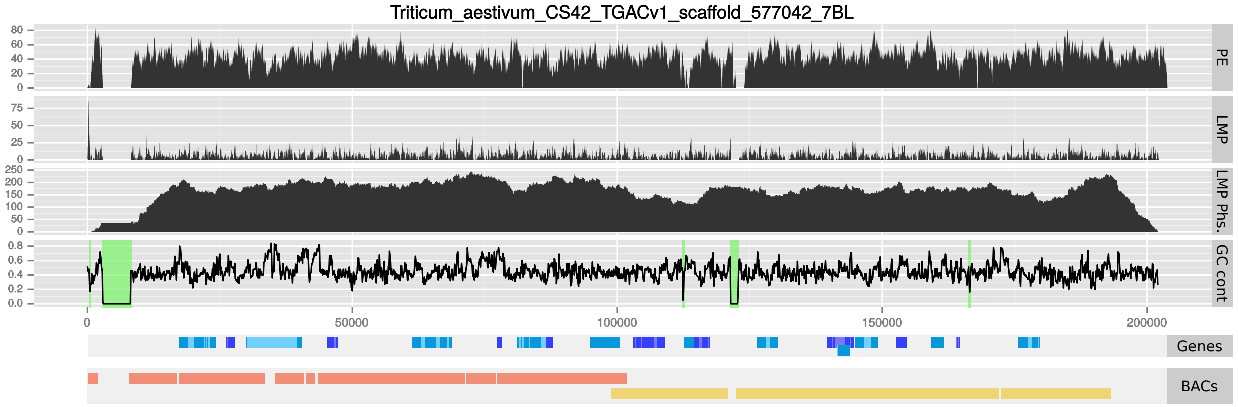

Figure S12.1: Scaffold 577042 of the TGACv1 assembly with resistance genes, aligned BACs and read data. The tracks from top to bottom show coverage of paired-end reads, coverage of mate-pair reads, coverage of mate-pair fragments, GC content and N regions (highlighted in green), resistance genes, and BACs. There are two BACs in 7 and 4 contigs, respectively, and 20 resistance genes.

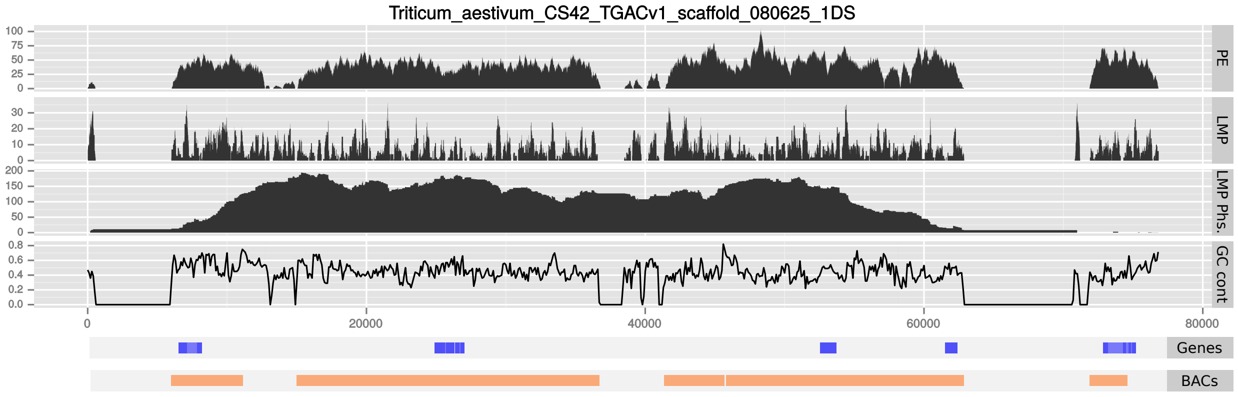

Figure S12.2: Scaffold 080625 of the TGACv1 assembly with gluten genes, aligned BACs and read data. The tracks from top to bottom show coverage of paired-end reads, coverage of mate-pair reads, coverage of mate-pair fragments, GC content and N regions (highlighted in green), gluten genes, and BACs. There is one BAC in 5 contigs and 6 resistance genes.

12 Gene families of agronomic importance

12.1 Disease resistance genes

Disease resistance genes were predicted by analysing the domain architectures with previously established pipeline (Sarris et al., 2016) which utilises the Pfam annotation of functional domains. In addition, we scanned high confidence proteomes for previously identified NLR MEME motifs (Jupe et al., 2012) and analysed the results with NLR-parser (Steuernagel et al., 2015) to predict NLR-associated motifs and assess CC-NBS-LRR type disease resistance genes. The fragmented and complete transcript genes were also compared by the presence of start and stop codons in predicted transcripts. The sequences for the resistance genes are provided in Supplementary files **S15**, **S16**, **S17** and **S18**.

12.2 Gluten genes

Due to the challenges of annotating repeat rich gluten genes, we reviewed all regions with nucleotide similarity to publicly available gluten sequences (NCBI, Zhang et al. (2014b); Pfeifer et al. (2014)) with blastx (e-10) or GMAP (at least 95% identity, at least 40% coverage) via the Apollo browser (`http://genomearchitect.github.io/`).

Due to the challenges of annotating repeat rich gluten genes, we reviewed regions with nucleotide similarity to publically available gluten sequences (NCBI, Zhang et al. (2014a), Pfeifer et al. (2014)) with blastx (e-10) or GMAP (at least 95% identity, at least 40% coverage) via the Apollo browser (`http://genomearchitect.github.io/`). The manually updated annotations are provided as supplementary files **S19**, **S20**, **S21**, **S22** and **S23**. Gluten pseudogenes are provided in a separate Supplementary file, **S24**.

12.3 Gibberellin genes

Wheat genomic sequences corresponding to genes from the gibberellin biosynthesis, inactivation and signalling pathways were identified in the TGACv1 assembly by BLASTN, using previously identified sequences from wheat (Pearce et al., 2015) or rice (Hirano et al., 2008) and aligned using Geneious (`http://www.geneious.com`).

12.4 BAC analysis

The two BACs we have used in our examples are from a larger set of BACs which we were sequenced and assembled. Briefly, BACs were selected for sequencing from a HinDIII partial digest BAC library (Allouis et al., 2003). BAC DNAs were miniprepped (Sambrook and Russell, 2006), treated with ATP dependent DNase to remove E. coli genomic DNA, and individually barcoded Illumina Nextera libraries prepared. Nextera libraries were sequenced using Illumina chemistry 2×250bp cycles (paired end). The reads were demultiplexed and filtered to remove the BAC vector, E. coli genome, and wheat chloroplast and mitochondria sequences. The remaining reads for each BAC were then assembled using DISCOVAR *de novo* (Weisenfeld et al., 2014) and then trimmed to remove any remaining vector sequence from the contigs. The assemblies had an average content of 111kbp and an average contig N50 of 16.7kbp. The assemblies of the BACs used in Figures S12.1 and S12.2 are given in Supplemental files **S25** and **S26**, respectively.

13 Authors' contributions

BJC, LV, DH, CF, DS, FDP and MDC designed the sequencing experiments. DNA and RNA was isolated by NMcK or GY, and sequencing libraries prepared by DH, TB and JL. BJC, GGA, JW and FDP designed and implemented the assembly strategy. GGA, JW and BJC performed the genome assembly. LV, GKa and DS designed and implemented the annotation strategy, including manual curation of gluten genes and alternative splicing analysis. HG (MIPS) performed the global analysis of repeats. CU performed detailed analysis of breakpoints versus previous assemblies. MS and GH (MIPS), PK (EBI) and DS performed global gene family analyses. CS, DR and KVK designed and implemented the approach to anchor the assembly onto the genetic map, predicted translocations and designed the validation strategy. R-gene family analyses and gluten gene family analyses was performed by CS, DR and KVK. Analyses of BACs was done by GKe and MDC. Validation of predicted translocations was performed by MDC, AC, NP and LPA. PB, CU, RRG, LV, DS, F-HL and MWB performed gene expression analyses and JT, OD, AHM proteome profiling and analysis. AP performed analyses of gibberellin pathways. DMB, GN, AK, GKa, DS, RPD, and PJK integrated assemblies, annotations and sequencing reads into public databases. CF and HC provided project management. MWB, LV, DS, GKe and MDC wrote the manuscript and all authors contributed to the text.

14 File list

S1 **Supplemental Information** Additional information for the main article.

S2 Gene ontology enrichment results for singleton and expanded OrthoMCL families (see Section 10)

S3 List of all potential translocation events supported by OrthoMCL outlier triads as described in Section 6.1.

S4 WGS map with corrected bins.

S5 The TGACv1 map (scaffold id, chromosome, cM; only class 1 scaffolds)

S6 Scaffold, Position on TGACv1 map (chromosome:cM), and Map classification for all TGACv1 scaffolds that had at least one matching WGS marker. For class 1 scaffolds (assigned to unique genetic bin), Position has only one entry, while class 2 (assigned to ambiguous bins on the same chromosome), class 3 (assigned to ambiguous bins on homoeologous chromosomes), and class 4 (assigned to at least two non-homoeologous chromosomes) scaffolds have multiple entries, separated by ';'. See Section 5.

S7 Primers used for translocation confirmation.

S8 AUGUSTUS config file used in gene prediction.

S9 Peptide matches to TGACv1 genes and transcripts

S10 List of the 9642 triads of homoeologous genes, as defined in Section 10.1

S11 Expression values, in TPM, for the transcripts in CS42.triplets.tsv across the analysed samples. See Section 11.5

S12 Pairwise Fisher test for the expression within a triad within each replicate. See Section 11.5

S13 Evaluation of whether members of a triplet were expressed in a balanced or unbalanced way in a given sample. See Section 11.5.

S14 Collinear blocks identified between A,B and D genomes. See Section 11.6.

S15 Sequences of all NBS CDS in Fasta format. See Section 12.1

S16 Sequences of all NBS-LRR CDS in Fasta format. See Section 12.1.

S17 Sequences of all translated NBS-LRR CDS in Fasta format. See Section 12.1.

S18 Sequences of all translated NBS CDS in Fasta format. See Section 12.1.

S19 cDNA sequences of manually annotated gluten genes. See Section 12.2.

S20 Coding sequences of manually annotated gluten genes. See Section 12.2.

S21 Protein sequences of manually annotated gluten genes. See Section 12.2.

S22 Gene position and structure of manually annotated gluten genes. See Section 12.2.

S23 Summary of manual gluten gene annotation. See Section 12.2.

S24 Sequences of manually annotated gluten pseudogenes. See Section 12.2.

S25 Sequence of scaffold 577042 of the TGACv1 assembly containing resistance genes. See Section 12.4.

S26 Sequence of scaffold 080625 of the TGACv1 assembly containing gluten genes. See Section 12.4.

15 References

Allouis, S., Moore, G., Bellec, A., Sharp, R., Rampant, P. F., Mortimer, K., Pateyron, S., Foote, T., Griffiths, S., Caboche, M., *et al.*, 2003. Construction and characterisation of a hexaploid wheat (*Triticum aestivum* L.) BAC library from the reference germplasm 'Chinese Spring'. *Cereal Research Communications*, **31**(3/4):331–338.

BabrahamLab, 2014. Trim Galore.

Barrero, J. M., Cavanagh, C., Verbyla, K. L., Tibbits, J. F., Verbyla, A. P., Huang, B. E., Rosewarne, G. M., Stephen, S., Wang, P., Whan, A., *et al.*, 2015. Transcriptomic analysis of wheat near-isogenic lines identifies PM19-A1 and A2 as candidates for a major dormancy QTL. *Genome Biology*, **16**(1):93.

Borrill, P., Ramirez-Gonzalez, R., and Uauy, C., 2016. expVIP: a Customizable RNA-seq Data Analysis and Visualization Platform. *Plant Physiology*, **170**(4):2172–2186.

Bray, N. L., Pimentel, H., Melsted, P., and Pachter, L., 2016. Near-optimal probabilistic RNA-seq quantification. *Nature Biotechnology*, **34**(5):525–527.

Cantu, D., Pearce, S. P., Distelfeld, A., Christiansen, M. W., Uauy, C., Akhunov, E., Fahima, T., and Dubcovsky, J., 2011. Effect of the down-regulation of the high Grain Protein Content (GPC) genes on the wheat transcriptome during monocarpic senescence. *BMC Genomics*, **12**(1):492.

Cantu, D., Segovia, V., MacLean, D., Bayles, R., Chen, X., Kamoun, S., Dubcovsky, J., Saunders, D. G., and Uauy, C., 2013. Genome analyses of the wheat yellow (stripe) rust pathogen Puccinia striiformis f. sp. tritici reveal polymorphic and haustorial expressed secreted proteins as candidate effectors. *BMC Genomics*, **14**(1):270.

Chapman, J. A., Mascher, M., Buluç, A., Barry, K., Georganas, E., Session, A., Strnadova, V., Jenkins, J., Sehgal, S., Oliker, L., *et al.*, 2015. A whole-genome shotgun approach for assembling and anchoring the hexaploid bread wheat genome. *Genome Biology*, **16**(1):26.

Choulet, F., Alberti, A., Theil, S., Glover, N., Barbe, V., Daron, J., Pingault, L., Sourdille, P., Couloux, A., Paux, E., *et al.*, 2014. Structural and functional partitioning of bread wheat chromosome 3B. *Science*, **345**(6194):1249721–1249721.

Clavijo, B., Garcia Accinelli, G., Wright, J., Heavens, D., Barr, K., Yanes, L., and Di Palma, F., 2017. W2RAP: a pipeline for high quality, robust assemblies of large complex genomes from short read data. *bioRxiv*, **10.1101/110999**.

Deutsch, E. W., Mendoza, L., Shteynberg, D., Farrah, T., Lam, H., Tasman, N., Sun, Z., Nilsson, E., Pratt, B., Prazen, B., *et al.*, 2010. A guided tour of the Trans-Proteomic Pipeline. *PROTEOMICS*, **10**(6):1150–1159.

Dobin, A., Davis, C. A., Schlesinger, F., Drenkow, J., Zaleski, C., Jha, S., Batut, P., Chaisson, M., and Gingeras, T. R., 2013. STAR: ultrafast universal RNA-seq aligner. *Bioinformatics*, **29**(1):15–21.

Duncan, O., Trösch, J., Fenske, R., Taylor, N. L., and Millar, A. H., 2017. Resource: Mapping the Triticum aestivum proteome. *The Plant Journal*, **89**(3):601–616.

Eddy, S. R., 2011. Accelerated Profile HMM Searches. *PLoS computational biology*, **7**(10):e1002195.

EdicoGenome, 2014. Dragen Bio-IT processor.

Ellinghaus, D., Kurtz, S., and Willhoeft, U., 2008. LTRharvest, an efficient and flexible software for de novo detection of LTR retrotransposons. *BMC bioinformatics*, **9**:18.

Eng, J. K., Jahan, T. A., and Hoopmann, M. R., 2013. Comet: An open-source MS/MS sequence database search tool. *PROTEOMICS*, **13**(1):22–24.

Falcon, S. and Gentleman, R., 2007. Using GOstats to test gene lists for GO term association. *Bioinformatics*, **23**(2):257–258.

Fernandez, N. and Guerrero, D., 2012. Full Lengther Next.

Frith, M. C., Hamada, M., and Horton, P., 2010. Parameters for accurate genome alignment. *BMC Bioinformatics*, **11**(1):80.

Gillies, S. A., Futardo, A., and Henry, R. J., 2012. Gene expression in the developing aleurone and starchy endosperm of wheat. *Plant Biotechnology Journal*, **10**(6):668–679.

Goodstein, D. M., Shu, S., Howson, R., Neupane, R., Hayes, R. D., Fazo, J., Mitros, T., Dirks, W., Hellsten, U., Putnam, N., *et al.*, 2012. Phytozome: a comparative platform for green plant genomics. *Nucleic Acids Research*, **40**(D1):D1178–D1186.

Grabherr, M. G., Haas, B. J., Yassour, M., Levin, J. Z., Thompson, D. A., Amit, I., Adiconis, X., Fan, L., Raychowdhury, R., Zeng, Q., *et al.*, 2011. Full-length transcriptome assembly from RNA-Seq data without a reference genome. *Nature Biotechnology*, **29**(7):644–652.

Haas, B. J., 2003. Improving the Arabidopsis genome annotation using maximal transcript alignment assemblies. *Nucleic Acids Research*, **31**(19):5654–5666.

Haas, B. J., 2010. TransposonPSI.

Haas, B. J., Papanicolaou, A., Yassour, M., Grabherr, M., Blood, P. D., Bowden, J., Couger, M. B., Eccles, D., Li, B., Lieber, M., *et al.*, 2013. De novo transcript sequence reconstruction from RNA-seq using the Trinity platform for reference generation and analysis. *Nature Protocols*, **8**(8):1494–1512.

Hallab, A., Klee, K., Boecker, F., Girish, S., and Schoof, H., 2014. Automated assignment of Humand Readable Descriptions (AHRD).

Heavens, D., Accinelli, G. G., Clavijo, B., and Clark, M. D., 2015. A method to simultaneously construct up to 12 differently sized Illumina Nextera long mate pair libraries with reduced DNA input, time, and cost. *BioTechniques*, **59**(1):42–45.

Hirano, K., Aya, K., Hobo, T., Sakakibara, H., Kojima, M., Shim, R. A., Hasegawa, Y., Ueguchi-Tanaka, M., and Matsuoka, M., 2008. Comprehensive Transcriptome Analysis of Phytohormone Biosynthesis and Signaling Genes in Microspore/Pollen and Tapetum of Rice. *Plant and Cell Physiology*, **49**(10):1429–1450.

Jones, E., Oliphant, T., Peterson, P., and Others, 2001. SciPy: Open source scientific tools for Python.

Jones, P., Binns, D., Chang, H.-Y., Fraser, M., Li, W., McAnulla, C., McWilliam, H., Maslen, J., Mitchell, A., Nuka, G., *et al.*, 2014. InterProScan 5: genome-scale protein function classification. *Bioinformatics*, **30**(9):1236–1240.

Jupe, F., Pritchard, L., Etherington, G. J., MacKenzie, K., Cock, P. J., Wright, F., Sharma, S. K., Bolser, D., Bryan, G. J., Jones, J. D., *et al.*, 2012. Identification and localisation of the NB-LRR gene family within the potato genome. *BMC Genomics*, **13**(1):75.

Kent, W. J., Baertsch, R., Hinrichs, A., Miller, W., and Haussler, D., 2003. Evolution's cauldron: Duplication, deletion, and rearrangement in the mouse and human genomes. *Proceedings of the National Academy of Sciences*, **100**(20):11484–11489.

Kim, D., Pertea, G., Trapnell, C., Pimentel, H., Kelley, R., and Salzberg, S. L., 2013. TopHat2: accurate alignment of transcriptomes in the presence of insertions, deletions and gene fusions. *Genome Biology*, **14**(4):R36.

Kong, L., Zhang, Y., Ye, Z.-Q., Liu, X.-Q., Zhao, S.-Q., Wei, L., and Gao, G., 2007. CPC: assess the protein-coding potential of transcripts using sequence features and support vector machine. *Nucleic Acids Research*, **35**(Web Server issue):W345–9.

Kopylova, E., Noe, L., and Touzet, H., 2012. SortMeRNA: fast and accurate filtering of ribosomal RNAs in metatranscriptomic data. *Bioinformatics*, **28**(24):3211–3217.

Kugler, K. G., Siegwart, G., Nussbaumer, T., Ametz, C., Spannagl, M., Steiner, B., Lemmens, M., Mayer, K. F., Buerstmayr, H., and Schweiger, W., *et al.*, 2013. Quantitative trait loci-dependent analysis of a gene co-expression network associated with Fusarium head blight resistance in bread wheat (Triticum aestivum L.). *BMC Genomics*, **14**(1):728.

Lamesch, P., Berardini, T. Z., Li, D., Swarbreck, D., Wilks, C., Sasidharan, R., Muller, R., Dreher, K., Alexander, D. L., Garcia-Hernandez, M., *et al.*, 2012. The Arabidopsis Information Resource (TAIR): improved gene annotation and new tools. *Nucleic Acids Research*, **40**(D1):D1202–D1210.

Langmead, B. and Salzberg, S. L., 2012. Fast gapped-read alignment with Bowtie 2. *Nature Methods*, **9**(4):357–359.

Leach, L. J., Belfield, E. J., Jiang, C., Brown, C., Mithani, A., and Harberd, N. P., 2014. Patterns of homoeologous gene expression shown by RNA sequencing in hexaploid bread wheat. *BMC Genomics*, **15**(1):276.

Leggett, R. M., Clavijo, B. J., Clissold, L., Clark, M. D., and Caccamo, M., 2014. NextClip: an analysis and read preparation tool for Nextera Long Mate Pair libraries. *Bioinformatics*, **30**(4):566–568.

Li, A., Liu, D., Wu, J., Zhao, X., Hao, M., Geng, S., Yan, J., Jiang, X., Zhang, L., Wu, J., *et al.*, 2014. mRNA and Small RNA Transcriptomes Reveal Insights into Dynamic Homoeolog Regulation of Allopolyploid Heterosis in Nascent Hexaploid Wheat. *The Plant cell*, **26**(5):1878–1900.

Li, H.-Z., Gao, X., Li, X.-Y., Chen, Q.-J., Dong, J., and Zhao, W.-C., 2013. Evaluation of Assembly Strategies Using RNA-Seq Data Associated with Grain Development of Wheat (Triticum aestivum L.). *PLoS ONE*, **8**(12):e83530.

Li, L., 2003. OrthoMCL: Identification of Ortholog Groups for Eukaryotic Genomes. *Genome Research*, **13**(9):2178–2189.

Liu, Z., Xin, M., Qin, J., Peng, H., Ni, Z., Yao, Y., and Sun, Q., 2015. Temporal transcriptome profiling reveals expression partitioning of homeologous genes contributing to heat and drought acclimation in wheat (Triticum aestivum L.). *BMC Plant Biology*, **15**(1):152.

Luo, R., Liu, B., Xie, Y., Li, Z., Huang, W., Yuan, J., He, G., Chen, Y., Pan, Q., Liu, Y., *et al.*, 2012. SOAPdenovo2: an empirically improved memory-efficient short-read de novo assembler. *GigaScience*, **1**(1):18.

Magoc, T. and Salzberg, S. L., 2011. FLASH: fast length adjustment of short reads to improve genome assemblies. *Bioinformatics*, **27**(21):2957–2963.

Mapleson, D., Garcia Accinelli, G., Kettleborough, G., Wright, J., and Clavijo, B. J., 2017. Kat: a k-mer analysis toolkit to quality control ngs datasets and genome assemblies. *Bioinformatics*, **33**(4):574.

Mapleson, D. L., Venturini, L., and Swarbreck, D., 2016. Portcullis. `https://github.com/maplesond/portcullis`.

Morgan, M., Falcon, S., and Gentleman, R., 2008. *GSEABase: Gene set enrichment data structures and methods*.

Oono, Y., Kobayashi, F., Kawahara, Y., Yazawa, T., Handa, H., Itoh, T., and Matsumoto, T., 2013. Characterisation of the wheat (triticum aestivum L.) transcriptome by de novo assembly for the discovery of phosphate starvation-responsive genes: gene expression in Pi-stressed wheat. *BMC Genomics*, **14**(1):77.

Pearce, S., Huttly, A. K., Prosser, I. M., Li, Y.-d., Vaughan, S. P., Gallova, B., Patil, A., Coghill, J. A., Dubcovsky, J., Hedden, P., *et al.*, 2015. Heterologous expression and transcript analysis of gibberellin biosynthetic genes of grasses reveals novel functionality in the GA3ox family. *BMC Plant Biology*, **15**(1):130.

Pertea, M., Pertea, G. M., Antonescu, C. M., Chang, T.-C., Mendell, J. T., and Salzberg, S. L., 2015. StringTie enables improved reconstruction of a transcriptome from RNA-seq reads. *Nature Biotechnology*, **33**(3):290–295.

Pfeifer, M., Kugler, K. G., Sandve, S. R., Zhan, B., Rudi, H., Hvidsten, T. R., The International Wheat Genome Sequencing Consortium (IWGSC), Mayer, K. F. X., and Olsen, O.-A., 2014. Genome interplay in the grain transcriptome of hexaploid bread wheat. *Science*, **345**(6194):1250091.

Pimentel, H. J., Bray, N., Puente, S., Melsted, P., and Pachter, L., 2016. Differential analysis of RNA-Seq incorporating quantification uncertainty. *Preprint*, .

Ramirez-Gonzalez, R. H., Uauy, C., and Caccamo, M., 2015. PolyMarker: A fast polyploid primer design pipeline: Fig. 1. *Bioinformatics*, **31**(12):2038–2039.

Roberts, A., Pimentel, H., Trapnell, C., and Pachter, L., 2011a. Identification of novel transcripts in annotated genomes using RNA-Seq. *Bioinformatics*, **27**(17):2325–2329.

Roberts, A., Trapnell, C., Donaghey, J., Rinn, J. L., and Pachter, L., 2011b. Improving RNA-Seq expression estimates by correcting for fragment bias. *Genome Biology*, **12**(3):R22.

Sambrook, J. and Russell, D. W., 2006. Preparation of Plasmid DNA by Alkaline Lysis with SDS: Minipreparation. *CSH protocols*, **2006**(1).

Sarris, P. F., Cevik, V., Dagdas, G., Jones, J. D. G., and Krasileva, K. V., 2016. Comparative analysis of plant immune receptor architectures uncovers host proteins likely targeted by pathogens. *BMC Biology*, **14**(1):8.

Seabold, S. and Perktold, J., 2010. Statsmodels: Econometric and statistical modeling with python. In *Proceedings of the 9th Python in Science Conference*, pages 57–61.

Sears, E. R., 1966. Nullisomic-Tetrasomic Combinations in Hexaploid Wheat. In *Chromosome Manipulations and Plant Genetics*, pages 29–45. Springer US, Boston, MA.

Slater, G. S. C. and Birney, E., 2005. Automated generation of heuristics for biological sequence comparison. *BMC bioinformatics*, **6**(1):31.

Song, L., Sabunciyan, S., and Florea, L., 2016. CLASS2: accurate and efficient splice variant annotation from RNA-seq reads. *Nucleic Acids Research*, **44**(10):e98–e98.

Spannagl, M., Nussbaumer, T., Bader, K. C., Martis, M. M., Seidel, M., Kugler, K. G., Gundlach, H., and Mayer, K. F., 2016. PGSB PlantsDB: updates to the database framework for comparative plant genome research. *Nucleic Acids Research*, **44**(D1):D1141–D1147.

Stanke, M., Tzvetkova, A., and Morgenstern, B., 2006. AUGUSTUS at EGASP: using EST, protein and genomic alignments for improved gene prediction in the human genome. *Genome biology*, **7 Suppl 1**(May 2005):S11.1–8.

Steuernagel, B., Jupe, F., Witek, K., Jones, J. D. G., and Wulff, B. B. H., 2015. NLR-parser: rapid annotation of plant NLR complements. *Bioinformatics*, **31**(10):1665–1667.

Sturgill, D., Malone, J. H., Sun, X., Smith, H. E., Rabinow, L., Samson, M.-L., and Oliver, B., 2013. Design of RNA splicing analysis null models for post hoc filtering of Drosophila head RNA-Seq data with the splicing analysis kit (Spanki). *BMC Bioinformatics*, **14**(1):320.

Supek, F., Bošnjak, M., Škunca, N., and Šmuc, T., 2011. REVIGO summarizes and visualizes long lists of gene ontology terms. *PloS one*, **6**(7):e21800.

The UniProt Consortium, 2014. Activities at the Universal Protein Resource (UniProt). *Nucleic Acids Research*, **42**(D1):D191–D198.

The International Wheat Genome Sequencing Consortium, 2014. A chromosome-based draft sequence of the hexaploid bread wheat (Triticum aestivum) genome. *Science*, **345**(6194):1251788–1251788.

Trapnell, C., Hendrickson, D. G., Sauvageau, M., Goff, L., Rinn, J. L., and Pachter, L., 2012. Differential analysis of gene regulation at transcript resolution with RNA-seq. *Nature Biotechnology*, **31**(1):46–53.

Trapnell, C., Williams, B. A., Pertea, G., Mortazavi, A., Kwan, G., van Baren, M. J., Salzberg, S. L., Wold, B. J., and Pachter, L., 2010. Transcript assembly and quantification by RNA-Seq reveals unannotated transcripts and isoform switching during cell differentiation. *Nature Biotechnology*, **28**(5):511–515.

Venturini, L., Caim, S., Mapleson, D. L., Kaithakottil, G. G., and Swarbreck, D., 2016. Mikado. `https://github.com/lucventurini/mikado`.

Vitting-Seerup, K., Porse, B., Sandelin, A., and Waage, J., 2014. spliceR: an R package for classification of alternative splicing and prediction of coding potential from RNA-seq data. *BMC Bioinformatics*, **15**(1):81.

Wagner, G. P., Kin, K., and Lynch, V. J., 2013. A model based criterion for gene expression calls using RNA-seq data. *Theory in Biosciences*, **132**(3):159–164.

Wang, L., Wang, S., and Li, W., 2012. RSeQC: quality control of RNA-seq experiments. *Bioinformatics*, **28**(16):2184–2185.

Weisenfeld, N. I., Yin, S., Sharpe, T., Lau, B., Hegarty, R., Holmes, L., Sogoloff, B., Tabbaa, D., Williams, L., Russ, C., *et al.*, 2014. Comprehensive variation discovery in single human genomes. *Nature Genetics*, **46**(12):1350–1355.

Wessel, D. and Flügge, U., 1984. A method for the quantitative recovery of protein in dilute solution in the presence of detergents and lipids. *Analytical Biochemistry*, **138**(1):141–143.

Wu, T. D. and Watanabe, C. K., 2005. GMAP: a genomic mapping and alignment program for mRNA and EST sequences. *Bioinformatics*, **21**(9):1859–1875.

Wysokar, A., Tibbetts, K., McCown, M., Homer, N., and Fennell, T., 2016. Picard: A set of Java command line tools for manipulating high-throughput sequencing data (HTS) data and formats.

Yang, F., Li, W., and Jørgensen, H. J. L., 2013. Transcriptional Reprogramming of Wheat and the Hemibiotrophic Pathogen Septoria tritici during Two Phases of the Compatible Interaction. *PLoS ONE*, **8**(11):e81606.

Yang, F., Shen, Y., Camp, D. G., and Smith, R. D., 2012. High-pH reversed-phase chromatography with fraction concatenation for 2D proteomic analysis. *Expert Review of Proteomics*, **9**(2):129–134.

Yang, Z., Peng, Z., Wei, S., Liao, M., Yu, Y., and Jang, Z., 2015. Pistillody mutant reveals key insights into stamen and pistil development in wheat (Triticum aestivum L.). *BMC Genomics*, **16**(1):211.

Zhang, H., Yang, Y., Wang, C., Liu, M., Li, H., Fu, Y., Wang, Y., Nie, Y., Liu, X., and Ji, W., *et al.*, 2014a. Large-scale transcriptome comparison reveals distinct gene activations in wheat responding to stripe rust and powdery mildew. *BMC Genomics*, **15**(1):898.

Zhang, W., Ciclitira, P., and Messing, J., 2014b. PacBio sequencing of gene families - a case study with wheat gluten genes. *Gene*, **533**(2):541–6.
